# Identification of Key Pathways and Genes in Obesity Using Bioinformatics Analysis and Molecular Docking Studies

**DOI:** 10.3389/fendo.2021.628907

**Published:** 2021-06-24

**Authors:** Harish Joshi, Basavaraj Vastrad, Nidhi Joshi, Chanabasayya Vastrad, Anandkumar Tengli, Iranna Kotturshetti

**Affiliations:** ^1^ Department of Endocrinology, Endocrine and Diabetes Care Center, Hubbali, India; ^2^ Department of Biochemistry, Basaveshwar College of Pharmacy, Gadag, India; ^3^ Department of Medicine, Dr. D. Y. Patil Medical College, Kolhapur, India; ^4^ Biostatistics and Bioinformatics, Chanabasava Nilaya, Bharthinagar, Dharwad, India; ^5^ Department of Pharmaceutical Chemistry, JSS College of Pharmacy, Mysuru and JSS Academy of Higher Education & Research, Mysuru, India; ^6^ Department of Ayurveda, Rajiv Gandhi Education Society`s Ayurvedic Medical College, Ron, India

**Keywords:** adiposities, obesity, differentially expressed genes, modules, protein–protein interaction network

## Abstract

Obesity is an excess accumulation of body fat. Its progression rate has remained high in recent years. Therefore, the aim of this study was to diagnose important differentially expressed genes (DEGs) associated in its development, which may be used as novel biomarkers or potential therapeutic targets for obesity. The gene expression profile of E-MTAB-6728 was downloaded from the database. After screening DEGs in each ArrayExpress dataset, we further used the robust rank aggregation method to diagnose 876 significant DEGs including 438 up regulated and 438 down regulated genes. Functional enrichment analysis was performed. These DEGs were shown to be significantly enriched in different obesity related pathways and GO functions. Then protein–protein interaction network, target genes - miRNA regulatory network and target genes - TF regulatory network were constructed and analyzed. The module analysis was performed based on the whole PPI network. We finally filtered out STAT3, CORO1C, SERPINH1, MVP, ITGB5, PCM1, SIRT1, EEF1G, PTEN and RPS2 hub genes. Hub genes were validated by ICH analysis, receiver operating curve (ROC) analysis and RT-PCR. Finally a molecular docking study was performed to find small drug molecules. The robust DEGs linked with the development of obesity were screened through the expression profile, and integrated bioinformatics analysis was conducted. Our study provides reliable molecular biomarkers for screening and diagnosis, prognosis as well as novel therapeutic targets for obesity.

## Introduction

Obesity has long been part of the larger metabolic disorder and affects a large proportion of the global population particularly in the Western World (1). Obesity is diagnosed on the basis of body mass index (1). Obesity occurs in children age between 5 to 19 years as well as more common in women than in men (2). Countless surveys have proved that obesity is an key risk factor for heart disease (3), hyperlipidaemia (4), hyperinsulinaemia (5), hypertension (6), atherosclerosis (7), insulin resistance (8) and cancer (9). Important candidate genes and relevant signaling pathways linked with obesity remains largely unknown. As a result, seek of an earlier diagnosis and better prognosis, deeper understanding of genetic and molecular mechanisms about obesity is necessary.

Previous reports demonstrate that many genes and signaling pathways participate in obesity. Polymorphisms in UCP2 and UCP3 were responsible for development of obesity (10). TNFα and lipoprotein lipase were important for advancement of obesity (11). SLC6A14 (12) and JHDM2A (13) were lined with pathogenesis of obesity. Human salivary (AMY1) and pancreatic (AMY2) amylase genes were diagnosed with growth of obesity (14). Signaling pathways such as inflammatory signaling pathway (15), TLR4 signaling pathway (16), calcineurin-dependent signaling pathways (17), mTOR Complex1–S6K1 signaling pathway (18) and leptin-signaling pathway (19) were important for development of obesity. Therefore, it is meaningful to explore the precise molecular mechanisms involved in obesity and thus find a valid diagnostic way and generate an advance therapeutic strategy.

In present trends, the application of high-throughput analysis in gene expression profiling is becoming more valuable in clinical and medical research (20), molecular classification (21), prognosis prediction (22), diagnoses (23) and new targeted drug discovery (24). In this study, the original microarray data (E-MTAB-6728) was downloaded from ArrayExpress database (https://www.ebi.ac.uk/) and analyzed to get differently expressed genes (DEGs) between obesity persons and lean persons (normal controls). Subsequently, gene ontology (GO), pathway enrichment analysis, protein–protein interaction network construction and analysis, module analysis, target gene - miRNA interaction network construction and analysis, and target gene - TF interaction network construction and analysis to discover the key genes and pathways closely related to obesity. Finally, selected hub genes were validated by immunohistochemical (IHC) analysis, receiver operating characteristic curve (ROC) analysis and RT-PCR. This current investigation aimed at using bioinformatics tools to predict the key pathways and genes in obesity that can hold a value for target based therapeutic means.

## Materials and Methods

### Microarray Data

The microarray expression profile of E-MTAB-6728 was downloaded from ArrayExpress (https://www.ebi.ac.uk/). E-MTAB-6728 was based on A-MEXP-1171 - Illumina HumanHT-12 v3.0 Expression BeadChip and was submitted by Bjune et al. (25). The E-MTAB-6728 dataset about expression of genes from obesity persons compared to lean persons (normal controls).There are twenty-four samples including twelve obesity persons and lean persons (normal controls). The overall design of the experiment was microarray analysis of adiposities from obese patients versus adipocytes from lean persons (controls).

### Identification of DEGs

The raw data files were acquired for the analysis as IDAT files (Illumina platform) forms and were converted into gene symbols and then processed to background correction and quantile data normalization using the effective multiarray average algorithm in the beadarray package (26). The analysis was carried out *via* R software (version 3.5.2). Hierarchical clustering analysis was applied to categorize the samples into two groups with similar expression patterns in obesity persons and lean persons (normal controls). The paired Student’s t-test based on the Limma package in R bioconductor was used to diagnose DEGs between two experimental conditions (27). Multiple testing corrections were performed by the Benjamini–Hochberg method (28). Then, the Log2 Fold change (log_2_FC) was determined. We selected up regulated DEGs with | log_2_FC | > 0.524 and FDR < 0.05, and down regulated DEGs with | log_2_FC | < -0.394 and FDR < 0.05 were considered as the cutoff values.

### Pathway and Gene Ontology (GO) Enrichment Analysis of DEGs

The BIOCYC, Kyoto Encyclopedia of Genes and Genomes (KEGG), REACTOME, Pathway Interaction Database (PID), GenMAPP, MSigDB C2 BIOCARTA, PantherDB, Pathway Ontology and Small Molecule Pathway Database (SMPDB) databases are a knowledge base for systematic analysis, annotation, and visualization of gene functions. The GO database can add functional classification for genomic data, including categories of biological processes (BP), cellular component (CC), and molecular function (MF). GO analysis is a prevalent genes and gene products annotating approach. ToppCluster (https://toppcluster.cchmc.org/) (29) is an online tool for gene functional classification, which is a key foundation for high-throughput gene analysis to understand the biological importance of genes. In the current investigation, in order to analyze the functions of DEGs, Pathway and GO enrichment analysis were conducted using the ToppCluster online tool; p<0.05 was set as the cutoff point.

### Integration of PPI Network and Module Analysis

The mentha (https://mentha.uniroma2.it/) (30) is a biological database designed to predict protein-protein interaction (PPI) information. The DEGs were mapped to STRING to evaluate the interactive relationships, with a confidence score >0.9 defined as significant. Then, integration of protein-protein interaction (PPI) network was visualized using cytoscape software (version 3.8.2) (http://www.cytoscape.org/) (31). The plug-in Network Analyzer identified hub genes based on mathematical calculation methods such as node degree (32), betweenness (33), stress (34) and closeness (35) the number of genes within centrality mathematical calculation methods were represented the significance of the disorder. The PEWCC1 was applied to screen modules of PPI network with degree cutoff = 2, node score cutoff = 0.2, k-core = 2, and max. depth = 100 (36). The functional enrichment analysis in the module was performed by ToppCluster.

### Construction of Target Genes - miRNA Regulatory Network

MiRNA of target genes were explored combined with the human miRNA information (miRNet database, (https://www.mirnet.ca/) (37), recorded using TarBase, miRTarBase, miRecords, miR2Disease, HMDD, SM2miR, PhenomiR, PharmacomiR, EpimiR and starBase databases, and visualized using the Cytoscape software (31).

### Construction of Target Genes - TF Regulatory Network

TFs of hub genes were explored combined with the human TF information (NetworkAnalyst database, http://www.networkanalyst.ca) (38), recorded using ENCODE database, and visualized using the Cytoscape software (31).

### Validation of Hub Genes

Immunohistochemical (IHC) analysis of adipose tissues was performed utilizing human protein atlas (www.proteinatlas.org) (39). ROC analysis was performed using pROC package (40) in R. ROC analyses were estimated for diagnostic value of hub genes. When the AUC value was > 0.7, the hub genes were considered to be capable of distinguishing obesity persons from normal lean with excellent specificity and sensitivity.

### Detection of the mRNA Expression of the Hub Genes by RT-PCR

D12 (ATCC CRL-3280) cell line for obesity and D16 (ATCC CRL-3281) cell line a normal control were purchased from the American Type Culture Collection (ATCC) (Maryland, USA). D12 cells were cultured in Dulbecco’s modified Eagle’s medium (DMEM) F­12 medium, which contains 10% fetal bovine serum. D16 cells were cultured in Dulbecco’s modified Eagle’s medium (DMEM) F­12 medium, which contains 10% fetal bovine serum. The culture temperature is 37°C and CO2 concentration is 5%. Total cellular RNA was extracted from cell culture with 1 ml TRI Reagent^®^ (Sigma, USA). Reverse transcription cDNA kit (Thermo Fisher Scientific, Waltham, MA, USA) and random primers were used to synthesize cDNA. RT-PCR was performed using QuantStudio 7 Flex real-time PCR system (Thermo Fisher Scientific, Waltham, MA, USA). The conditions for RT-PCR amplification were as follows: 95°C for 120 seconds followed by 40 cycles of 95°C for 15 seconds, annealing temperature for 45 seconds. Each sample was run in triplicate. Relative expression level for each target gene was normalized by the Ct value of β-actin (internal control) using a 2 ^−ΔΔCT^ relative quantification method (41). The primer pairs used in the experiments are listed in **Supplementary Table 1**.

### Molecular Docking Studies

The module SYBYL-X 2.0 perpetual software was used for Surflex-Docking of the designed molecules. The molecules were sketched by using ChemDraw Software and imported and saved in sdf. format using Openbabelfree software. The one co-crystallized protein from each of ERBB2, STAT3 and HSPAB8 were selected for docking studies. The protein structures of ERBB2, STAT3 and HSPAB8 of PDB code 1MFL, 5OOW and 3CWG was retrieved from Protein Data Bank (42–44). Together with the TRIPOS force field, GasteigerHuckel (GH) charges were added to all designed molecules and the standard ant-obesity drug Orlistat, for the structure optimization process. In addition, energy minimization was carried out using MMFF94s and MMFF94 algorithm process. Protein processing was carried out after the incorporation of protein. The co-crystallized ligand and all water molecules were removed from the crystal structure; more hydrogen’s were added and the side chain was set. TRIPOS force field was used for the minimization of structure. The designed molecules interaction efficiency with the receptor was represented by the Surflex-Dock score in kcal/mol units. The interaction between the protein and the ligand, the best pose was incorporated into the molecular area. The visualization of ligand interaction with receptor is done by using discovery studio visualizer.

## Results

### Data Normalization

**Figure 1:**
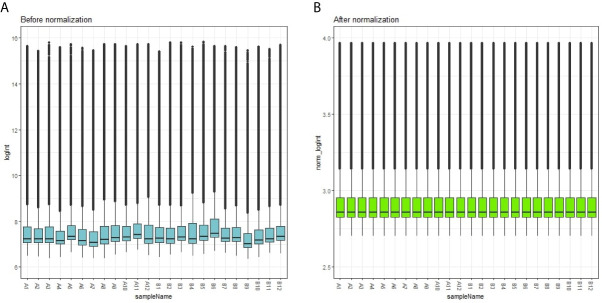
Box plots of the gene expression data before **(A)** and after normalization **(B)**. Horizontal axis represents the sample symbol and the vertical axis represents the gene expression values. The black line in the box plot represents the median value of gene expression. (A1-A12 = adipocytes from lean persons; B1-B12 = adipocytes from obese patients).

Each array was normalized (centered) by quantile data normalization using the beadarray package in R bioconductor. As shown in **Figures 1A, B**, raw expression data were normalized after preprocessing; median-centered values demonstrated that the data were normalized and thus it was possible to cross-compare between obesity persons and lean persons (normal controls).

### Identification of DEGs Between Obese Patients and Lean Persons

**Figure 2:**
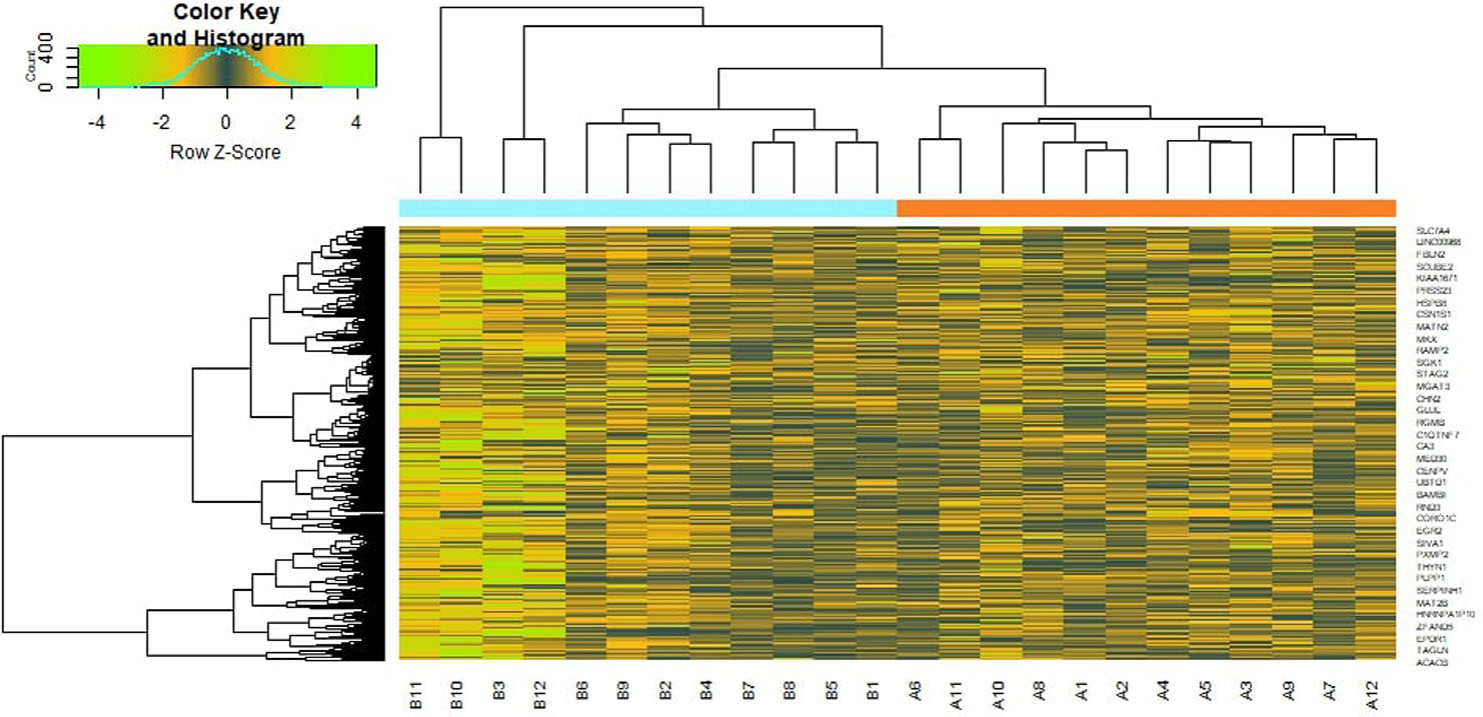
Heat map of differentially expressed genes.

**Figure 3:**
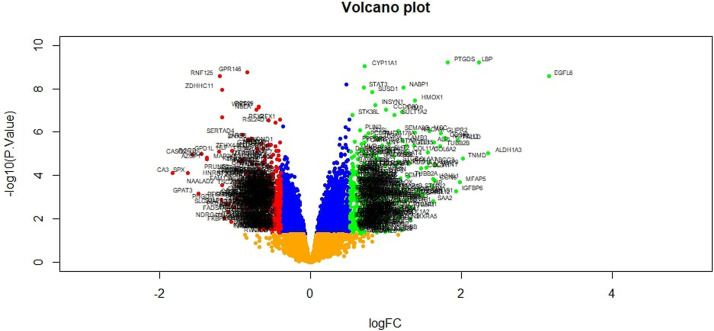
Volcano plot of differentially expressed genes. Genes with a significant change of more than two-fold were selected.

To preliminarily understand the mechanism contributing to the obesity, 24 patients [12 obesity persons and 12 lean persons (normal controls)] were selected for subsequent analysis. Based on the analysis, a total of 876 DEG compose of 438 genes had been expressed highly and about 438 genes had been shown to decrease expression in obesity and are listed in **Supplementary Table 2**. The FDR <0.05 was as a threshold value. Heat map is shown in **Figure 2**. Volcano plot for DEGs is shown in **Figure 3**.

### Pathway and Gene Ontology (GO) Enrichment Analysis of DEGs

To further investigate the biologic functions and mechanisms of the DEGs, pathway and GO enrichment analyses were performed using ToppCluster tool. Pathway enrichment analysis revealed that the up regulated genes were mainly enriched in thyroid hormone metabolism II (via conjugation and/or degradation), ECM-receptor interaction, IL6-mediated signaling events, collagen formation, C21 steroid hormone metabolism, genes encoding collagen proteins, integrin signalling pathway, hypertension and suprofen pathway, and are listed in **Supplementary Table 3**. Similarly, down regulated genes were mainly enriched in superpathway of methionine degradation, ribosome, FoxO family signaling, eukaryotic translation elongation, propanoate metabolism, CDK regulation of DNA replication, p38 MAPK pathway, glycine, serine and threonine metabolic, and glycine, serine and threonine metabolism, and are listed in **Supplementary Table 4**. GO analysis results showed that up regulated genes were significantly enriched in blood vessel morphogenesis, extracellular matrix and growth factor binding, and are listed in **Supplementary Table 5**. Similarly, down regulated genes were mainly enriched in organic acid biosynthetic process, cytosolic small ribosomal subunit and structural constituent of ribosome, and are listed in **Supplementary Table 6**.

### Integration of PPI Network and Module Analysis

**Figure 4:**
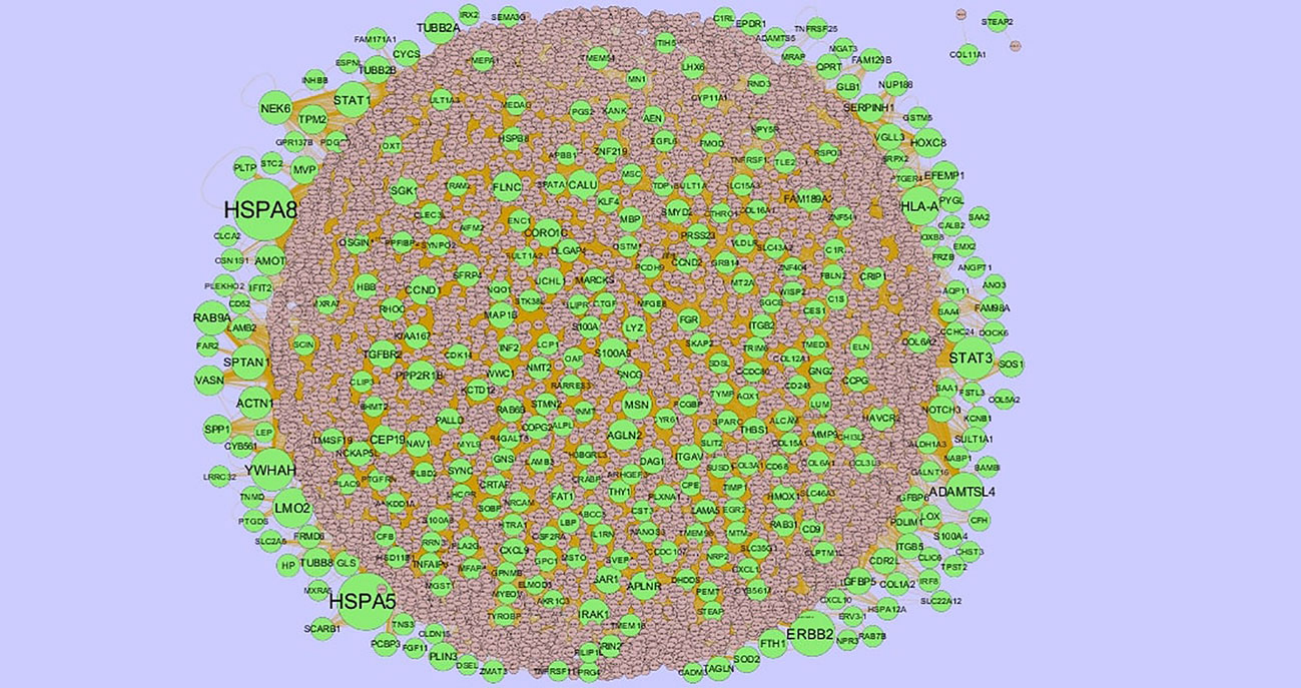
Protein–protein interaction network of up regulated genes. Green nodes denotes up regulated genes.

**Figure 5:**
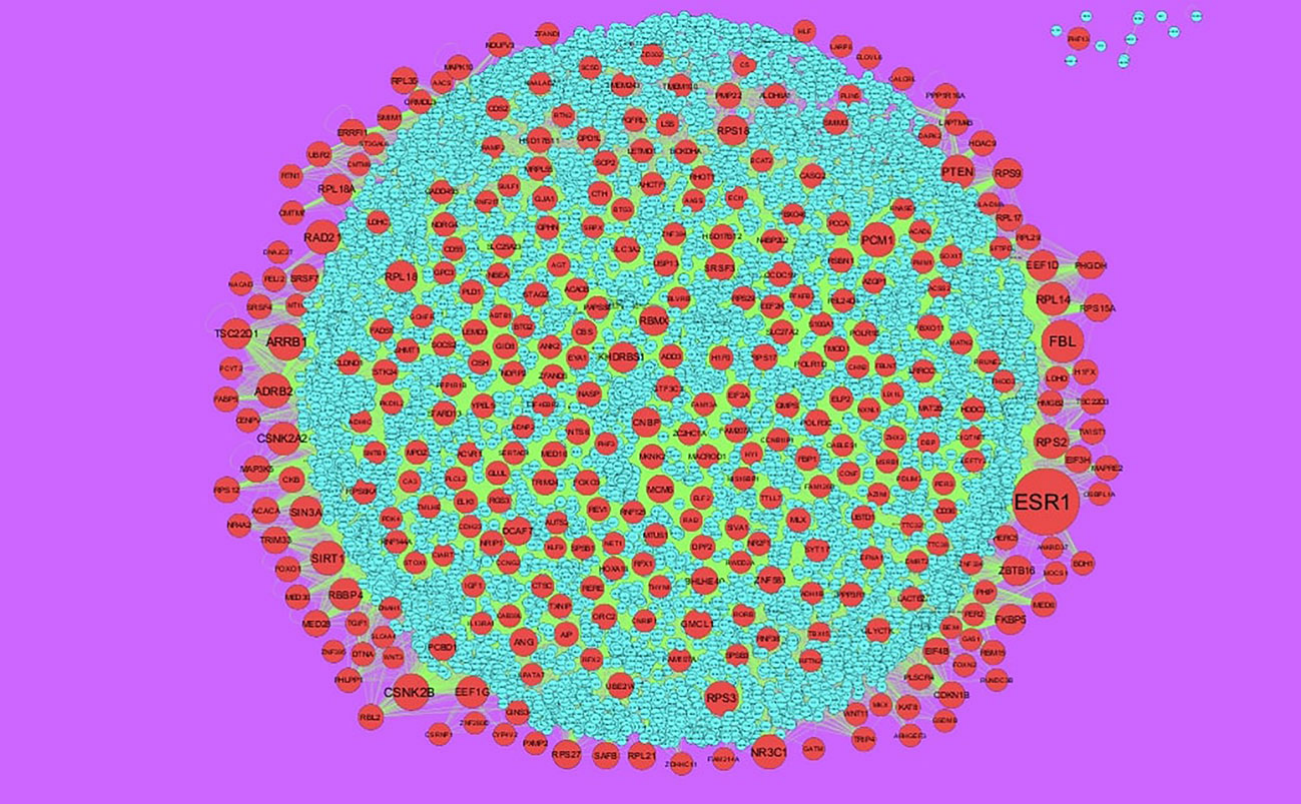
Protein–protein interaction network of down regulated genes.

**Figure 6:**
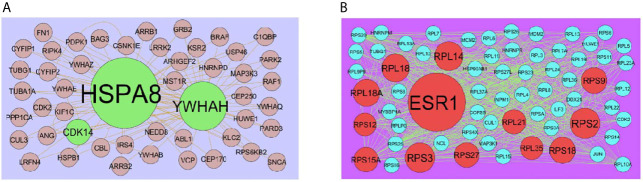
**(A)** Module of up regulated genes. The green nodes denote the up regulated genes **(B)** Module of down regulated genes. The red nodes denote the down regulated genes.

The PPI network of up regulated genes consisted of 7271 nodes and 16270 edges (**Figure 4**) and down regulated genes consisted of 7276 nodes and 19862 edges (**Figure 5**) constructed in the mentha database and visualized using Cytoscape software. Based on the mentha database, the DEGs with the highest PPI scores identified by the four centrality methods are shown in **Supplementary Table 7**. There are 5 up regulated genes selected as hub genes, such as HSPA8, HSPA5, YWHAH, STAT3 and ERBB2, and 5 down regulated genes selected as hub genes, such as ESR1, ARRB1, CSNK2A2, RBBP4 and NR3C1. A significant module was obtained from PPI network of DEGs using PEWCC1, including module 1 contains 49 nodes and 99 edges (**Figure 6A**) and module 2 contains 66 nodes and 754 edges (**Figure 6B**). Functional enrichment analysis revealed that genes in these modules were mainly involved in PI3K-Akt signaling pathway, regulation of nuclear SMAD2/3 signaling, ribosome, eukaryotic translation elongation, metabolism of amino acids and derivatives, disease, cellular amide metabolic process, establishment of protein localization to endoplasmic reticulum, monocarboxylic acid biosynthetic process translation, translational initiation, macromolecule catabolic process and cytosolic small ribosomal subunit.

### Construction of Target Genes - miRNA Regulatory Network

**Figure 7:**
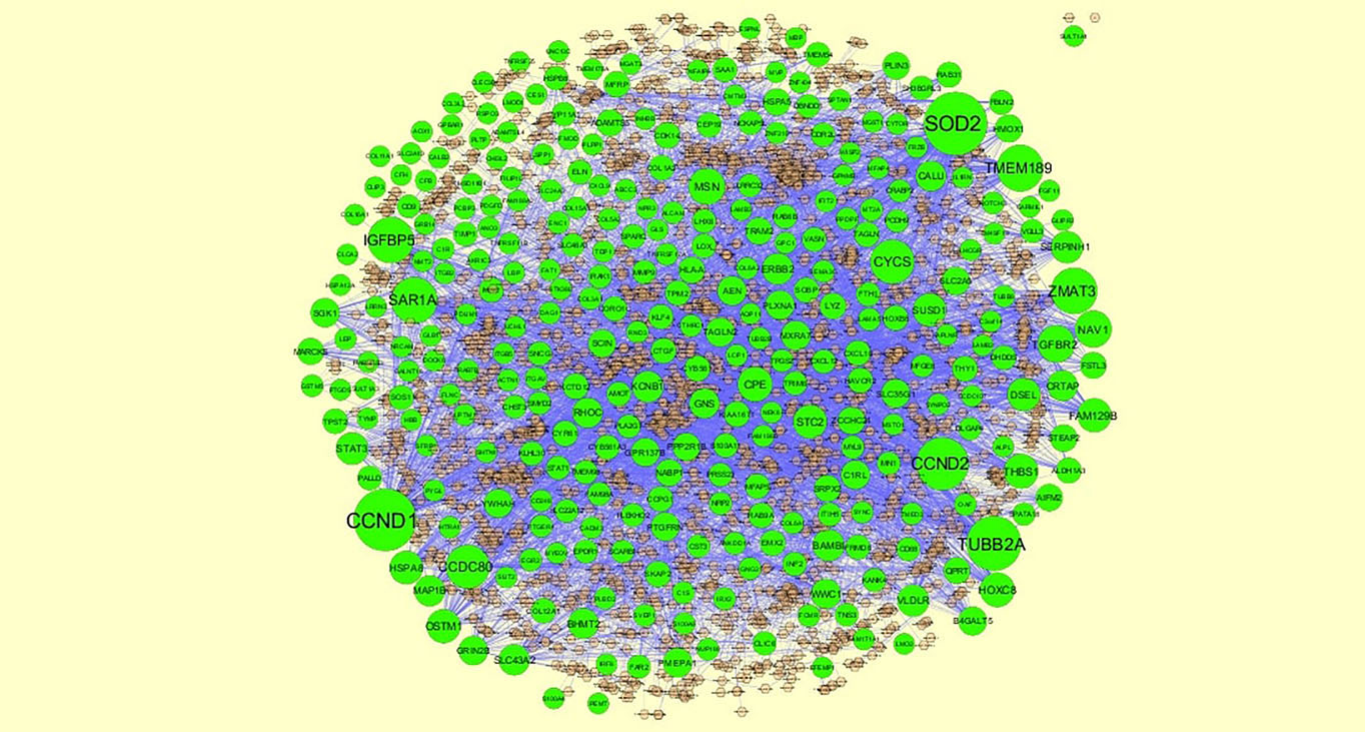
The network of up regulated genes and their related miRNAs. The green circles nodes are the up regulated genes, and chocolate diamond nodes are the miRNAs.

**Figure 8:**
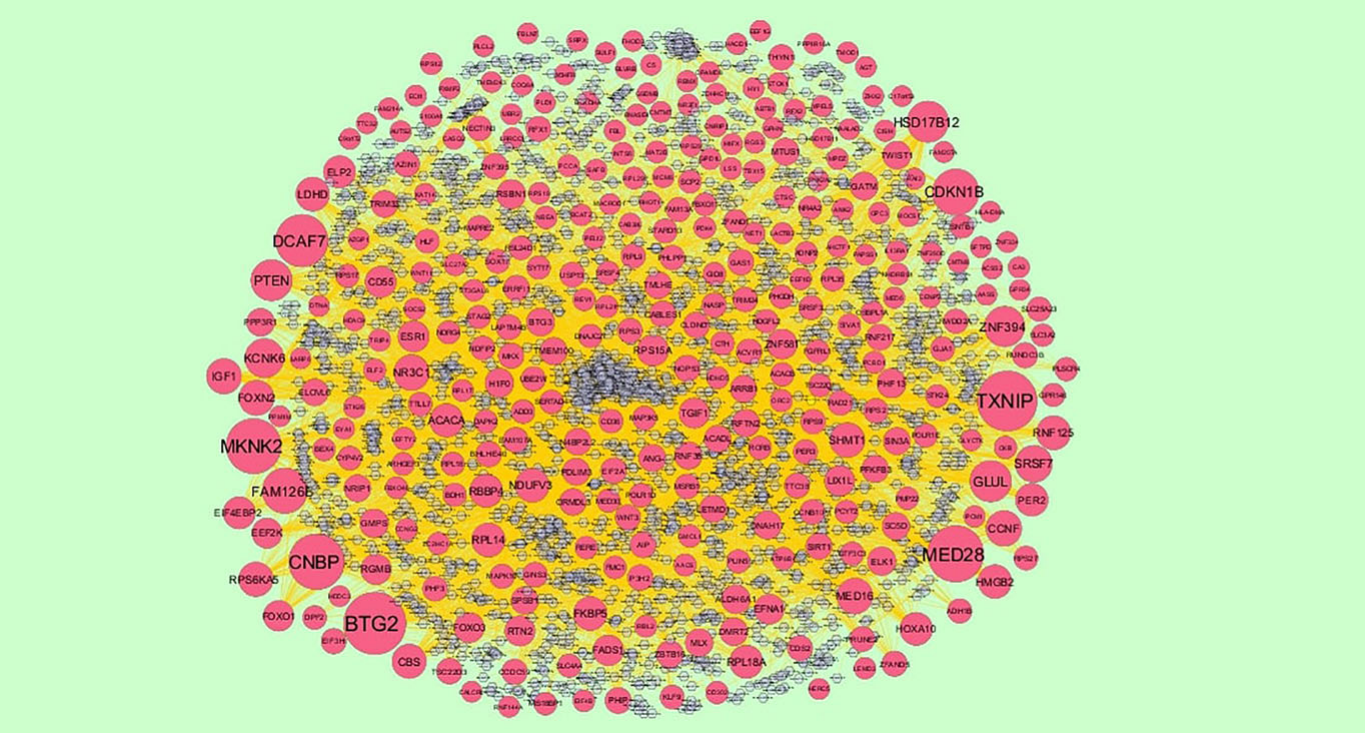
The network of down regulated genes and their related miRNAs. The red circles nodes are the down regulated genes, and chocolate diamond nodes are the miRNAs.

To further understand the regulatory network between miRNAs and target genes, through miRNet database were constructed by Cytoscape. As shown in **Figure 7**, the miRNA-regulated network with 2613 nodes (miRNA: 2261; target gene: 352) and 17260 edges was obtained for up regulated target genes and **Figure 8**, the miRNA-regulated network with 2685 nodes (miRNA: 2327; target gene: 358) and 19827 edges was obtained for down regulated target genes. Different target genes regulated by miRNAs are shown in **Supplementary Table 8**. SOD2 had been predicted to regulate 257 miRNAs (ex; hsa-mir-3144-3p), CCND1 had been predicted to regulate 251 miRNAs (ex; hsa-mir-7706), TUBB2A had been predicted to regulate 193 miRNAs (ex; hsa-mir-5692c), CCND2 had been predicted to regulate 179 miRNAs (ex; hsa-mir-7162-3p), TMEM189 had been predicted to regulate 146 miRNAs (ex; hsa-mir-548z), BTG2 had been predicted to regulate 247 miRNAs (ex; hsa-mir-6075), TXNIP had been predicted to regulate 228 miRNAs (ex; hsa-mir-3194-3p), MED28 had been predicted to regulate 203 miRNAs (ex; hsa-mir-6861-5p), CNBP had been predicted to regulate 197 miRNAs (ex; hsa-mir-4651) and MKNK2 had been predicted to regulate 195 miRNAs (ex; hsa-mir-3650).

### Construction of Target Genes - TF Regulatory Network

**Figure 9:**
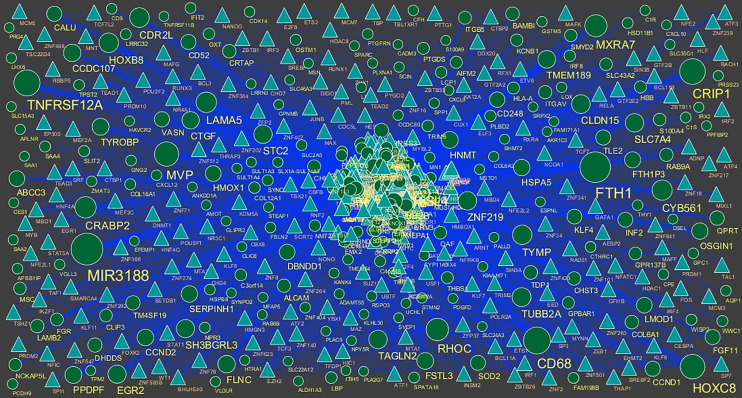
TF ‐ gene network of predicted target up regulated genes. (Blue triangle - TFs and green circles- target up regulated genes).

**Figure 10:**
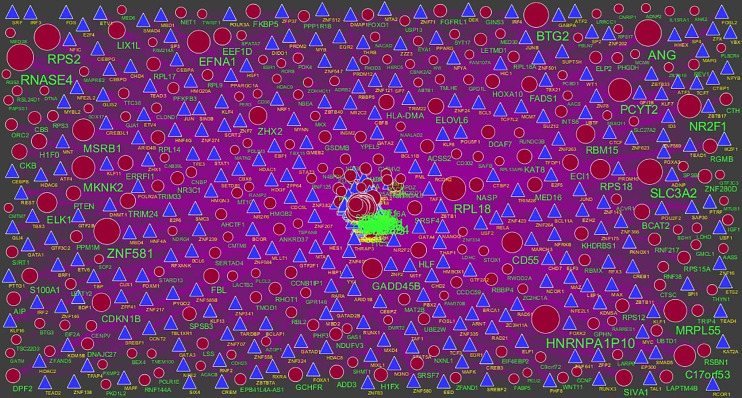
TF‐gene network of predicted target down regulated genes. (Blue triangle - TFs and red circles- target up regulated genes).

To further understand the regulatory network between TFs and target genes, through NetworkAnalyst database were constructed by Cytoscape. As shown in **Figure 9**, the TF-regulated network with 629 nodes (TF: 336; Gene: 293) and 6293 edges was obtained for up regulated target genes and **Figure 10**, the TF-regulated network with 2685 nodes (TF: 342; Gene: 299) and 8597 edges was obtained for down regulated target genes. Different target genes regulated by TFs are shown in **Supplementary Table 9**. YWHAH had been predicted to regulate 70 TFs (ex; MAZ), LYZ had been predicted to regulate 62 TFs (ex; TFDP1), HP had been predicted to regulate 60 TFs (ex; KLF9), TRAM2 had been predicted to regulate 54 TFs (ex; KLF16), CCND1 had been predicted to regulate 51 TFs (ex; EZH2), EFNA1 had been predicted to regulate 91 TFs (ex; TFDP1), MED16 had been predicted to regulate 85 TFs (ex; MAZ), RWDD2A had been predicted to regulate 82 TFs (ex; KDM5B), ADD3 had been predicted to regulate 82 TFs (ex; SAP30) and AIP had been predicted to regulate 82 TFs (ex; PHF8).

### Validation of Hub Genes

**Figure 11:**
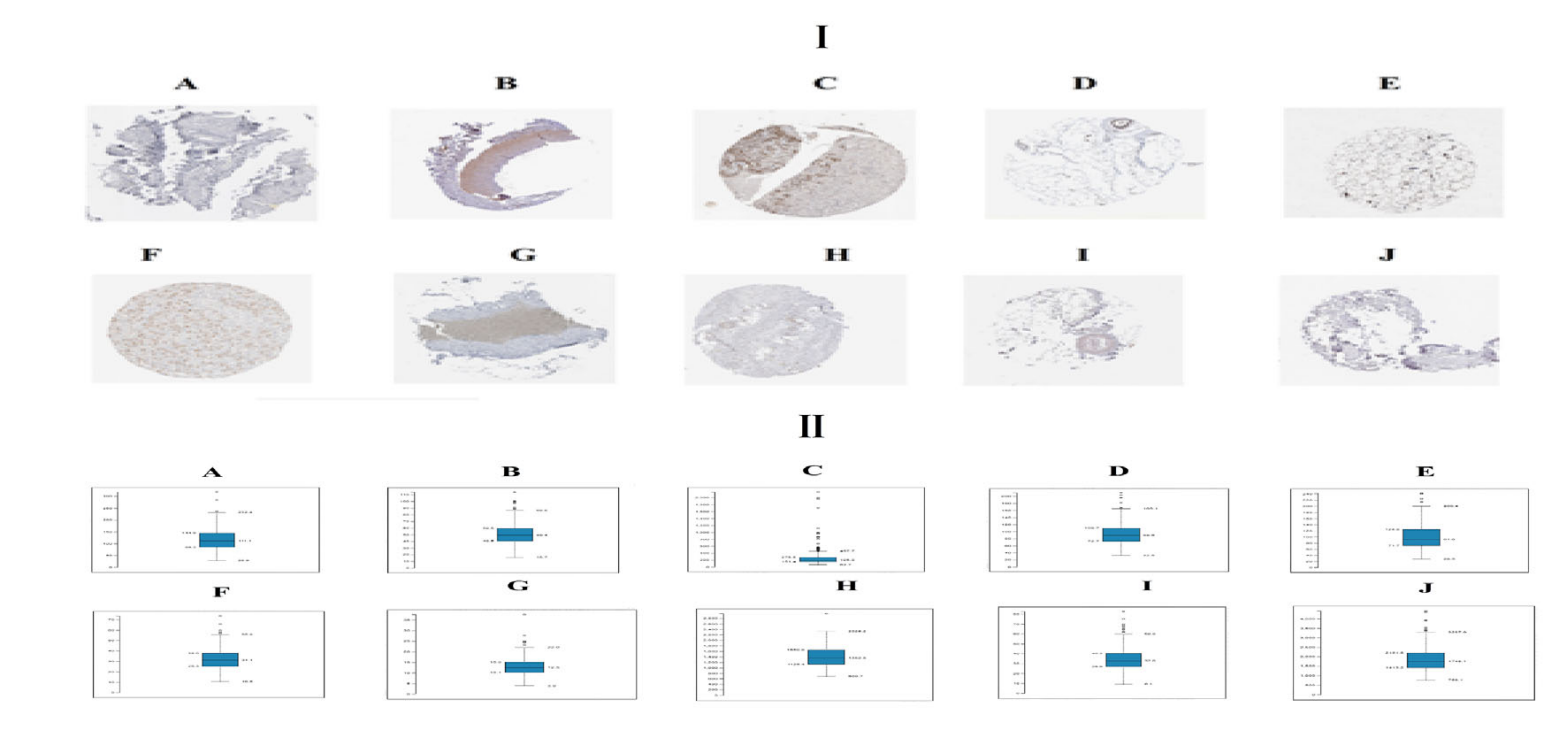
I) Immunohisto chemical l (IHC) analyses of hub genes were produced using the human protein atlas (HPA) online platform. II) Box plot for IHC analysis of hub genes **(A)** STAT3 **(B)** CORO1C **(C)** SERPINH1 **(D)** MVP **(E)** ITGB5 **(F)** PCM1 **(G)** SIRT1 **(H)** EEF1G **(I)** PTEN **(J)** RPS2.

**Figure 12:**
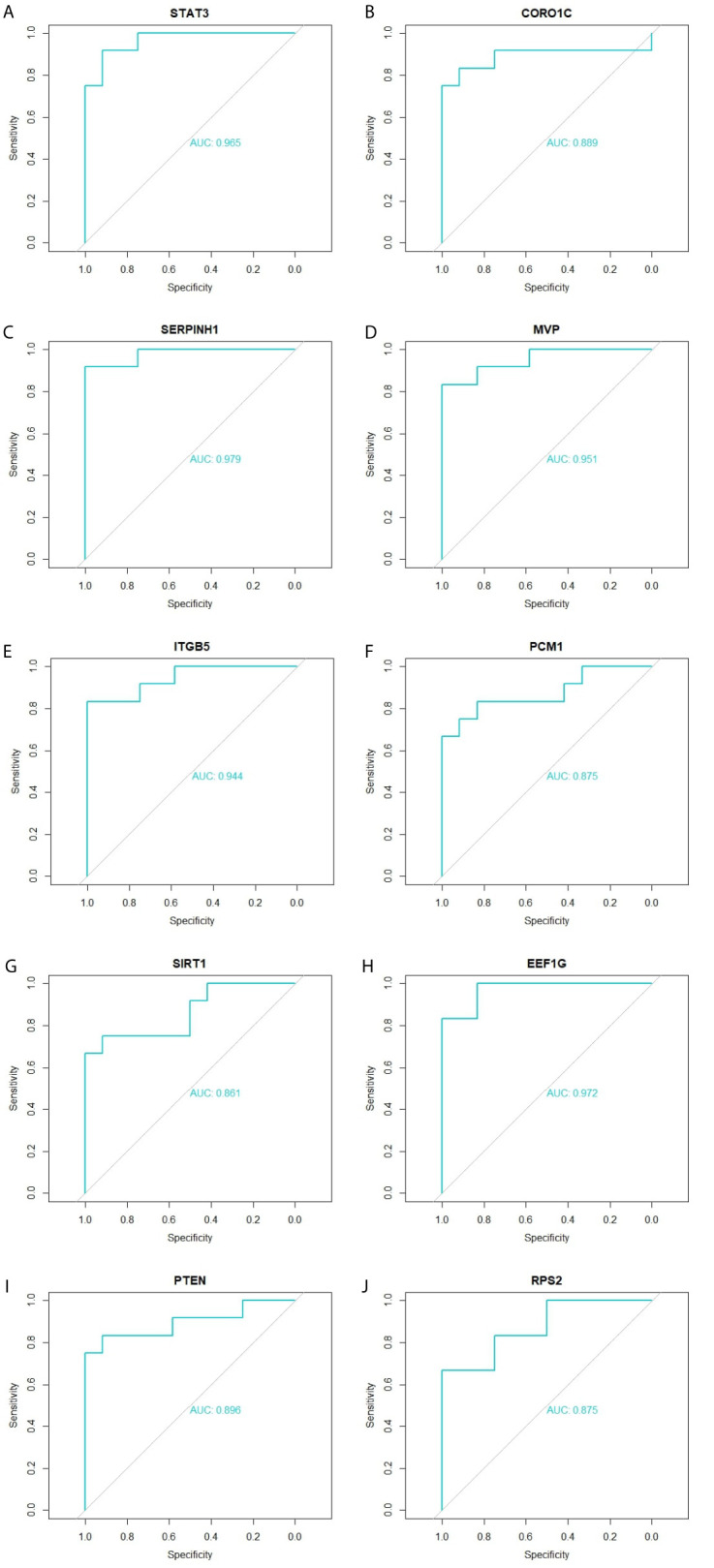
ROC curve validated the sensitivity, specificity of hub genes as a predictive biomarker for obesity prognosis. **(A)** STAT3 **(B)** CORO1C **(C)** SERPINH1 **(D)** MVP **(E)** ITGB5 **(F)** PCM1 **(G)** SIRT1 **(H)** EEF1G **(I)** PTEN **(J)** RPS2.

Immunohistochemical analysis demonstrated that the expression of STAT3, CORO1C, SERPINH1, MVP and ITGB5 were highly expressed in adipose tissues, whereas PCM1, SIRT1, EEF1G, PTEN and RPS2 were low expressed in adipose tissue (**Figure 11I**) and Box plots is showed in **Figure 11II**. Validated by ROC curves, we found that 10 hub genes had high sensitivity and specificity, including STAT3 (0.951), CORO1C (0.799), SERPINH1 (0.924), MVP (0.938), ITGB5 (0.938), PCM1 (0.826), SIRT1 (0.799), EEF1G (0.913), PTEN (0.833) and RPS2 (0.840) (**Figure 12**). The 10 hub genes might be biomarkers of obesity and have positive implications for early medical intervention of the disease.

### Detection of the mRNA Expression of the Hub Genes by RT-PCR

**Figure 13:**
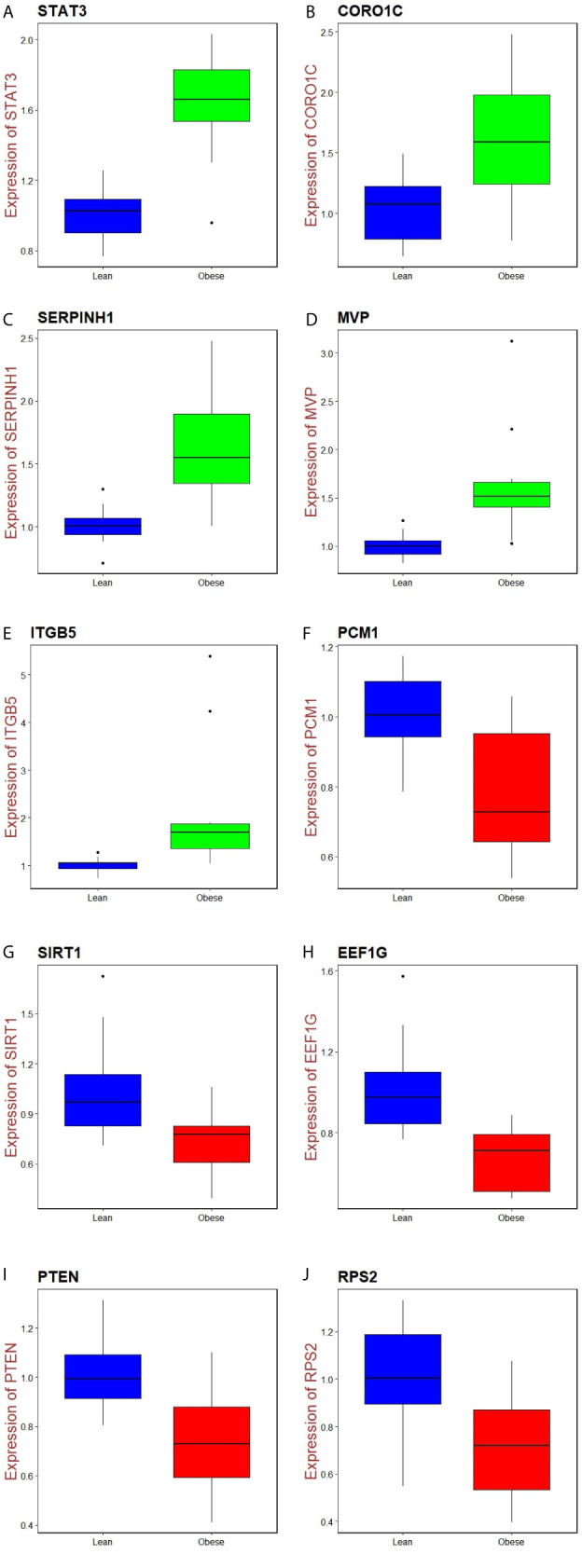
Validation of hub genes by RT- PCR. **(A)** STAT3 **(B)** CORO1C **(C)** SERPINH1 **(D)** MVP E) ITGB5 **(F)** PCM1 **(G)** SIRT1 **(H)** EEF1G **(I)** PTEN **(J)** RPS2.

The adipocytes were removed to detect the mRNA expression levels of hub genes in the PPI network, including STAT3, CORO1C, SERPINH1, MVP, ITGB5, PCM1, SIRT1, EEF1G, PTEN and RPS2. It was found that the mRNA expression levels of STAT3, CORO1C, SERPINH1, MVP and ITGB5 were significantly increased in the obesity compared with the control group. Furthermore, the results illustrate that the mRNA expression levels of PCM1, SIRT1, EEF1G, PTEN and RPS2 were significantly decreased in the obesity compared with the control group (**Figure 13**). Therefore, the RT-PCR results of the hub genes were consistent with the bioinformatics analysis.

### Molecular Docking Studies

**Figure 14:**
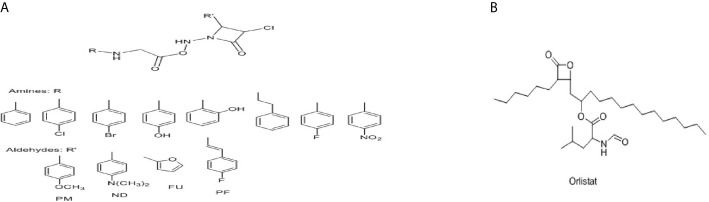
**(A)** Scheme of designed molecule **(B)** Structure of orlistat.

**Figure 15:**
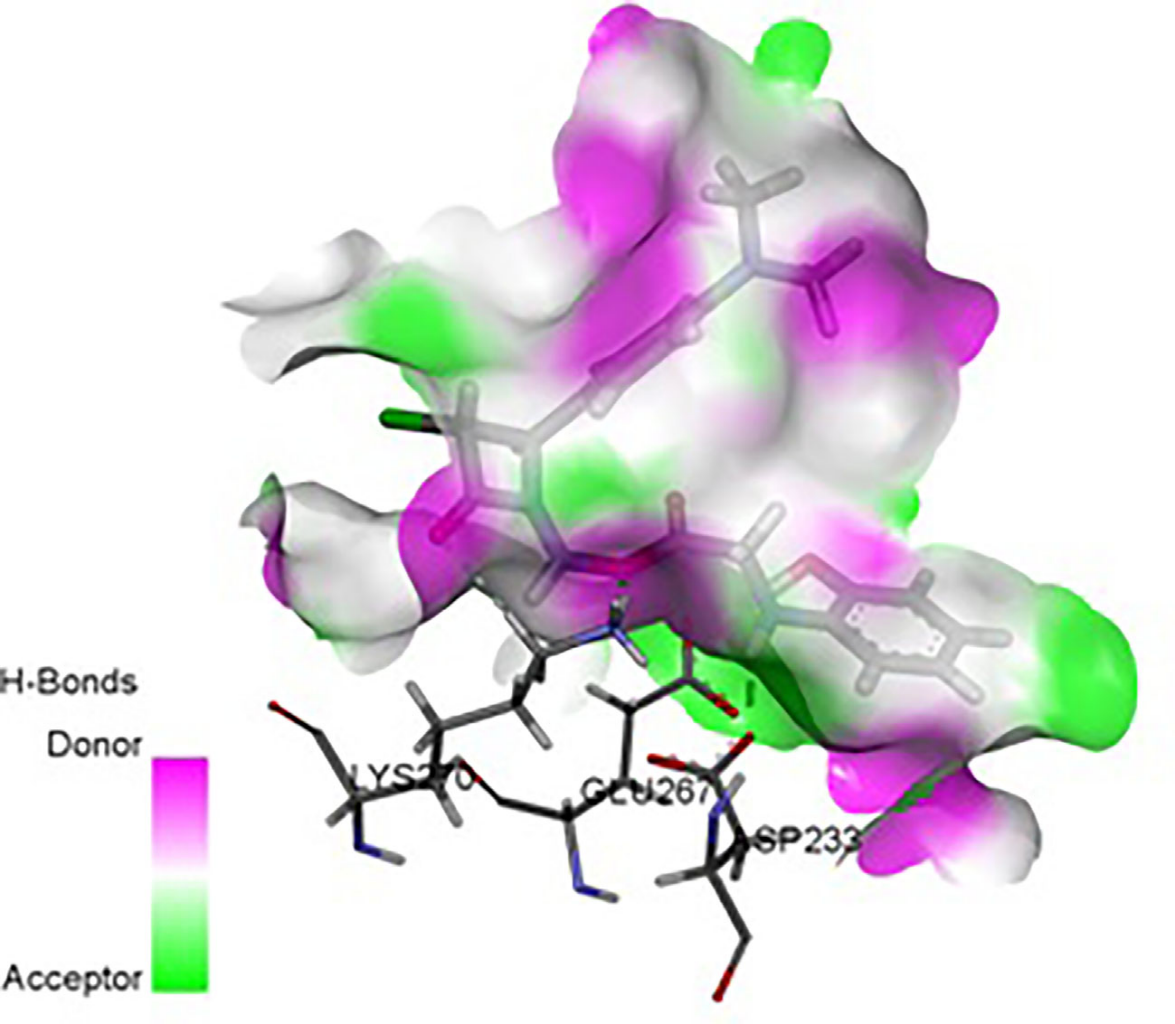
3D Interaction of ND5 with 5OOW.

**Figure 16:**
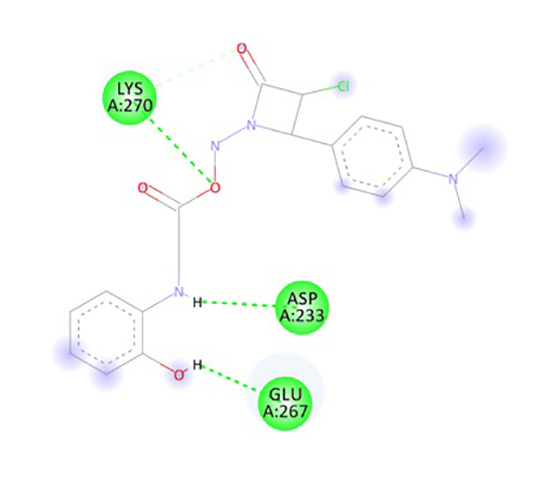
2D Interaction of ND5 with 5OOW.

In the present research, the docking simulations are performed to identify the active site conformation and major interactions responsible for complex stability with the ligand receptor. Designed novel molecules containing four membered more sensitive β-lactam ring, the four membered and performed docking studies using Sybyl X 2.1 drug design software. Molecules containing β-lactam ring is designed which is easily reacting group **Figure 14A**, based on the structure of anti-obesity drug orlistatfour membered ring **Figure 14B**, has potent pancreatic lipase inhibitory activity. The molecules were designed based on the structure of the standard anti-obesity drug orlistat. The one protein in each of three over expressed genes of ERBB2, its co-crystallized protein of PDB code 1MFL,HSPAB 8its co-crystallized protein of PDB code 5OOW and STAT 3its co-crystallized protein of PDB code of 3CWG respectively selected for docking studies. The investigation of designed molecules was performed to identify the potential molecule. The most of the designed molecules with respect to the standard anti-obesity drug orlistat, obtained C-score greater than 5. The C-score greater than 5 are said to be an active, among total of 32 designed molecules few molecules have excellent good binding energy (C-score) greater than 7 respectively. The molecule ND4, FU5 and PF5 obtained score of 7.242, 7.659 and 7.842 with 1MFL and the molecules PM6, ND1, ND3, ND5, ND6, PF5 and PF6 obtained score of 7.5269, 7.6271, 8.0824, 7.6595, 7.0792 and 7.2659 with 3 CWG and the molecules PM4, PM6, ND1, ND5, ND6, PF4, and PF obtained good binding score of 7.1631, 8.8312, 7.3781, 7.9872, 7.9567, 7.0213 and 7.0386 with 5OOW respectively. The molecules found binding score 5-6 is PM1, PM2, PM3, PM4, PM5, PM6, PM7, PM8, ND1, ND2, ND3, ND5, ND6, ND7, ND8, FU1, FU2, FU3, FU4, FU7, FU8, PF1, PF2, PF3, PF4, PF6, PF7, PF8 and standard olistat (STD) with 1MFL and PM2, PM6, FU17, FU18, FU19, FU20, FU23, PF26, PF27, PF28 and PF32 with 3CWG, and PM1, PM2, PM3, PM5, PM7, PM8, ND2, ND3, ND4, ND7, ND8, FU1, FU2, FU3, FU4, FU5, FU6, FU7, FU8, PF1, PF2, PF3, PF6, PF7 and PF8 5OOW respectively. No molecules obtained binding score with less than 5 respectively; the values are depicted in **Supplementary Table 10**. The molecule PF5 has good binding score with all three proteins and ND1, ND3, ND5 and ND6 obtained good binding score with 3CWG and 5OOW. The molecule ND5 has highest binding score and is very close with standard olistat, the interaction with protein 5OOW and hydrogen bonding and other bonding interactions with amino acids are depicted by 3D (**Figure 15**) and 2D (**Figure 16**) images.

## Discussion

Due to the heterogeneity of obesity, obesity was still a disease with high rates of prevalence. This might be due to the scarcity of valid biomarkers for detection of obesity and of valid treatment for obesity. Therefore, molecular mechanisms of obesity are necessary for scientists to find the treat and diagnosis method of obesity. Because of the fast advancement of bioinformatics analysis, it is more convenient to find out the genetic modification in obesity. Bioinformatics analysis enables us to explore the gene, the genetic change in obesity, which had been proved to be a better approach to identify novel biomarkers.

In our study, a total of 876 DEGs were diagnosed from gene expression dataset, consisting of 438 up regulated genes and 438 down regulated genes in obese patients compared to lean persons. Study showed that PTGDS (prostaglandin D2 synthase) (45), LBP (lipopolysaccharide binding protein) (46), EGFL6 (47), STAT3 (48) and HDAC9 (49) were closely associated with obesity. The expression level of CYP11A1 (50) and WNT11 (51) were linked to cancer progression, but these genes might be novel target for obesity. A previous study showed that expression of GPR146 played an important role in insulin resistance (52), but these genes might be novel target for obesity. Aberrant expression of RFX1 (53) and (54) are noticeable factors in the heart disease, but these genes might be novel target for obesity. CLDND1 expression predicted poor therapeutic outcomes of hypertension patients (55).

Functional enrichment analysis of DEGs was implemented. SULT1A1 (56), SULT1A2 (56), COL6A1 (57), COL6A2 (58), SOS1 (59), STAT1 (60), COL5A2 (61), RND3 (62), COL15A1 (63), CBS (cystathionine-beta-synthase) (64), MCM6 (65), TNFRSF12A (66), FMOD (fibromodulin) (67), TYMP (thymidine phosphorylase) (68), ALPL (alkaline phosphatase, biomineralization associated) (69), EFEMP1 (70), MFAP4 (71), IGFBP5 (72), GLUL (glutamate-ammonia ligase) (73), HACD1 (74) and SCP2 (75) have been reported to be biomarkers of heart disease or play a vital role in its pathogenesis, but these genes might be novel target for obesity. Several studies have shown that expressions of COL1A2 (76), COL3A1 (77), EEF2K (78), ANGPT1 (79), NOTCH3 (80) and TGFBR2 (81) can be a strong prognosis biomarker in patients with hypertension, but these genes might be novel target for obesity. DAG1 (82), ITGAV (integrin subunit alpha V) (83), LAMA5 (84), SPP1 (85), COL11A1 (86), COL12A1 (87), SERPINH1 (88), RHOC (89), RPL14 (90), RPL29 (91), RPS12 (92), RPS15A (93), RPS2 (94), RPS27 (95), RPS3 (96), RBL2 (97), EEF1D (98), ACACA (acetyl-CoA carboxylase alpha) (99), ORC2 (100), PHGDH (phosphoglycerate dehydrogenase) (101), SHMT1 (102), NRCAM (neuronal cell adhesion molecule) (103), NRP2 (104), RSPO3 (105), SRPX2 (106), THY1 (107), CD248 (108), CLEC3B (109), CST3 (110), CTHRC1 (111), GPC1 (112), ACSS2 (113) and HSD17B12 (114) have been extensively reported as a tumor biomarkers, but these genes might be novel target for obesity. The results obtained were consistent with studies that role of LAMB3 (115), THBS1 (116), TIMP1 (117), LOX (lysyl oxidase) (118), MMP9 (119), HSD11B1 (120), ITGB2 (121), HMOX1 (122), SOD2 (123), AKR1C3 (124), MAT2B (125), FOXO1 (126), FOXO3 (127), SIRT1 (128), ACACB (acetyl-CoA carboxylase beta) (129), ELK1 (130), MAP3K5 (131), CTH (cystathionine gamma-lyase) (132), AMOT (angiomotin) (133), CCDC80 (134), CXCL10 (135), ERBB2 (136), KLF4 (137), LEP (leptin) (138), MFGE8 (139), SLIT2 (140), TNMD (tenomodulin) (141), ADAMTS5 (142), ELN (elastin) (143), HTRA1 (144), LUM (lumican) (145), MFAP5 (146), IL1RN (147), ACADL (acyl-CoA dehydrogenase long chain) (148), AGT (angiotensinogen) (149), FADS1 (150), PDK4 (151), PER2 (152) and SLC27A2 (153) in obesity. CDKN1B was shown to be a potential predictor of advanced hyperinsulinemia (154), but this gene might be novel target for obesity. Reports illustrate that CXCL12 (155) and IGFBP6 (156) and ELOVL6 (157) were expressed in patients with insulin resistance, but these genes might be novel target for obesity.

Furthermore, by constructing PPI networks and moduleas, we identified some key genes that provide new insights for obesity diagnosis, prognosis, and drug target identification. Expression of the HSPA8 (158) and CKB (159) were correlated with disease grades of hypertension, but these genes might be novel target for obesity. Recent studies have proposed that HSPA5 (160), YWHAH (161), ESR1 (162), PTEN (163), IRAK1 (164), CYR61 (165) and ZBTB16 (166) are involved in obesity. Previous reports demonstrate that SPTAN1 (167), STEAP2 (168), NEK6 (169), ARRB1 (170), FBXO11 (171), UBR2 (172), INTS6 (173), CDK14 (174). LMO2 (175), MSN (176), TAGLN2 (177), SRSF3 (178), SAFB (179), SIN3A (180), TRIM24 (181) and AUTS2 (182) appears to be constitutively activated in cancer, but these genes might be novel target for obesity. CSNK2A2 expression might be regarded as an indicator of susceptibility to heart disease (183), but this gene might be novel target for obesity. COPG2, FBL, CSNK2B, PCM1, ZNF581, KHDRBS1, RBMX, RBBP4 and DCAF7 are novel biomarkers for obesity.

Target genes - miRNA regulatory network and target genes - TF regulatory network were constructed and analyzed. A previous study reported that CCND1 (184) and HP (185) were expressed in obesity. CCND2 (186) and TXNIP (187) are a potential marker for the detection and prognosis of insulin resistance, but these genes might be novel target for obesity. Other research has revealed that BTG2 was expressed in obesity (188). Expression of MED28 (189) and EFNA1 (190) might participate in cancer progression, but these genes might be novel target for obesity. TUBB2A, TMEM189, CNBP, LYZ, TRAM2, MED16, RWDD2A, ADD3 and AIP are a novel biomarkers for obesity.

However, this investigation had some limitations. Primarily, the mechanisms of several hub genes in the pathological process of obesity remain unclear, warranting needs further investigation. Moreover, the success of our small molecule drug compound screening in reducing obesity remains to be assessed.

In conclusion, in this study, we determined that STAT3, CORO1C, SERPINH1, MVP, ITGB5, PCM1, SIRT1, EEF1G, PTEN and RPS2 might be critical genes in the development and prognosis of obesity through bioinformatics analysis combined with validations. However, it is essential that further experiments are carried out and clinical data made available to confirm the results of our investigation and guide the discovery of future gene therapies against obesity.

## Data Availability Statement

The datasets supporting the conclusions of this article are available in the ArrayExpress (https://www.ebi.ac.uk/arrayexpress/) repository. [E-MTAB-6728) (https://www.ebi.ac.uk/arrayexpress/experiments/E-MTAB-6728/)].

## Author Contributions

HJ: Methodology and validation. BV: Writing original draft, and review and editing. NJ: Software and resources. CV: Investigation and resources. AT: Formal analysis and validation. IK: Supervision and resources. All authors contributed to the article and approved the submitted version.

## Conflict of Interest

The authors declare that the research was conducted in the absence of any commercial or financial relationships that could be construed as a potential conflict of interest.
