# Supplementary material for: Identification of Key Pathways and Genes in Obesity Using Bioinformatics Analysis and Molecular Docking Studies
Source: Front Endocrinol (Lausanne). 2021 Jun 24;12:628907. doi: 10.3389/fendo.2021.628907 (PMC8264660; doi:10.3389/fendo.2021.628907)
Supplement: Supplementary file 1 [file DataSheet_1.docx]

**Table 1**. Primers used for quantitative PCR

| **Primer sequence (5'→3')** | | |
| --- | --- | --- |
| **Gene** | **Forward** | **Reverse** |
| STAT3 | CAGCAGCTTGACACACGGTA | AAACACCAAAGTGGCATGTGA |
| CORO1C | ATGAGGCGAGTGGTACGACA | ATCCCAGGTCACACGAGAAAC |
| SERPINH1 | TCAGTGAGCTTCGCTGATGAC | CATGGCGTTGACTAGCAGGG |
| MVP | TACATCCGGCAGGACAATGAG | CTGTGCAGTAGTGACGTGGG |
| ITGB5 | TCTCGGTGTGATCTGAGGG | TGGCGAACCTGTAGCTGGA |
| PCM1 | CGGAGTCGTCACCAGGAGT | GCTGTTGCTCTACCTTGGGAAT |
| SIRT1 | TAGCCTTGTCAGATAAGGAAGGA | ACAGCTTCACAGTCAACTTTGT |
| EEF1G | AACCGCACCCCTGAATTTCTC | GGCGTTGCTCTCAAACACAC |
| PTEN | TGGATTCGACTTAGACTTGACCT | GGTGGGTTATGGTCTTCAAAAGG |
| RPS2 | GGCCTCTCTCAAGGATGAGGT | GTCCCCGATAGCAACAAATGC |

**Table 2** The statistical metrics for key differentially expressed genes (DEGs)

| **Illumina Id** | **Gene Symbol** | **logFC** | **p Value** | **Adj P value** | **t value** | **Regulation** | **Gene Name** |
| --- | --- | --- | --- | --- | --- | --- | --- |
| ILMN_1664464 | PTGDS | 1.820427 | 5.99E-10 | 5.99E-10 | 9.552145 | Up | prostaglandin D2 synthase |
| ILMN_1732538 | LBP | 2.225478 | 6.05E-10 | 6.05E-10 | 9.547636 | Up | lipopolysaccharide binding protein |
| ILMN_1768820 | CYP11A1 | 0.714722 | 8.98E-10 | 8.98E-10 | 9.362374 | Up | cytochrome P450 family 11 subfamily A member 1 |
| ILMN_1716309 | EGFL6 | 3.153351 | 2.55E-09 | 2.55E-09 | 8.884119 | Up | EGF like domain multiple 6 |
| ILMN_2401978 | STAT3 | 0.708174 | 8.88E-09 | 8.88E-09 | 8.328804 | Up | signal transducer and activator of transcription 3 |
| ILMN_1785852 | NABP1 | 1.234646 | 9.01E-09 | 9.01E-09 | 8.322415 | Up | nucleic acid binding protein 1 |
| ILMN_1709750 | SUSD1 | 0.818338 | 1.47E-08 | 1.47E-08 | 8.108538 | Up | sushi domain containing 1 |
| ILMN_1800512 | HMOX1 | 1.385702 | 3.42E-08 | 3.42E-08 | 7.747992 | Up | hemeoxygenase 1 |
| ILMN_2057479 | EGFL6 | 3.924423 | 3.64E-08 | 3.64E-08 | 7.721939 | Up | EGF like domain multiple 6 |
| ILMN_2141118 | INSYN1 | 0.859189 | 5.67E-08 | 5.67E-08 | 7.535199 | Up | inhibitory synaptic factor 1 |
| ILMN_1715662 | CCDC80 | 0.999509 | 8.82E-08 | 8.82E-08 | 7.351189 | Up | coiled-coil domain containing 80 |
| ILMN_2109708 | TYMP | 1.207265 | 1.12E-07 | 1.12E-07 | 7.250807 | Up | thymidine phosphorylase |
| ILMN_2400372 | SULT1A2 | 1.110385 | 1.61E-07 | 1.61E-07 | 7.102387 | Up | sulfotransferase family 1A member 2 |
| ILMN_1755792 | STK38L | 0.554845 | 1.67E-07 | 1.67E-07 | 7.089178 | Up | serine/threonine kinase 38 like |
| ILMN_1663618 | STAT3 | 0.567385 | 1.71E-07 | 1.71E-07 | 7.07883 | Up | signal transducer and activator of transcription 3 |
| ILMN_1798528 | SULT1A2 | 1.001497 | 2.89E-07 | 2.89E-07 | 6.86514 | Up | sulfotransferase family 1A member 2 |
| ILMN_1660021 | PLIN3 | 0.659133 | 8.1E-07 | 8.1E-07 | 6.452309 | Up | perilipin 3 |
| ILMN_1910180 | NRP2 | 1.230106 | 8.62E-07 | 8.62E-07 | 6.427843 | Up | neuropilin 2 |
| ILMN_1741404 | MSC | 1.576437 | 9.09E-07 | 9.09E-07 | 6.406619 | Up | musculin |
| ILMN_1676067 | SEMA3G | 1.155506 | 9.21E-07 | 9.21E-07 | 6.401565 | Up | semaphorin 3G |
| ILMN_1722809 | NRCAM | 1.386089 | 1.03E-06 | 1.03E-06 | 6.356808 | Up | neuronal cell adhesion molecule |
| ILMN_1652631 | GLIPR2 | 1.728391 | 1.1E-06 | 1.1E-06 | 6.332413 | Up | GLI pathogenesis related 2 |
| ILMN_1690939 | TYMP | 0.635845 | 1.11E-06 | 1.11E-06 | 6.327574 | Up | thymidine phosphorylase |
| ILMN_1750674 | SDSL | 0.768954 | 1.11E-06 | 1.11E-06 | 6.325979 | Up | serine dehydratase like |
| ILMN_1687216 | PCBP3 | 0.721533 | 1.56E-06 | 1.56E-06 | 6.192409 | Up | poly(rC) binding protein 3 |
| ILMN_2104295 | TMEM178A | 0.848363 | 1.6E-06 | 1.6E-06 | 6.184294 | Up | transmembrane protein 178A |
| ILMN_1726589 | CD248 | 1.778343 | 1.77E-06 | 1.77E-06 | 6.143392 | Up | CD248 molecule |
| ILMN_2169439 | ITGAV | 0.911775 | 2.01E-06 | 2.01E-06 | 6.094822 | Up | integrin subunit alpha V |
| ILMN_1658926 | NOTCH3 | 1.789129 | 2.13E-06 | 2.13E-06 | 6.07107 | Up | notch 3 |
| ILMN_1698732 | PALLD | 1.949881 | 2.4E-06 | 2.4E-06 | 6.025117 | Up | palladin, cytoskeletal associated protein |
| ILMN_1715684 | LAMB3 | 1.217439 | 2.54E-06 | 2.54E-06 | 6.002744 | Up | laminin subunit beta 3 |
| ILMN_1675656 | PPFIBP2 | 0.586918 | 2.79E-06 | 2.79E-06 | 5.966758 | Up | PPFIA binding protein 2 |
| ILMN_1751028 | SERPINH1 | 0.710927 | 2.9E-06 | 2.9E-06 | 5.951672 | Up | serpin family H member 1 |
| ILMN_1701603 | ALPL | 1.630113 | 2.95E-06 | 2.95E-06 | 5.943838 | Up | alkaline phosphatase, biomineralization associated |
| ILMN_1775542 | FCMR | 0.672225 | 3.36E-06 | 3.36E-06 | 5.893893 | Up | Fc fragment of IgM receptor |
| ILMN_1720484 | CRTAP | 1.04261 | 3.58E-06 | 3.58E-06 | 5.86966 | Up | cartilage associated protein |
| ILMN_1793695 | ITIH5 | 1.374714 | 4.1E-06 | 4.1E-06 | 5.816395 | Up | inter-alpha-trypsin inhibitor heavy chain family member 5 |
| ILMN_1714586 | VGLL3 | 1.26809 | 4.16E-06 | 4.16E-06 | 5.810654 | Up | vestigial like family member 3 |
| ILMN_1732197 | MN1 | 0.948733 | 4.26E-06 | 4.26E-06 | 5.802156 | Up | MN1 proto-oncogene, transcriptional regulator |
| ILMN_1680874 | TUBB2B | 1.715743 | 4.64E-06 | 4.64E-06 | 5.768826 | Up | tubulin beta 2B class IIb |
| ILMN_1656900 | SULT1A3 | 1.129801 | 6.1E-06 | 6.1E-06 | 5.662817 | Up | sulfotransferase family 1A member 3 |
| ILMN_2344373 | MVP | 0.650428 | 6.34E-06 | 6.34E-06 | 5.647981 | Up | major vault protein |
| ILMN_1676449 | SLIT2 | 1.051292 | 7.26E-06 | 7.26E-06 | 5.595914 | Up | slit guidance ligand 2 |
| ILMN_1715416 | NUP188 | 0.674676 | 7.47E-06 | 7.47E-06 | 5.584899 | Up | nucleoporin 188 |
| ILMN_1789507 | COL11A1 | 1.258588 | 7.99E-06 | 7.99E-06 | 5.558827 | Up | collagen type XI alpha 1 chain |
| ILMN_1658425 | DAG1 | 0.528968 | 8.18E-06 | 8.18E-06 | 5.549832 | Up | dystroglycan 1 |
| ILMN_1678403 | TMEM178A | 0.540004 | 8.25E-06 | 8.25E-06 | 5.546888 | Up | transmembrane protein 178A |
| ILMN_1801516 | GPC1 | 0.702812 | 8.47E-06 | 8.47E-06 | 5.536387 | Up | glypican 1 |
| ILMN_1718285 | HOXC8 | 0.916774 | 8.51E-06 | 8.51E-06 | 5.534712 | Up | homeobox C8 |
| ILMN_1734184 | PLBD2 | 0.814142 | 8.63E-06 | 8.63E-06 | 5.52926 | Up | phospholipase B domain containing 2 |
| ILMN_1809928 | COL6A2 | 1.55151 | 8.7E-06 | 8.7E-06 | 5.526418 | Up | collagen type VI alpha 2 chain |
| ILMN_2139970 | ALDH1A3 | 2.346733 | 9.12E-06 | 9.12E-06 | 5.508259 | Up | aldehyde dehydrogenase 1 family member A3 |
| ILMN_1658619 | WWC1 | 0.614241 | 9.14E-06 | 9.14E-06 | 5.507185 | Up | WW and C2 domain containing 1 |
| ILMN_2111237 | MN1 | 0.93355 | 9.31E-06 | 9.31E-06 | 5.5002 | Up | MN1 proto-oncogene, transcriptional regulator |
| ILMN_1853824 | MGAT3 | 0.828545 | 1.13E-05 | 1.13E-05 | 5.424362 | Up | mannosyl (beta-1,4-)-glycoprotein beta-1,4-N-acetylglucosaminyltransferase |
| ILMN_1750101 | S100A11 | 0.735352 | 1.19E-05 | 1.19E-05 | 5.404472 | Up | S100 calcium binding protein A11 |
| ILMN_1803338 | CCDC80 | 0.979808 | 1.2E-05 | 1.2E-05 | 5.403253 | Up | coiled-coil domain containing 80 |
| ILMN_1701613 | RARRES3 | 1.103884 | 1.25E-05 | 1.25E-05 | 5.387103 | Up | retinoic acid receptor responder 3 |
| ILMN_1803236 | CLCA2 | 0.574002 | 1.26E-05 | 1.26E-05 | 5.384393 | Up | chloride channel accessory 2 |
| ILMN_1807439 | ALDH1A3 | 1.954717 | 1.31E-05 | 1.31E-05 | 5.369138 | Up | aldehyde dehydrogenase 1 family member A3 |
| ILMN_1759374 | LINC00545 | 1.309512 | 1.39E-05 | 1.39E-05 | 5.345927 | Up | long intergenic non-protein coding RNA 545 |
| ILMN_1803277 | MVP | 0.916886 | 1.4E-05 | 1.4E-05 | 5.344141 | Up | major vault protein |
| ILMN_1771179 | CYB561 | 0.539323 | 1.46E-05 | 1.46E-05 | 5.327812 | Up | cytochrome b561 |
| ILMN_1887987 | LINC00968 | 0.855428 | 1.54E-05 | 1.54E-05 | 5.307616 | Up | long intergenic non-protein coding RNA 968 |
| ILMN_1689968 | PLEKHO2 | 0.895733 | 1.58E-05 | 1.58E-05 | 5.29842 | Up | pleckstrin homology domain containing O2 |
| ILMN_1763587 | TNMD | 2.019209 | 1.69E-05 | 1.69E-05 | 5.272155 | Up | tenomodulin |
| ILMN_1814282 | AEN | 0.629178 | 1.81E-05 | 1.81E-05 | 5.245312 | Up | apoptosis enhancing nuclease |
| ILMN_2336133 | SULT1A4 | 0.781945 | 1.82E-05 | 1.82E-05 | 5.24246 | Up | sulfotransferase family 1A member 4 |
| ILMN_1783909 | COL6A2 | 1.730795 | 1.94E-05 | 1.94E-05 | 5.218859 | Up | collagen type VI alpha 2 chain |
| ILMN_1691884 | STC2 | 1.156543 | 2.19E-05 | 2.19E-05 | 5.171969 | Up | stanniocalcin 2 |
| ILMN_1732151 | COL6A1 | 1.298231 | 2.36E-05 | 2.36E-05 | 5.143952 | Up | collagen type VI alpha 1 chain |
| ILMN_1752299 | RAB6B | 0.714693 | 2.59E-05 | 2.59E-05 | 5.109432 | Up | RAB6B, member RAS oncogene family |
| ILMN_1677814 | ABCC3 | 1.564486 | 2.67E-05 | 2.67E-05 | 5.097746 | Up | ATP binding cassette subfamily C member 3 |
| ILMN_1779147 | ENC1 | 0.85892 | 2.74E-05 | 2.74E-05 | 5.087435 | Up | ectodermal-neural cortex 1 |
| ILMN_1750409 | RAB9A | 0.555529 | 2.78E-05 | 2.78E-05 | 5.081386 | Up | RAB9A, member RAS oncogene family |
| ILMN_1666594 | IRF8 | 1.300268 | 2.88E-05 | 2.88E-05 | 5.068737 | Up | interferon regulatory factor 8 |
| ILMN_1796316 | MMP9 | 1.65811 | 3.26E-05 | 3.26E-05 | 5.020792 | Up | matrix metallopeptidase 9 |
| ILMN_1813139 | ANKDD1A | 1.12868 | 3.44E-05 | 3.44E-05 | 5.000054 | Up | ankyrin repeat and death domain containing 1A |
| ILMN_2306661 | UNC13C | 0.623986 | 3.56E-05 | 3.56E-05 | 4.987296 | Up | unc-13 homolog C |
| ILMN_2188722 | GLS | 0.666142 | 3.56E-05 | 3.56E-05 | 4.987083 | Up | glutaminase |
| ILMN_2062620 | NMT2 | 0.699357 | 3.57E-05 | 3.57E-05 | 4.986309 | Up | N-myristoyltransferase 2 |
| ILMN_1771688 | RAB7B | 0.985922 | 3.77E-05 | 3.77E-05 | 4.966148 | Up | RAB7B, member RAS oncogene family |
| ILMN_1749868 | FAM171A1 | 0.805476 | 3.84E-05 | 3.84E-05 | 4.95859 | Up | family with sequence similarity 171 member A1 |
| ILMN_1801226 | DOCK6 | 0.88875 | 3.94E-05 | 3.94E-05 | 4.949344 | Up | dedicator of cytokinesis 6 |
| ILMN_1657129 | SKAP2 | 0.56751 | 3.95E-05 | 3.95E-05 | 4.947644 | Up | src kinase associated phosphoprotein 2 |
| ILMN_1678757 | BCYRN1 | 1.528559 | 4.21E-05 | 4.21E-05 | 4.923778 | Up | brain cytoplasmic RNA 1 |
| ILMN_1773079 | COL3A1 | 1.465245 | 4.58E-05 | 4.58E-05 | 4.891919 | Up | collagen type III alpha 1 chain |
| ILMN_1729596 | INF2 | 0.717482 | 4.84E-05 | 4.84E-05 | 4.870531 | Up | inverted formin, FH2 and WH2 domain containing |
| ILMN_1779416 | SCUBE2 | 0.812412 | 4.93E-05 | 4.93E-05 | 4.86363 | Up | signal peptide, CUB domain and EGF like domain containing 2 |
| ILMN_2397721 | GLB1 | 0.668144 | 5.28E-05 | 5.28E-05 | 4.837888 | Up | galactosidase beta 1 |
| ILMN_1691364 | STAT1 | 0.649399 | 5.38E-05 | 5.38E-05 | 4.830063 | Up | signal transducer and activator of transcription 1 |
| ILMN_1735155 | GLB1 | 0.738614 | 5.47E-05 | 5.47E-05 | 4.824387 | Up | galactosidase beta 1 |
| ILMN_1800354 | CST3 | 0.562598 | 5.91E-05 | 5.91E-05 | 4.794968 | Up | cystatin C |
| ILMN_1804007 | NANOS3 | 0.541781 | 6.12E-05 | 6.12E-05 | 4.781403 | Up | nanos C2HC-type zinc finger 3 |
| ILMN_2335557 | ITIH5 | 0.80499 | 6.15E-05 | 6.15E-05 | 4.779724 | Up | inter-alpha-trypsin inhibitor heavy chain family member 5 |
| ILMN_1686367 | HSPA8 | 0.557034 | 6.18E-05 | 6.18E-05 | 4.777626 | Up | heat shock protein family A (Hsp70) member 8 |
| ILMN_2313730 | RHOC | 0.613535 | 6.46E-05 | 6.46E-05 | 4.760619 | Up | ras homolog family member C |
| ILMN_2379130 | IRAK1 | 0.562669 | 6.98E-05 | 6.98E-05 | 4.731406 | Up | interleukin 1 receptor associated kinase 1 |
| ILMN_1660871 | NEK6 | 0.613802 | 7.12E-05 | 7.12E-05 | 4.723617 | Up | NIMA related kinase 6 |
| ILMN_1690105 | STAT1 | 0.563232 | 7.21E-05 | 7.21E-05 | 4.718878 | Up | signal transducer and activator of transcription 1 |
| ILMN_1667295 | VASN | 0.99406 | 7.24E-05 | 7.24E-05 | 4.717494 | Up | vasorin |
| ILMN_1754842 | DLGAP4 | 0.561107 | 7.94E-05 | 7.94E-05 | 4.682126 | Up | DLG associated protein 4 |
| ILMN_1789733 | CLIP3 | 0.953388 | 7.98E-05 | 7.98E-05 | 4.680665 | Up | CAP-Gly domain containing linker protein 3 |
| ILMN_1720430 | PPDPF | 0.580876 | 8.33E-05 | 8.33E-05 | 4.664348 | Up | pancreatic progenitor cell differentiation and proliferation factor |
| ILMN_2378376 | CYB561 | 0.526267 | 8.52E-05 | 8.52E-05 | 4.655456 | Up | cytochrome b561 |
| ILMN_2411236 | NRCAM | 1.443196 | 8.72E-05 | 8.72E-05 | 4.646872 | Up | neuronal cell adhesion molecule |
| ILMN_1805992 | SHTN1 | 0.731593 | 9.13E-05 | 9.13E-05 | 4.629195 | Up | shootin 1 |
| ILMN_1735124 | OXT | 0.578334 | 9.16E-05 | 9.16E-05 | 4.627921 | Up | oxytocin/neurophysin I prepropeptide |
| ILMN_1693826 | HAVCR2 | 0.722164 | 9.19E-05 | 9.19E-05 | 4.626647 | Up | hepatitis A virus cellular receptor 2 |
| ILMN_1791569 | PLXNA1 | 0.62569 | 9.48E-05 | 9.48E-05 | 4.615066 | Up | plexin A1 |
| ILMN_2224486 | C3orf14 | 0.788002 | 0.000102 | 0.000102 | 4.588076 | Up | chromosome 3 open reading frame 14 |
| ILMN_2038775 | TUBB2A | 1.288125 | 0.00011 | 0.00011 | 4.55718 | Up | tubulin beta 2A class IIa |
| ILMN_1763640 | NCKAP5L | 0.532109 | 0.000113 | 0.000113 | 4.548191 | Up | NCK associated protein 5 like |
| ILMN_1731862 | ITIH5 | 0.857364 | 0.000119 | 0.000119 | 4.527192 | Up | inter-alpha-trypsin inhibitor heavy chain family member 5 |
| ILMN_1796734 | SPARC | 0.8005 | 0.000127 | 0.000127 | 4.504283 | Up | secreted protein acidic and cysteine rich |
| ILMN_1707804 | SLX1A-SULT1A3 | 0.634745 | 0.000127 | 0.000127 | 4.503092 | Up | SLX1A-SULT1A3 readthrough (NMD candidate) |
| ILMN_2374352 | DBNDD1 | 0.617377 | 0.000129 | 0.000129 | 4.498243 | Up | dysbindin domain containing 1 |
| ILMN_2121816 | GPR137B | 0.679236 | 0.00013 | 0.00013 | 4.495797 | Up | G protein-coupled receptor 137B |
| ILMN_1908923 | GRIN2B | 1.478932 | 0.000132 | 0.000132 | 4.488805 | Up | glutamate ionotropic receptor NMDA type subunit 2B |
| ILMN_1745806 | PEMT | 1.18577 | 0.000136 | 0.000136 | 4.478651 | Up | phosphatidylethanolamine N-methyltransferase |
| ILMN_1782412 | IRX2 | 0.640361 | 0.000138 | 0.000138 | 4.471406 | Up | iroquoishomeobox 2 |
| ILMN_2171295 | CDK14 | 0.526289 | 0.000139 | 0.000139 | 4.47008 | Up | cyclin dependent kinase 14 |
| ILMN_1794190 | CCPG1 | 0.595489 | 0.000142 | 0.000142 | 4.461295 | Up | cell cycle progression 1 |
| ILMN_1757387 | UCHL1 | 1.625354 | 0.000147 | 0.000147 | 4.44834 | Up | ubiquitin C-terminal hydrolase L1 |
| ILMN_1744487 | MFRP | 1.085469 | 0.000153 | 0.000153 | 4.432535 | Up | membrane frizzled-related protein |
| ILMN_1683146 | FTH1 | 0.709387 | 0.000153 | 0.000153 | 4.431824 | Up | ferritin heavy chain 1 |
| ILMN_1728512 | YWHAH | 0.734671 | 0.000154 | 0.000154 | 4.429773 | Up | tyrosine 3-monooxygenase/tryptophan 5-monooxygenase activation protein eta |
| ILMN_2352131 | ERBB2 | 0.825698 | 0.000161 | 0.000161 | 4.412124 | Up | erb-b2 receptor tyrosine kinase 2 |
| ILMN_1656378 | NMT2 | 0.637872 | 0.000163 | 0.000163 | 4.409175 | Up | N-myristoyltransferase 2 |
| ILMN_1788783 | TRAM2 | 0.731691 | 0.000169 | 0.000169 | 4.395858 | Up | translocation associated membrane protein 2 |
| ILMN_1812461 | WISP2 | 1.648741 | 0.000172 | 0.000172 | 4.389102 | Up | WNT1 inducible signaling pathway protein 2 |
| ILMN_1796755 | ITGB5 | 0.824151 | 0.000178 | 0.000178 | 4.37583 | Up | integrin subunit beta 5 |
| ILMN_1675062 | MYL9 | 0.704484 | 0.000184 | 0.000184 | 4.361427 | Up | myosin light chain 9 |
| ILMN_1705984 | HNMT | 0.55377 | 0.000195 | 0.000195 | 4.34004 | Up | histamine N-methyltransferase |
| ILMN_1733415 | MFAP5 | 1.982064 | 0.000204 | 0.000204 | 4.323196 | Up | microfibril associated protein 5 |
| ILMN_1721605 | SMYD2 | 0.535837 | 0.00021 | 0.00021 | 4.311879 | Up | SET and MYND domain containing 2 |
| ILMN_2179717 | FAM189A2 | 0.524393 | 0.000212 | 0.000212 | 4.308716 | Up | family with sequence similarity 189 member A2 |
| ILMN_1787127 | SLC43A2 | 0.574814 | 0.000217 | 0.000217 | 4.300207 | Up | solute carrier family 43 member 2 |
| ILMN_1744517 | GNS | 0.548078 | 0.00022 | 0.00022 | 4.293593 | Up | glucosamine (N-acetyl)-6-sulfatase |
| ILMN_1754660 | ZCCHC24 | 0.577849 | 0.000221 | 0.000221 | 4.292656 | Up | zinc finger CCHC-type containing 24 |
| ILMN_1685824 | B4GALT5 | 0.642948 | 0.000222 | 0.000222 | 4.291033 | Up | beta-1,4-galactosyltransferase 5 |
| ILMN_1685714 | INHBB | 0.880027 | 0.000232 | 0.000232 | 4.27473 | Up | inhibin subunit beta B |
| ILMN_1713124 | AKR1C3 | 0.814393 | 0.000233 | 0.000233 | 4.272982 | Up | aldo-ketoreductase family 1 member C3 |
| ILMN_2404795 | SULT1A1 | 0.54791 | 0.000243 | 0.000243 | 4.25676 | Up | sulfotransferase family 1A member 1 |
| ILMN_1668374 | ITGB5 | 0.846685 | 0.000254 | 0.000254 | 4.239363 | Up | integrin subunit beta 5 |
| ILMN_1774229 | SLC7A4 | 0.588643 | 0.000254 | 0.000254 | 4.238984 | Up | solute carrier family 7 member 4 |
| ILMN_1798496 | HOXB8 | 0.831195 | 0.000268 | 0.000268 | 4.218942 | Up | homeobox B8 |
| ILMN_1654262 | ZMAT3 | 0.568561 | 0.000269 | 0.000269 | 4.216909 | Up | zinc finger matrin-type 3 |
| ILMN_1736730 | CARMIL1 | 0.532451 | 0.000283 | 0.000283 | 4.198429 | Up | capping protein regulator and myosin 1 linker 1 |
| ILMN_1658639 | SLC46A3 | 0.534039 | 0.000287 | 0.000287 | 4.192647 | Up | solute carrier family 46 member 3 |
| ILMN_1695880 | LOX | 1.159643 | 0.00029 | 0.00029 | 4.189243 | Up | lysyl oxidase |
| ILMN_2343278 | PLPP1 | 0.762711 | 0.000294 | 0.000294 | 4.182978 | Up | phospholipid phosphatase 1 |
| ILMN_2162989 | TMEM189 | 0.5836 | 0.000297 | 0.000297 | 4.179119 | Up | transmembrane protein 189 |
| ILMN_1812433 | HP | 1.60106 | 0.000303 | 0.000303 | 4.172329 | Up | haptoglobin |
| ILMN_2044813 | TUBB2A | 1.233632 | 0.000309 | 0.000309 | 4.164915 | Up | tubulin beta 2A class IIa |
| ILMN_1692077 | MXRA7 | 0.862596 | 0.000312 | 0.000312 | 4.160596 | Up | matrix remodeling associated 7 |
| ILMN_1759513 | RND3 | 0.776115 | 0.000319 | 0.000319 | 4.151717 | Up | Rho family GTPase 3 |
| ILMN_1701195 | PLA2G7 | 0.874848 | 0.000321 | 0.000321 | 4.150019 | Up | phospholipase A2 group VII |
| ILMN_1809496 | COPG2 | 0.790334 | 0.000324 | 0.000324 | 4.145622 | Up | coatomer protein complex subunit gamma 2 |
| ILMN_2336781 | SOD2 | 0.621923 | 0.000333 | 0.000333 | 4.135996 | Up | superoxide dismutase 2 |
| ILMN_2095133 | SPTAN1 | 0.557462 | 0.000359 | 0.000359 | 4.106471 | Up | spectrin alpha, non-erythrocytic 1 |
| ILMN_1674620 | SGCE | 0.622138 | 0.000374 | 0.000374 | 4.090801 | Up | sarcoglycan epsilon |
| ILMN_1666502 | SOBP | 0.667445 | 0.0004 | 0.0004 | 4.065752 | Up | sine oculis binding protein homolog |
| ILMN_2316386 | GPBAR1 | 0.828862 | 0.000402 | 0.000402 | 4.063058 | Up | G protein-coupled bile acid receptor 1 |
| ILMN_2377900 | MAP1B | 1.140578 | 0.000411 | 0.000411 | 4.055336 | Up | microtubule associated protein 1B |
| ILMN_2232177 | ACTN1 | 0.63592 | 0.000419 | 0.000419 | 4.047935 | Up | actinin alpha 1 |
| ILMN_1795679 | STMN2 | 1.434038 | 0.000434 | 0.000434 | 4.033809 | Up | stathmin 2 |
| ILMN_1771261 | SYNC | 1.004439 | 0.000455 | 0.000455 | 4.015841 | Up | syncoilin, intermediate filament protein |
| ILMN_2413644 | TM4SF19 | 0.839844 | 0.00046 | 0.00046 | 4.011693 | Up | transmembrane 4 L six family member 19 |
| ILMN_1781626 | C1S | 1.250935 | 0.00046 | 0.00046 | 4.011381 | Up | complement C1s |
| ILMN_2115862 | ESPNL | 0.592832 | 0.000461 | 0.000461 | 4.011043 | Up | espin like |
| ILMN_2202915 | FAR2 | 0.717943 | 0.000463 | 0.000463 | 4.008904 | Up | fatty acyl-CoA reductase 2 |
| ILMN_1730416 | CYCS | 0.538013 | 0.000473 | 0.000473 | 4.000795 | Up | cytochrome c, somatic |
| ILMN_1733288 | C1RL | 0.569636 | 0.000479 | 0.000479 | 3.996304 | Up | complement C1r subcomponent like |
| ILMN_2187746 | EMX2 | 0.526956 | 0.000491 | 0.000491 | 3.986382 | Up | empty spiracles homeobox 2 |
| ILMN_1737163 | SH3BGRL3 | 0.572465 | 0.000492 | 0.000492 | 3.985522 | Up | SH3 domain binding glutamate rich protein like 3 |
| ILMN_1670870 | ALCAM | 0.68422 | 0.000506 | 0.000506 | 3.975 | Up | activated leukocyte cell adhesion molecule |
| ILMN_1669362 | IGFBP6 | 1.927527 | 0.000529 | 0.000529 | 3.958008 | Up | insulin like growth factor binding protein 6 |
| ILMN_1773865 | HSPA5 | 0.542294 | 0.000535 | 0.000535 | 3.953083 | Up | heat shock protein family A (Hsp70) member 5 |
| ILMN_1791494 | SYNPO2 | 0.555068 | 0.000563 | 0.000563 | 3.933878 | Up | synaptopodin 2 |
| ILMN_1727709 | GPBAR1 | 0.738312 | 0.000582 | 0.000582 | 3.920655 | Up | G protein-coupled bile acid receptor 1 |
| ILMN_1766914 | MFAP4 | 1.402644 | 0.000585 | 0.000585 | 3.91908 | Up | microfibril associated protein 4 |
| ILMN_2059689 | TMEM54 | 0.718887 | 0.000587 | 0.000587 | 3.917919 | Up | transmembrane protein 54 |
| ILMN_1653161 | SNCG | 1.257281 | 0.000602 | 0.000602 | 3.908096 | Up | synuclein gamma |
| ILMN_2370208 | CMTM3 | 0.671258 | 0.000625 | 0.000625 | 3.893215 | Up | CKLF like MARVEL transmembrane domain containing 3 |
| ILMN_1712545 | S100A3 | 0.548387 | 0.000634 | 0.000634 | 3.888169 | Up | S100 calcium binding protein A3 |
| ILMN_1719316 | TMED3 | 1.118639 | 0.000674 | 0.000674 | 3.864212 | Up | transmembrane p24 trafficking protein 3 |
| ILMN_1789196 | TPM2 | 0.999608 | 0.000674 | 0.000674 | 3.864171 | Up | tropomyosin 2 |
| ILMN_1676663 | TNFRSF11B | 0.737053 | 0.000674 | 0.000674 | 3.863984 | Up | TNF receptor superfamily member 11b |
| ILMN_1752802 | CLPTM1L | 0.538557 | 0.000681 | 0.000681 | 3.860129 | Up | CLPTM1 like |
| ILMN_1776953 | MYL9 | 0.73886 | 0.000694 | 0.000694 | 3.853141 | Up | myosin light chain 9 |
| ILMN_1688295 | ZNF219 | 0.693667 | 0.000695 | 0.000695 | 3.852426 | Up | zinc finger protein 219 |
| ILMN_1653006 | CSN1S1 | 1.490032 | 0.000707 | 0.000707 | 3.845789 | Up | casein alpha s1 |
| ILMN_1788955 | PDLIM1 | 0.78358 | 0.000725 | 0.000725 | 3.836228 | Up | PDZ and LIM domain 1 |
| ILMN_1696048 | MEDAG | 1.372503 | 0.000746 | 0.000746 | 3.825101 | Up | mesenteric estrogen dependent adipogenesis |
| ILMN_1667081 | CCND2 | 1.194174 | 0.000754 | 0.000754 | 3.82061 | Up | cyclin D2 |
| ILMN_1791447 | CXCL12 | 1.463874 | 0.000764 | 0.000764 | 3.815507 | Up | C-X-C motif chemokine ligand 12 |
| ILMN_1773650 | LRRN3 | 0.857534 | 0.000772 | 0.000772 | 3.81182 | Up | leucine rich repeat neuronal 3 |
| ILMN_1788098 | LHCGR | 0.89169 | 0.000777 | 0.000777 | 3.80896 | Up | luteinizing hormone/choriogonadotropin receptor |
| ILMN_1726245 | TGFBR2 | 0.593243 | 0.000793 | 0.000793 | 3.801026 | Up | transforming growth factor beta receptor 2 |
| ILMN_1711748 | PLTP | 0.575848 | 0.000794 | 0.000794 | 3.800825 | Up | phospholipid transfer protein |
| ILMN_2067656 | CCND2 | 1.120476 | 0.000809 | 0.000809 | 3.79331 | Up | cyclin D2 |
| ILMN_1695423 | CD9 | 0.740259 | 0.000811 | 0.000811 | 3.792273 | Up | CD9 molecule |
| ILMN_1689734 | IL1RN | 0.603248 | 0.000826 | 0.000826 | 3.785204 | Up | interleukin 1 receptor antagonist |
| ILMN_1682226 | CLDN15 | 0.574303 | 0.000834 | 0.000834 | 3.781751 | Up | claudin 15 |
| ILMN_1651354 | SPP1 | 1.473099 | 0.000848 | 0.000848 | 3.775351 | Up | secreted phosphoprotein 1 |
| ILMN_1701827 | CADM3 | 0.906754 | 0.000855 | 0.000855 | 3.771972 | Up | cell adhesion molecule 3 |
| ILMN_1762606 | AQP11 | 0.606742 | 0.000856 | 0.000856 | 3.77143 | Up | aquaporin 11 |
| ILMN_1752294 | PCDH9 | 0.795465 | 0.000895 | 0.000895 | 3.754302 | Up | protocadherin 9 |
| ILMN_1757604 | TPM2 | 1.097432 | 0.000924 | 0.000924 | 3.741629 | Up | tropomyosin 2 |
| ILMN_1846517 | ZNF404 | 0.539851 | 0.000927 | 0.000927 | 3.74066 | Up | zinc finger protein 404 |
| ILMN_1733756 | COL12A1 | 0.856512 | 0.000957 | 0.000957 | 3.728149 | Up | collagen type XII alpha 1 chain |
| ILMN_1775330 | CCDC9B | 0.541531 | 0.00096 | 0.00096 | 3.72698 | Up | coiled-coil domain containing 9B |
| ILMN_1727194 | CALU | 0.528913 | 0.001017 | 0.001017 | 3.704368 | Up | calumenin |
| ILMN_1689004 | TNFRSF12A | 0.747228 | 0.001026 | 0.001026 | 3.700896 | Up | TNF receptor superfamily member 12A |
| ILMN_1681983 | RSPO3 | 0.686707 | 0.001033 | 0.001033 | 3.698195 | Up | R-spondin 3 |
| ILMN_1659895 | MSN | 0.625448 | 0.001043 | 0.001043 | 3.694419 | Up | moesin |
| ILMN_2368318 | FGR | 0.658653 | 0.001059 | 0.001059 | 3.68866 | Up | FGR proto-oncogene, Src family tyrosine kinase |
| ILMN_1688780 | S100A4 | 1.113275 | 0.001085 | 0.001085 | 3.679083 | Up | S100 calcium binding protein A4 |
| ILMN_2326273 | CHI3L2 | 0.994947 | 0.001109 | 0.001109 | 3.670674 | Up | chitinase 3 like 2 |
| ILMN_1656910 | TRIM6 | 0.874617 | 0.00114 | 0.00114 | 3.65994 | Up | tripartite motif containing 6 |
| ILMN_1690464 | SLC35G1 | 0.621139 | 0.001146 | 0.001146 | 3.657985 | Up | solute carrier family 35 member G1 |
| ILMN_1680154 | MAP1B | 0.767967 | 0.001168 | 0.001168 | 3.650234 | Up | microtubule associated protein 1B |
| ILMN_1695432 | TPST2 | 0.631332 | 0.001178 | 0.001178 | 3.647137 | Up | tyrosylproteinsulfotransferase 2 |
| ILMN_1769556 | NPR3 | 1.277963 | 0.001204 | 0.001204 | 3.638396 | Up | natriuretic peptide receptor 3 |
| ILMN_1709630 | CCDC107 | 0.580296 | 0.001213 | 0.001213 | 3.635726 | Up | coiled-coil domain containing 107 |
| ILMN_1719938 | FGF11 | 0.526074 | 0.001215 | 0.001215 | 3.634907 | Up | fibroblast growth factor 11 |
| ILMN_1705442 | CMTM3 | 0.862194 | 0.001216 | 0.001216 | 3.634754 | Up | CKLF like MARVEL transmembrane domain containing 3 |
| ILMN_1809101 | STEAP2 | 0.596209 | 0.001232 | 0.001232 | 3.629353 | Up | STEAP2 metalloreductase |
| ILMN_2126038 | STMN2 | 1.000711 | 0.001248 | 0.001248 | 3.624508 | Up | stathmin 2 |
| ILMN_1716246 | FRZB | 1.171067 | 0.001249 | 0.001249 | 3.624283 | Up | frizzled related protein |
| ILMN_1765109 | TNFRSF25 | 0.935143 | 0.00125 | 0.00125 | 3.623681 | Up | TNF receptor superfamily member 25 |
| ILMN_1686116 | THBS1 | 0.92544 | 0.001267 | 0.001267 | 3.618613 | Up | thrombospondin 1 |
| ILMN_1703374 | NAV1 | 0.572426 | 0.001331 | 0.001331 | 3.599306 | Up | neuron navigator 1 |
| ILMN_2120247 | SLC2A10 | 0.724743 | 0.001347 | 0.001347 | 3.594626 | Up | solute carrier family 2 member 10 |
| ILMN_1774602 | FBLN2 | 0.732902 | 0.001389 | 0.001389 | 3.582362 | Up | fibulin 2 |
| ILMN_1720303 | OSTM1 | 0.544277 | 0.001419 | 0.001419 | 3.574118 | Up | osteoclastogenesis associated transmembrane protein 1 |
| ILMN_1728262 | SAA2 | 1.626006 | 0.001484 | 0.001484 | 3.556403 | Up | serum amyloid A2 |
| ILMN_1684306 | S100A4 | 1.095809 | 0.00154 | 0.00154 | 3.542006 | Up | S100 calcium binding protein A4 |
| ILMN_1767135 | SOS1 | 0.669373 | 0.001554 | 0.001554 | 3.538311 | Up | SOS Ras/Rac guanine nucleotide exchange factor 1 |
| ILMN_2311166 | ITGB5 | 0.677959 | 0.001588 | 0.001588 | 3.529759 | Up | integrin subunit beta 5 |
| ILMN_1780349 | PRG4 | 1.243154 | 0.001623 | 0.001623 | 3.521134 | Up | proteoglycan 4 |
| ILMN_1865764 | ZMAT3 | 0.587479 | 0.001638 | 0.001638 | 3.517465 | Up | zinc finger matrin-type 3 |
| ILMN_2049672 | ANO3 | 0.733106 | 0.00164 | 0.00164 | 3.517198 | Up | anoctamin 3 |
| ILMN_1723481 | CHST3 | 0.61328 | 0.001697 | 0.001697 | 3.503528 | Up | carbohydrate sulfotransferase 3 |
| ILMN_1730670 | FSTL3 | 0.886754 | 0.001749 | 0.001749 | 3.491738 | Up | follistatin like 3 |
| ILMN_1677603 | C1S | 0.559371 | 0.001764 | 0.001764 | 3.488236 | Up | complement C1s |
| ILMN_2389501 | HSD11B1 | 1.150839 | 0.001819 | 0.001819 | 3.476139 | Up | hydroxysteroid 11-beta dehydrogenase 1 |
| ILMN_2129505 | CYB561A3 | 0.524082 | 0.001892 | 0.001892 | 3.460542 | Up | cytochrome b561 family member A3 |
| ILMN_2385672 | ELN | 0.691623 | 0.001931 | 0.001931 | 3.452532 | Up | elastin |
| ILMN_1750790 | GSTM5 | 0.915719 | 0.002019 | 0.002019 | 3.434939 | Up | glutathione S-transferase mu 5 |
| ILMN_1691410 | BAMBI | 0.527637 | 0.002035 | 0.002035 | 3.431697 | Up | BMP and activin membrane bound inhibitor |
| ILMN_1761941 | FAM198B | 1.004094 | 0.002161 | 0.002161 | 3.407852 | Up | family with sequence similarity 198 member B |
| ILMN_1813561 | SCIN | 0.841973 | 0.002249 | 0.002249 | 3.392005 | Up | scinderin |
| ILMN_1874901 | MYMX | 1.099779 | 0.002337 | 0.002337 | 3.376776 | Up | myomixer, myoblast fusion factor |
| ILMN_2359907 | CD68 | 0.6105 | 0.00241 | 0.00241 | 3.364459 | Up | CD68 molecule |
| ILMN_1768940 | COL15A1 | 0.737179 | 0.002477 | 0.002477 | 3.35347 | Up | collagen type XV alpha 1 chain |
| ILMN_2208903 | CD52 | 1.060026 | 0.002573 | 0.002573 | 3.338374 | Up | CD52 molecule |
| ILMN_1791280 | HSPB8 | 0.744974 | 0.002774 | 0.002774 | 3.308162 | Up | heat shock protein family B (small) member 8 |
| ILMN_2374449 | SPP1 | 1.225401 | 0.0028 | 0.0028 | 3.304459 | Up | secreted phosphoprotein 1 |
| ILMN_1667893 | TNS3 | 0.580577 | 0.002814 | 0.002814 | 3.302498 | Up | tensin 3 |
| ILMN_2405642 | DHDDS | 0.75152 | 0.002815 | 0.002815 | 3.30234 | Up | dehydrodolichyldiphosphate synthase subunit |
| ILMN_1672124 | FAM198B | 0.855847 | 0.002832 | 0.002832 | 3.299928 | Up | family with sequence similarity 198 member B |
| ILMN_1675386 | CES1 | 1.290413 | 0.00284 | 0.00284 | 3.298759 | Up | carboxylesterase 1 |
| ILMN_1675797 | EPDR1 | 0.778794 | 0.002842 | 0.002842 | 3.298553 | Up | ependymin related 1 |
| ILMN_2302757 | FCGBP | 0.736052 | 0.002851 | 0.002851 | 3.297263 | Up | Fc fragment of IgG binding protein |
| ILMN_1662932 | LCP1 | 1.204997 | 0.002892 | 0.002892 | 3.291554 | Up | lymphocyte cytosolic protein 1 |
| ILMN_1762294 | ADAMTSL4 | 0.530835 | 0.002915 | 0.002915 | 3.288379 | Up | ADAMTS like 4 |
| ILMN_2207505 | LEP | 0.926283 | 0.002934 | 0.002934 | 3.285758 | Up | leptin |
| ILMN_1801710 | APBB1IP | 0.636573 | 0.002939 | 0.002939 | 3.285044 | Up | amyloid beta precursor protein binding family B member 1 interacting protein |
| ILMN_1688480 | CCND1 | 1.306118 | 0.002988 | 0.002988 | 3.278393 | Up | cyclin D1 |
| ILMN_1711566 | TIMP1 | 1.341538 | 0.003021 | 0.003021 | 3.274039 | Up | TIMP metallopeptidase inhibitor 1 |
| ILMN_2118663 | ERV3-1 | 0.661689 | 0.003035 | 0.003035 | 3.272124 | Up | endogenous retrovirus group 3 member 1, envelope |
| ILMN_1660691 | RAB31 | 0.709054 | 0.003132 | 0.003132 | 3.259457 | Up | RAB31, member RAS oncogene family |
| ILMN_2329679 | TPST2 | 0.55545 | 0.003374 | 0.003374 | 3.229456 | Up | tyrosylproteinsulfotransferase 2 |
| ILMN_1789535 | DHDDS | 0.745734 | 0.003401 | 0.003401 | 3.226338 | Up | dehydrodolichyldiphosphate synthase subunit |
| ILMN_1795963 | OSGIN1 | 0.67904 | 0.003407 | 0.003407 | 3.225533 | Up | oxidative stress induced growth inhibitor 1 |
| ILMN_1807925 | GNG2 | 0.834543 | 0.003615 | 0.003615 | 3.201662 | Up | G protein subunit gamma 2 |
| ILMN_2167915 | DSEL | 0.710109 | 0.003624 | 0.003624 | 3.200638 | Up | dermatansulfateepimerase like |
| ILMN_1774874 | IL1RN | 0.859928 | 0.003639 | 0.003639 | 3.198924 | Up | interleukin 1 receptor antagonist |
| ILMN_1689111 | CXCL12 | 0.954243 | 0.003689 | 0.003689 | 3.193422 | Up | C-X-C motif chemokine ligand 12 |
| ILMN_1779875 | THY1 | 1.139729 | 0.003871 | 0.003871 | 3.17396 | Up | Thy-1 cell surface antigen |
| ILMN_1720282 | NQO1 | 1.254269 | 0.003892 | 0.003892 | 3.171678 | Up | NAD(P)H quinone dehydrogenase 1 |
| ILMN_2132982 | IGFBP5 | 0.905666 | 0.003894 | 0.003894 | 3.171544 | Up | insulin like growth factor binding protein 5 |
| ILMN_1667948 | SPATA18 | 0.587846 | 0.003905 | 0.003905 | 3.170399 | Up | spermatogenesis associated 18 |
| ILMN_1729117 | COL5A2 | 0.640357 | 0.004082 | 0.004082 | 3.152419 | Up | collagen type V alpha 2 chain |
| ILMN_1668629 | C4orf48 | 0.583778 | 0.004123 | 0.004123 | 3.148274 | Up | chromosome 4 open reading frame 48 |
| ILMN_1661755 | FAM129B | 0.559501 | 0.004167 | 0.004167 | 3.144004 | Up | family with sequence similarity 129 member B |
| ILMN_1815205 | LYZ | 0.888701 | 0.004176 | 0.004176 | 3.143076 | Up | lysozyme |
| ILMN_1676563 | HTRA1 | 0.717816 | 0.00422 | 0.00422 | 3.138853 | Up | HtrA serine peptidase 1 |
| ILMN_1739161 | PLPP1 | 0.659073 | 0.004346 | 0.004346 | 3.126844 | Up | phospholipid phosphatase 1 |
| ILMN_1718198 | NPY5R | 0.680103 | 0.004442 | 0.004442 | 3.117994 | Up | neuropeptide Y receptor Y5 |
| ILMN_1700268 | QPRT | 0.699906 | 0.004533 | 0.004533 | 3.109706 | Up | quinolinatephosphoribosyltransferase |
| ILMN_1754795 | FAT1 | 0.577636 | 0.00456 | 0.00456 | 3.107242 | Up | FAT atypical cadherin 1 |
| ILMN_2070521 | BHMT2 | 0.720849 | 0.004718 | 0.004718 | 3.093369 | Up | betaine--homocysteine S-methyltransferase 2 |
| ILMN_2408748 | SLC22A12 | 0.712622 | 0.004807 | 0.004807 | 3.085718 | Up | solute carrier family 22 member 12 |
| ILMN_1808732 | SAA1 | 1.188117 | 0.004821 | 0.004821 | 3.084499 | Up | serum amyloid A1 |
| ILMN_1676213 | SRPX2 | 0.800783 | 0.005117 | 0.005117 | 3.060079 | Up | sushi repeat containing protein X-linked 2 |
| ILMN_2359945 | CES1 | 1.12408 | 0.005165 | 0.005165 | 3.056273 | Up | carboxylesterase 1 |
| ILMN_1681886 | ADAMTS5 | 0.669885 | 0.005448 | 0.005448 | 3.034336 | Up | ADAM metallopeptidase with thrombospondin type 1 motif 5 |
| ILMN_2198413 | MYEOV | 0.990449 | 0.005456 | 0.005456 | 3.033789 | Up | myeloma overexpressed |
| ILMN_1772821 | KIAA1671 | 0.625167 | 0.0056 | 0.0056 | 3.02307 | Up | KIAA1671 |
| ILMN_1735877 | EFEMP1 | 0.530826 | 0.005622 | 0.005622 | 3.021426 | Up | EGF containing fibulin extracellular matrix protein 1 |
| ILMN_1769282 | FRMD6 | 0.590922 | 0.005627 | 0.005627 | 3.021047 | Up | FERM domain containing 6 |
| ILMN_1671337 | SLC2A5 | 0.819888 | 0.005673 | 0.005673 | 3.01771 | Up | solute carrier family 2 member 5 |
| ILMN_1745954 | CORO1C | 0.555758 | 0.005891 | 0.005891 | 3.00216 | Up | coronin 1C |
| ILMN_1813043 | BHMT2 | 0.656478 | 0.005947 | 0.005947 | 2.998245 | Up | betaine--homocysteine S-methyltransferase 2 |
| ILMN_2413816 | GRB14 | 0.547963 | 0.006056 | 0.006056 | 2.990752 | Up | growth factor receptor bound protein 14 |
| ILMN_1696187 | PYGL | 0.644613 | 0.006112 | 0.006112 | 2.986947 | Up | glycogen phosphorylase L |
| ILMN_2288483 | CEP19 | 0.554845 | 0.006145 | 0.006145 | 2.984691 | Up | centrosomal protein 19 |
| ILMN_1792409 | AMOT | 0.816144 | 0.006593 | 0.006593 | 2.95555 | Up | angiomotin |
| ILMN_1704656 | PPP2R1B | 0.907097 | 0.00667 | 0.00667 | 2.950717 | Up | protein phosphatase 2 scaffold subunit Abeta |
| ILMN_1663519 | SLC24A3 | 0.633922 | 0.006681 | 0.006681 | 2.950063 | Up | solute carrier family 24 member 3 |
| ILMN_1785272 | COL1A2 | 1.193362 | 0.006691 | 0.006691 | 2.949439 | Up | collagen type I alpha 2 chain |
| ILMN_1743130 | PTGFRN | 0.524934 | 0.006893 | 0.006893 | 2.937051 | Up | prostaglandin F2 receptor inhibitor |
| ILMN_1684554 | COL16A1 | 0.61773 | 0.006913 | 0.006913 | 2.935844 | Up | collagen type XVI alpha 1 chain |
| ILMN_1912662 | #N/A | 0.754156 | 0.006937 | 0.006937 | 2.934422 | Up | NA |
| ILMN_1750324 | IGFBP5 | 0.747781 | 0.006964 | 0.006964 | 2.93278 | Up | insulin like growth factor binding protein 5 |
| ILMN_2389506 | HSD11B1 | 0.984125 | 0.007022 | 0.007022 | 2.929321 | Up | hydroxysteroid 11-beta dehydrogenase 1 |
| ILMN_1743836 | MXRA7 | 0.631725 | 0.007077 | 0.007077 | 2.926107 | Up | matrix remodeling associated 7 |
| ILMN_1810172 | SFRP4 | 1.030645 | 0.007248 | 0.007248 | 2.91613 | Up | secreted frizzled related protein 4 |
| ILMN_1901314 | MIR3188 | 0.733061 | 0.007283 | 0.007283 | 2.91414 | Up | microRNA 3188 |
| ILMN_1779182 | TMEM98 | 0.794433 | 0.007292 | 0.007292 | 2.913633 | Up | transmembrane protein 98 |
| ILMN_1790859 | PLAC9 | 1.050248 | 0.00741 | 0.00741 | 2.906899 | Up | placenta specific 9 |
| ILMN_1711092 | KCNB1 | 0.551283 | 0.007446 | 0.007446 | 2.904916 | Up | potassium voltage-gated channel subfamily B member 1 |
| ILMN_2090105 | TAGLN2 | 0.596354 | 0.007534 | 0.007534 | 2.900001 | Up | transgelin 2 |
| ILMN_2183409 | SCARB1 | 0.663206 | 0.007717 | 0.007717 | 2.889966 | Up | scavenger receptor class B member 1 |
| ILMN_2104356 | COL1A2 | 1.147573 | 0.007881 | 0.007881 | 2.881147 | Up | collagen type I alpha 2 chain |
| ILMN_1764109 | C1R | 0.857406 | 0.007884 | 0.007884 | 2.881016 | Up | complement C1r |
| ILMN_1773389 | PLTP | 0.698948 | 0.007931 | 0.007931 | 2.878531 | Up | phospholipid transfer protein |
| ILMN_2317364 | CSN1S1 | 0.754633 | 0.008037 | 0.008037 | 2.872918 | Up | casein alpha s1 |
| ILMN_1811370 | HSD11B1 | 0.987491 | 0.008232 | 0.008232 | 2.862873 | Up | hydroxysteroid 11-beta dehydrogenase 1 |
| ILMN_1715748 | FLNC | 0.938831 | 0.008274 | 0.008274 | 2.860761 | Up | filamin C |
| ILMN_1702487 | SGK1 | 0.776167 | 0.008467 | 0.008467 | 2.85108 | Up | serum/glucocorticoid regulated kinase 1 |
| ILMN_1785732 | TNFAIP6 | 0.814063 | 0.008644 | 0.008644 | 2.842376 | Up | TNF alpha induced protein 6 |
| ILMN_1752968 | LAMB2 | 0.525558 | 0.00872 | 0.00872 | 2.838678 | Up | laminin subunit beta 2 |
| ILMN_1739428 | IFIT2 | 0.526187 | 0.008742 | 0.008742 | 2.837639 | Up | interferon induced protein with tetratricopeptide repeats 2 |
| ILMN_1795930 | PTGER4 | 0.559346 | 0.008844 | 0.008844 | 2.832708 | Up | prostaglandin E receptor 4 |
| ILMN_2207504 | LEP | 0.982719 | 0.009035 | 0.009035 | 2.823736 | Up | leptin |
| ILMN_1680948 | LMOD1 | 0.695953 | 0.009262 | 0.009262 | 2.813224 | Up | leiomodin 1 |
| ILMN_2361862 | VLDLR | 0.854371 | 0.009369 | 0.009369 | 2.808386 | Up | very low density lipoprotein receptor |
| ILMN_1677723 | ANGPT1 | 0.555038 | 0.009564 | 0.009564 | 2.799696 | Up | angiopoietin 1 |
| ILMN_1779841 | PPP2R1B | 0.853222 | 0.00961 | 0.00961 | 2.797666 | Up | protein phosphatase 2 scaffold subunit Abeta |
| ILMN_2086890 | ANGPT1 | 0.538832 | 0.009689 | 0.009689 | 2.794185 | Up | angiopoietin 1 |
| ILMN_1668345 | OAF | 0.706992 | 0.009694 | 0.009694 | 2.793955 | Up | out at first homolog |
| ILMN_1712639 | AIFM2 | 0.528075 | 0.009914 | 0.009914 | 2.784441 | Up | apoptosis inducing factor, mitochondria associated 2 |
| ILMN_2175912 | ITGB2 | 0.995891 | 0.009957 | 0.009957 | 2.782636 | Up | integrin subunit beta 2 |
| ILMN_2304512 | SAA1 | 1.23664 | 0.010239 | 0.010239 | 2.770776 | Up | serum amyloid A1 |
| ILMN_1803213 | MXRA5 | 1.316927 | 0.010301 | 0.010301 | 2.768192 | Up | matrix remodeling associated 5 |
| ILMN_2128770 | CDR2L | 0.648047 | 0.010378 | 0.010378 | 2.765041 | Up | cerebellar degeneration related protein 2 like |
| ILMN_1699829 | CTGF | 1.157229 | 0.010714 | 0.010714 | 2.751485 | Up | connective tissue growth factor |
| ILMN_1668065 | ELMOD3 | 0.623974 | 0.011063 | 0.011063 | 2.737816 | Up | ELMO domain containing 3 |
| ILMN_2400935 | TAGLN | 1.014014 | 0.012046 | 0.012046 | 2.701368 | Up | transgelin |
| ILMN_1814917 | TLE2 | 0.597486 | 0.012167 | 0.012167 | 2.69709 | Up | transducin like enhancer of split 2 |
| ILMN_2085862 | SLC15A3 | 0.636791 | 0.01239 | 0.01239 | 2.689276 | Up | solute carrier family 15 member 3 |
| ILMN_1733094 | STEAP1 | 0.592088 | 0.012408 | 0.012408 | 2.688656 | Up | STEAP family member 1 |
| ILMN_1791759 | CXCL10 | 0.657731 | 0.012449 | 0.012449 | 2.687253 | Up | C-X-C motif chemokine ligand 10 |
| ILMN_1776936 | KANK4 | 0.876463 | 0.012729 | 0.012729 | 2.677674 | Up | KN motif and ankyrin repeat domains 4 |
| ILMN_1800078 | LMO2 | 0.63765 | 0.012879 | 0.012879 | 2.672643 | Up | LIM domain only 2 |
| ILMN_2115125 | CTGF | 1.120845 | 0.012951 | 0.012951 | 2.670237 | Up | connective tissue growth factor |
| ILMN_1654396 | ITGB2 | 0.895679 | 0.013318 | 0.013318 | 2.658192 | Up | integrin subunit beta 2 |
| ILMN_1701017 | SAA1 | 1.464529 | 0.013581 | 0.013581 | 2.64974 | Up | serum amyloid A1 |
| ILMN_2285713 | TDP1 | 0.644122 | 0.013593 | 0.013593 | 2.649359 | Up | tyrosyl-DNA phosphodiesterase 1 |
| ILMN_1735453 | FAM98A | 0.663756 | 0.013712 | 0.013712 | 2.645589 | Up | family with sequence similarity 98 member A |
| ILMN_1803094 | PDGFD | 0.783158 | 0.014521 | 0.014521 | 2.620732 | Up | platelet derived growth factor D |
| ILMN_2048591 | LRRN3 | 0.742029 | 0.014574 | 0.014574 | 2.619167 | Up | leucine rich repeat neuronal 3 |
| ILMN_1665033 | NPR3 | 0.645778 | 0.014643 | 0.014643 | 2.617101 | Up | natriuretic peptide receptor 3 |
| ILMN_1780663 | KLHL30 | 0.558729 | 0.014833 | 0.014833 | 2.611491 | Up | kelch like family member 30 |
| ILMN_1657697 | SAR1A | 0.634135 | 0.014959 | 0.014959 | 2.607818 | Up | secretion associated Ras related GTPase 1A |
| ILMN_1778668 | TAGLN | 0.906221 | 0.015205 | 0.015205 | 2.600719 | Up | transgelin |
| ILMN_1715189 | LHX6 | 0.565688 | 0.015316 | 0.015316 | 2.597543 | Up | LIM homeobox 6 |
| ILMN_1714861 | CD68 | 0.675226 | 0.015872 | 0.015872 | 2.582001 | Up | CD68 molecule |
| ILMN_2150095 | CES1 | 0.984298 | 0.015943 | 0.015943 | 2.580041 | Up | carboxylesterase 1 |
| ILMN_2214473 | ARHGEF35 | 0.811545 | 0.016232 | 0.016232 | 2.572178 | Up | Rho guanine nucleotide exchange factor 35 |
| ILMN_2376859 | PDGFD | 0.719127 | 0.016976 | 0.016976 | 2.552558 | Up | platelet derived growth factor D |
| ILMN_1686664 | MT2A | 0.710372 | 0.017665 | 0.017665 | 2.535057 | Up | metallothionein 2A |
| ILMN_1756071 | MFGE8 | 0.547292 | 0.017825 | 0.017825 | 2.531091 | Up | milk fat globule-EGF factor 8 protein |
| ILMN_1672660 | MBP | 0.589996 | 0.018136 | 0.018136 | 2.523473 | Up | myelin basic protein |
| ILMN_2390919 | FBLN2 | 0.618463 | 0.018757 | 0.018757 | 2.508612 | Up | fibulin 2 |
| ILMN_2129161 | LRRC32 | 0.633777 | 0.019006 | 0.019006 | 2.50277 | Up | leucine rich repeat containing 32 |
| ILMN_2077952 | GALNT16 | 0.693338 | 0.019332 | 0.019332 | 2.495234 | Up | polypeptide N-acetylgalactosaminyltransferase 16 |
| ILMN_2188264 | CYR61 | 0.614752 | 0.019359 | 0.019359 | 2.49463 | Up | cysteine rich angiogenic inducer 61 |
| ILMN_2081087 | HSPA12A | 0.712069 | 0.019366 | 0.019366 | 2.494464 | Up | heat shock protein family A (Hsp70) member 12A |
| ILMN_1731374 | CPE | 0.684523 | 0.019703 | 0.019703 | 2.486818 | Up | carboxypeptidase E |
| ILMN_1790529 | LUM | 1.011109 | 0.019825 | 0.019825 | 2.484067 | Up | lumican |
| ILMN_1690170 | CRABP2 | 0.621015 | 0.019878 | 0.019878 | 2.482893 | Up | cellular retinoic acid binding protein 2 |
| ILMN_2112638 | SVEP1 | 0.620822 | 0.020031 | 0.020031 | 2.479476 | Up | sushi, von Willebrand factor type A, EGF and pentraxin domain containing 1 |
| ILMN_1745356 | CXCL9 | 0.621326 | 0.020666 | 0.020666 | 2.465596 | Up | C-X-C motif chemokine ligand 9 |
| ILMN_1707337 | MSTO1 | 0.52744 | 0.020761 | 0.020761 | 2.463566 | Up | misato 1, mitochondrial distribution and morphology regulator |
| ILMN_1677198 | C1R | 0.747849 | 0.020785 | 0.020785 | 2.46305 | Up | complement C1r |
| ILMN_1773567 | LAMA5 | 0.597756 | 0.021626 | 0.021626 | 2.445353 | Up | laminin subunit alpha 5 |
| ILMN_1880143 | #N/A | 1.313752 | 0.022328 | 0.022328 | 2.431054 | Up | NA |
| ILMN_1778977 | TYROBP | 0.880575 | 0.023449 | 0.023449 | 2.409065 | Up | TYRO protein tyrosine kinase binding protein |
| ILMN_1789639 | FMOD | 0.551304 | 0.023716 | 0.023716 | 2.403955 | Up | fibromodulin |
| ILMN_1767113 | AOX1 | 0.568405 | 0.023837 | 0.023837 | 2.401671 | Up | aldehyde oxidase 1 |
| ILMN_1743199 | EGR2 | 0.837499 | 0.024408 | 0.024408 | 2.391003 | Up | early growth response 2 |
| ILMN_2137789 | KLF4 | 0.69295 | 0.024585 | 0.024585 | 2.387746 | Up | Kruppel like factor 4 |
| ILMN_1656920 | CRIP1 | 0.949702 | 0.024669 | 0.024669 | 2.386203 | Up | cysteine rich protein 1 |
| ILMN_1750100 | TUBB8 | 0.542127 | 0.024671 | 0.024671 | 2.386169 | Up | tubulin beta 8 class VIII |
| ILMN_2143795 | CYTOR | 0.653327 | 0.025094 | 0.025094 | 2.37848 | Up | cytoskeleton regulator RNA |
| ILMN_1773313 | ATP5MD | 0.888141 | 0.025158 | 0.025158 | 2.377325 | Up | ATP synthase membrane subunit DAPIT |
| ILMN_1807042 | MARCKS | 0.527377 | 0.025383 | 0.025383 | 2.373296 | Up | myristoylated alanine rich protein kinase C substrate |
| ILMN_1750974 | S100A9 | 0.533811 | 0.025625 | 0.025625 | 2.369003 | Up | S100 calcium binding protein A9 |
| ILMN_2134538 | FTH1 | 0.660487 | 0.02646 | 0.02646 | 2.354446 | Up | ferritin heavy chain 1 |
| ILMN_1742332 | KCTD12 | 0.622266 | 0.027085 | 0.027085 | 2.343832 | Up | potassium channel tetramerization domain containing 12 |
| ILMN_1810910 | CFH | 0.588853 | 0.027275 | 0.027275 | 2.340649 | Up | complement factor H |
| ILMN_1667796 | HBA1 | 1.236166 | 0.027548 | 0.027548 | 2.336103 | Up | hemoglobin subunit alpha 1 |
| ILMN_2073235 | FTH1 | 0.608036 | 0.028444 | 0.028444 | 2.321488 | Up | ferritin heavy chain 1 |
| ILMN_1774287 | CFB | 0.599443 | 0.029185 | 0.029185 | 2.309725 | Up | complement factor B |
| ILMN_2125010 | SKAP2 | 0.539815 | 0.029275 | 0.029275 | 2.308306 | Up | src kinase associated phosphoprotein 2 |
| ILMN_2173835 | FTH1P3 | 0.662782 | 0.029605 | 0.029605 | 2.303169 | Up | ferritin heavy chain 1 pseudogene 3 |
| ILMN_1734276 | PMEPA1 | 0.531815 | 0.030569 | 0.030569 | 2.288446 | Up | prostate transmembrane protein, androgen induced 1 |
| ILMN_1797776 | PRSS23 | 0.559654 | 0.031299 | 0.031299 | 2.277572 | Up | serine protease 23 |
| ILMN_2100437 | HBB | 1.198522 | 0.032716 | 0.032716 | 2.257103 | Up | hemoglobin subunit beta |
| ILMN_1730906 | FILIP1L | 0.558741 | 0.03308 | 0.03308 | 2.251975 | Up | filamin A interacting protein 1 like |
| ILMN_1746525 | FTH1 | 0.659996 | 0.033776 | 0.033776 | 2.242313 | Up | ferritin heavy chain 1 |
| ILMN_2412192 | CFH | 0.617233 | 0.0349 | 0.0349 | 2.227088 | Up | complement factor H |
| ILMN_1699665 | CLIC6 | 1.048527 | 0.035707 | 0.035707 | 2.216421 | Up | chloride intracellular channel 6 |
| ILMN_1663490 | ZNF541 | 0.56762 | 0.036403 | 0.036403 | 2.207405 | Up | zinc finger protein 541 |
| ILMN_2207328 | TPGS2 | 0.6484 | 0.036764 | 0.036764 | 2.202786 | Up | tubulin polyglutamylase complex subunit 2 |
| ILMN_1725090 | CTHRC1 | 0.710406 | 0.038042 | 0.038042 | 2.186755 | Up | collagen triple helix repeat containing 1 |
| ILMN_2105573 | CCL3L3 | 0.989332 | 0.039486 | 0.039486 | 2.169215 | Up | C-C motif chemokine ligand 3 like 3 |
| ILMN_1668055 | SAA4 | 0.818678 | 0.040036 | 0.040036 | 2.162689 | Up | serum amyloid A4, constitutive |
| ILMN_2203950 | HLA-A | 0.552025 | 0.040908 | 0.040908 | 2.152498 | Up | major histocompatibility complex, class I, A |
| ILMN_1803033 | MGST1 | 0.565499 | 0.041608 | 0.041608 | 2.144465 | Up | microsomal glutathione S-transferase 1 |
| ILMN_1801205 | GPNMB | 0.87457 | 0.041612 | 0.041612 | 2.144418 | Up | glycoprotein nmb |
| ILMN_1748840 | CALB2 | 0.575885 | 0.044509 | 0.044509 | 2.112392 | Up | calbindin 2 |
| ILMN_1696911 | FTH1 | 0.76525 | 0.044581 | 0.044581 | 2.111618 | Up | ferritin heavy chain 1 |
| ILMN_2309156 | PMEPA1 | 0.578206 | 0.044785 | 0.044785 | 2.10944 | Up | prostate transmembrane protein, androgen induced 1 |
| ILMN_1661196 | CSF2RA | 0.652935 | 0.046014 | 0.046014 | 2.096479 | Up | colony stimulating factor 2 receptor alpha subunit |
| ILMN_2376455 | CSF2RA | 0.670921 | 0.046639 | 0.046639 | 2.090007 | Up | colony stimulating factor 2 receptor alpha subunit |
| ILMN_2391150 | FILIP1L | 0.541279 | 0.048499 | 0.048499 | 2.071199 | Up | filamin A interacting protein 1 like |
| ILMN_1738578 | FILIP1L | 0.649489 | 0.048772 | 0.048772 | 2.068499 | Up | filamin A interacting protein 1 like |
| ILMN_1705676 | MRAP | 0.740128 | 0.048781 | 0.048781 | 2.06841 | Up | melanocortin 2 receptor accessory protein |
| ILMN_1706013 | FTH1 | 0.538663 | 0.049065 | 0.049065 | 2.065608 | Up | ferritin heavy chain 1 |
| ILMN_1764714 | ANGPTL8 | 0.674487 | 0.049616 | 0.049616 | 2.060209 | Up | angiopoietin like 8 |
| ILMN_1700183 | APLNR | 0.60085 | 0.049861 | 0.049861 | 2.057827 | Up | apelin receptor |
| ILMN_1682176 | CLEC3B | 0.756039 | 0.049876 | 0.049876 | 2.057683 | Up | C-type lectin domain family 3 member B |
| ILMN_1684227 | GPR146 | -0.83595 | 1.71E-09 | 1.71E-09 | -9.06509 | Down | G protein-coupled receptor 146 |
| ILMN_1821176 | RNF125 | -1.20288 | 2.73E-09 | 2.73E-09 | -8.85291 | Down | ring finger protein 125 |
| ILMN_1694514 | ZDHHC11 | -1.16835 | 1.08E-08 | 1.08E-08 | -8.24436 | Down | zinc finger DHHC-type containing 11 |
| ILMN_2298818 | RPS29 | -0.68843 | 6.63E-08 | 6.63E-08 | -7.46988 | Down | ribosomal protein S29 |
| ILMN_1662188 | WNT11 | -0.68384 | 7.34E-08 | 7.34E-08 | -7.42761 | Down | Wnt family member 11 |
| ILMN_1705346 | NBEA | -0.72037 | 9.33E-08 | 9.33E-08 | -7.32789 | Down | neurobeachin |
| ILMN_1881960 | HDAC9 | -1.17786 | 2.12E-07 | 2.12E-07 | -6.9912 | Down | histone deacetylase 9 |
| ILMN_1725787 | RFX1 | -0.40524 | 2.68E-07 | 2.68E-07 | -6.89627 | Down | regulatory factor X1 |
| ILMN_2396287 | RFX2 | -0.56092 | 2.76E-07 | 2.76E-07 | -6.88354 | Down | regulatory factor X2 |
| ILMN_2175465 | RSL24D1 | -0.46858 | 3.6E-07 | 3.6E-07 | -6.77716 | Down | ribosomal L24 domain containing 1 |
| ILMN_1730794 | SERTAD4 | -0.89762 | 1.28E-06 | 1.28E-06 | -6.27275 | Down | SERTA domain containing 4 |
| ILMN_1752810 | LARP6 | -0.78114 | 2.04E-06 | 2.04E-06 | -6.08902 | Down | La ribonucleoprotein domain family member 6 |
| ILMN_2184966 | ZHX2 | -0.82429 | 2.19E-06 | 2.19E-06 | -6.06085 | Down | zinc fingers and homeoboxes 2 |
| ILMN_2352563 | CLDND1 | -0.40042 | 2.91E-06 | 2.91E-06 | -5.95008 | Down | claudin domain containing 1 |
| ILMN_1691559 | ELF2 | -0.65194 | 3.12E-06 | 3.12E-06 | -5.92228 | Down | E74 like ETS transcription factor 2 |
| ILMN_1749821 | MED28 | -0.52157 | 3.69E-06 | 3.69E-06 | -5.85719 | Down | mediator complex subunit 28 |
| ILMN_2389347 | NR3C1 | -0.55108 | 3.74E-06 | 3.74E-06 | -5.85196 | Down | nuclear receptor subfamily 3 group C member 1 |
| ILMN_1789508 | GTF3C3 | -0.5818 | 4.23E-06 | 4.23E-06 | -5.8044 | Down | general transcription factor IIIC subunit 3 |
| ILMN_2295252 | C9orf72 | -0.46781 | 4.56E-06 | 4.56E-06 | -5.77509 | Down | chromosome 9 open reading frame 72 |
| ILMN_1757183 | ZFHX4-AS1 | -0.70437 | 5.53E-06 | 5.53E-06 | -5.70054 | Down | ZFHX4 antisense RNA 1 |
| ILMN_2346727 | MTUS1 | -0.62796 | 5.6E-06 | 5.6E-06 | -5.69632 | Down | microtubule associated scaffold protein 1 |
| ILMN_1666208 | MIS18BP1 | -0.42342 | 6.97E-06 | 6.97E-06 | -5.61133 | Down | MIS18 binding protein 1 |
| ILMN_1722829 | HLF | -1.04562 | 7.13E-06 | 7.13E-06 | -5.60276 | Down | HLF, PAR bZIP transcription factor |
| ILMN_1790973 | CDS2 | -0.45775 | 7.13E-06 | 7.13E-06 | -5.6027 | Down | CDP-diacylglycerol synthase 2 |
| ILMN_1694106 | GPD1L | -1.21248 | 7.83E-06 | 7.83E-06 | -5.56674 | Down | glycerol-3-phosphate dehydrogenase 1 like |
| ILMN_1735743 | FBLN7 | -0.70811 | 8.05E-06 | 8.05E-06 | -5.55624 | Down | fibulin 7 |
| ILMN_1792951 | ZHX2 | -0.59218 | 1.03E-05 | 1.03E-05 | -5.46294 | Down | zinc fingers and homeoboxes 2 |
| ILMN_1746658 | RORB | -1.44876 | 1.04E-05 | 1.04E-05 | -5.45785 | Down | RAR related orphan receptor B |
| ILMN_1747119 | FBXO46 | -0.49642 | 1.11E-05 | 1.11E-05 | -5.43233 | Down | F-box protein 46 |
| ILMN_1722329 | CASQ2 | -1.56426 | 1.14E-05 | 1.14E-05 | -5.42341 | Down | calsequestrin 2 |
| ILMN_1706706 | DCAF7 | -0.42387 | 1.21E-05 | 1.21E-05 | -5.39933 | Down | DDB1 and CUL4 associated factor 7 |
| ILMN_1730907 | ZFAND1 | -0.60649 | 1.26E-05 | 1.26E-05 | -5.38283 | Down | zinc finger AN1-type containing 1 |
| ILMN_1735038 | MARCH3 | -0.53104 | 1.4E-05 | 1.4E-05 | -5.34512 | Down | membrane associated ring-CH-type finger 3 |
| ILMN_1681780 | MKX | -1.37991 | 1.44E-05 | 1.44E-05 | -5.33293 | Down | mohawkhomeobox |
| ILMN_1797154 | AZGP1 | -1.37572 | 1.72E-05 | 1.72E-05 | -5.26426 | Down | alpha-2-glycoprotein 1, zinc-binding |
| ILMN_1726547 | MAP3K5 | -0.8941 | 1.77E-05 | 1.77E-05 | -5.2531 | Down | mitogen-activated protein kinase kinasekinase 5 |
| ILMN_1799289 | MRPL55 | -0.4503 | 1.83E-05 | 1.83E-05 | -5.24231 | Down | mitochondrial ribosomal protein L55 |
| ILMN_1782609 | STAG2 | -0.41892 | 1.88E-05 | 1.88E-05 | -5.23192 | Down | stromal antigen 2 |
| ILMN_1800451 | MED16 | -0.60494 | 1.96E-05 | 1.96E-05 | -5.21497 | Down | mediator complex subunit 16 |
| ILMN_1778523 | KLF9 | -0.92493 | 2.2E-05 | 2.2E-05 | -5.17137 | Down | Kruppel like factor 9 |
| ILMN_1708059 | USP13 | -0.51604 | 2.49E-05 | 2.49E-05 | -5.12403 | Down | ubiquitin specific peptidase 13 |
| ILMN_1760727 | ANG | -0.78319 | 2.55E-05 | 2.55E-05 | -5.11517 | Down | angiogenin |
| ILMN_1751143 | TMEM243 | -0.63607 | 2.56E-05 | 2.56E-05 | -5.11254 | Down | transmembrane protein 243 |
| ILMN_1760490 | ACVR1 | -0.62337 | 2.65E-05 | 2.65E-05 | -5.09947 | Down | activin A receptor type 1 |
| ILMN_1790782 | MED16 | -0.63172 | 2.75E-05 | 2.75E-05 | -5.08654 | Down | mediator complex subunit 16 |
| ILMN_1748578 | RAD21 | -0.71483 | 3.08E-05 | 3.08E-05 | -5.04321 | Down | RAD21 cohesin complex component |
| ILMN_1662935 | C1QTNF7 | -0.56926 | 3.72E-05 | 3.72E-05 | -4.97056 | Down | C1q and TNF related 7 |
| ILMN_1660451 | STARD13 | -0.4495 | 3.89E-05 | 3.89E-05 | -4.95347 | Down | StAR related lipid transfer domain containing 13 [ |
| ILMN_1655497 | EIF4B | -0.52825 | 3.95E-05 | 3.95E-05 | -4.94802 | Down | eukaryotic translation initiation factor 4B |
| ILMN_2076640 | KHDRBS1 | -0.45183 | 3.96E-05 | 3.96E-05 | -4.94687 | Down | KH RNA binding domain containing, signal transduction associated 1 |
| ILMN_1770247 | HOXA10 | -0.51668 | 3.97E-05 | 3.97E-05 | -4.94619 | Down | homeobox A10 |
| ILMN_1652549 | DTNA | -0.6919 | 4.46E-05 | 4.46E-05 | -4.90175 | Down | dystrobrevin alpha |
| ILMN_1811367 | MAT2B | -0.4369 | 4.69E-05 | 4.69E-05 | -4.88274 | Down | methionine adenosyltransferase 2B |
| ILMN_1730201 | DTNA | -0.89589 | 4.73E-05 | 4.73E-05 | -4.87937 | Down | dystrobrevin alpha |
| ILMN_1810628 | PRUNE2 | -0.98344 | 5.62E-05 | 5.62E-05 | -4.81407 | Down | prune homolog 2 |
| ILMN_1677396 | NDFIP2 | -0.46666 | 5.7E-05 | 5.7E-05 | -4.80812 | Down | Nedd4 family interacting protein 2 |
| ILMN_1738095 | PER2 | -0.92194 | 6.05E-05 | 6.05E-05 | -4.78554 | Down | period circadian regulator 2 |
| ILMN_2103841 | AIP | -0.45066 | 6.73E-05 | 6.73E-05 | -4.74505 | Down | aryl hydrocarbon receptor interacting protein |
| ILMN_1724349 | RGS3 | -0.74322 | 6.74E-05 | 6.74E-05 | -4.74458 | Down | regulator of G protein signaling 3 |
| ILMN_1654268 | HMGB2 | -0.80135 | 7.07E-05 | 7.07E-05 | -4.72659 | Down | high mobility group box 2 |
| ILMN_1740996 | CA3 | -1.83227 | 7.42E-05 | 7.42E-05 | -4.70796 | Down | carbonic anhydrase 3 |
| ILMN_1763311 | SPX | -1.62598 | 7.43E-05 | 7.43E-05 | -4.70777 | Down | spexin hormone |
| ILMN_2262288 | EEF1G | -0.60713 | 7.48E-05 | 7.48E-05 | -4.70498 | Down | eukaryotic translation elongation factor 1 gamma |
| ILMN_2347349 | CCNB1IP1 | -0.62741 | 7.53E-05 | 7.53E-05 | -4.70247 | Down | cyclin B1 interacting protein 1 |
| ILMN_1807514 | ZNF394 | -0.39537 | 7.9E-05 | 7.9E-05 | -4.68415 | Down | zinc finger protein 394 |
| ILMN_1672908 | TWIST1 | -0.85589 | 8.24E-05 | 8.24E-05 | -4.66846 | Down | twist family bHLH transcription factor 1 |
| ILMN_1808157 | RUNDC3B | -0.49287 | 8.27E-05 | 8.27E-05 | -4.66671 | Down | RUN domain containing 3B |
| ILMN_1729180 | GATM | -0.51604 | 9.08E-05 | 9.08E-05 | -4.63121 | Down | glycine amidinotransferase |
| ILMN_1722811 | CDKN1B | -0.64659 | 9.65E-05 | 9.65E-05 | -4.60803 | Down | cyclin dependent kinase inhibitor 1B |
| ILMN_1738047 | FAM170B | -0.56504 | 9.94E-05 | 9.94E-05 | -4.59684 | Down | family with sequence similarity 170 member B |
| ILMN_1804735 | CBS | -0.52494 | 0.0001 | 0.0001 | -4.59345 | Down | cystathionine-beta-synthase |
| ILMN_1691611 | HNRNPA1P10 | -0.7512 | 0.000101 | 0.000101 | -4.58978 | Down | heterogeneous nuclear ribonucleoprotein A1 pseudogene 10 |
| ILMN_1654543 | MED6 | -0.42696 | 0.000102 | 0.000102 | -4.58768 | Down | mediator complex subunit 6 |
| ILMN_1749447 | RPS9 | -0.54354 | 0.000104 | 0.000104 | -4.57948 | Down | ribosomal protein S9 |
| ILMN_1798164 | PHF3 | -0.57458 | 0.000109 | 0.000109 | -4.5601 | Down | PHD finger protein 3 |
| ILMN_1781560 | ST3GAL6 | -0.8137 | 0.000109 | 0.000109 | -4.5601 | Down | ST3 beta-galactoside alpha-2,3-sialyltransferase 6 |
| ILMN_1655422 | RPL17 | -0.52633 | 0.000122 | 0.000122 | -4.51945 | Down | ribosomal protein L17 |
| ILMN_1795228 | ZFAND5 | -0.72934 | 0.000127 | 0.000127 | -4.50466 | Down | zinc finger AN1-type containing 5 |
| ILMN_1690386 | KAT14 | -0.43942 | 0.000133 | 0.000133 | -4.48521 | Down | lysine acetyltransferase 14 |
| ILMN_1736510 | FOXN2 | -0.49204 | 0.000133 | 0.000133 | -4.48497 | Down | forkhead box N2 |
| ILMN_1804798 | BEX4 | -0.6225 | 0.000143 | 0.000143 | -4.45813 | Down | brain expressed X-linked 4 |
| ILMN_1769277 | RPL9 | -0.41195 | 0.000148 | 0.000148 | -4.44428 | Down | ribosomal protein L9 |
| ILMN_1799642 | TRIM24 | -0.39819 | 0.000149 | 0.000149 | -4.44385 | Down | tripartite motif containing 24 |
| ILMN_2294976 | ANG | -0.79076 | 0.000159 | 0.000159 | -4.41709 | Down | angiogenin |
| ILMN_1805996 | SIN3A | -0.56259 | 0.00016 | 0.00016 | -4.41525 | Down | SIN3 transcription regulator family member A |
| ILMN_2175075 | SRSF4 | -0.44136 | 0.000163 | 0.000163 | -4.40778 | Down | serine and arginine rich splicing factor 4 |
| ILMN_1793616 | RNF38 | -0.44936 | 0.000167 | 0.000167 | -4.39862 | Down | ring finger protein 38 |
| ILMN_1677962 | GPHN | -0.65151 | 0.000168 | 0.000168 | -4.39736 | Down | gephyrin |
| ILMN_1902658 | LINC01278 | -0.40063 | 0.000179 | 0.000179 | -4.37307 | Down | long intergenic non-protein coding RNA 1278 |
| ILMN_2401258 | FAM13A | -0.9418 | 0.000181 | 0.000181 | -4.36775 | Down | family with sequence similarity 13 member A |
| ILMN_1678504 | RHOT1 | -0.57537 | 0.000189 | 0.000189 | -4.35223 | Down | ras homolog family member T1 |
| ILMN_1696974 | ANG | -0.71006 | 0.000199 | 0.000199 | -4.33318 | Down | angiogenin |
| ILMN_2124951 | RBMX | -0.5553 | 0.000203 | 0.000203 | -4.32423 | Down | RNA binding motif protein X-linked |
| ILMN_1720745 | HNRNPA1P58 | -0.72079 | 0.000205 | 0.000205 | -4.32093 | Down | heterogeneous nuclear ribonucleoprotein A1 pseudogene 58 |
| ILMN_1660986 | PER3 | -0.81664 | 0.000205 | 0.000205 | -4.32037 | Down | period circadian regulator 3 |
| ILMN_1682864 | SPSB3 | -0.45379 | 0.000222 | 0.000222 | -4.29046 | Down | splA/ryanodine receptor domain and SOCS box containing 3 |
| ILMN_1723212 | SRSF3 | -0.50378 | 0.000228 | 0.000228 | -4.28117 | Down | serine and arginine rich splicing factor 3 |
| ILMN_1707965 | ZFHX4-AS1 | -0.58402 | 0.000228 | 0.000228 | -4.28102 | Down | ZFHX4 antisense RNA 1 |
| ILMN_1695276 | MAPRE2 | -0.63141 | 0.00024 | 0.00024 | -4.26121 | Down | microtubule associated protein RP/EB family member 2 |
| ILMN_1809139 | AHCTF1 | -0.5111 | 0.000242 | 0.000242 | -4.25724 | Down | AT-hook containing transcription factor 1 |
| ILMN_1795678 | POLR3C | -0.43557 | 0.000251 | 0.000251 | -4.24422 | Down | RNA polymerase III subunit C |
| ILMN_1663489 | UBR2 | -0.39554 | 0.000253 | 0.000253 | -4.24027 | Down | ubiquitin protein ligase E3 component n-recognin 2 |
| ILMN_1734317 | DPF2 | -0.5493 | 0.000256 | 0.000256 | -4.2369 | Down | double PHD fingers 2 |
| ILMN_1747192 | RNF125 | -0.39931 | 0.000256 | 0.000256 | -4.23562 | Down | ring finger protein 125 |
| ILMN_1752510 | FAM13A | -0.99837 | 0.000257 | 0.000257 | -4.23415 | Down | family with sequence similarity 13 member A |
| ILMN_2394777 | DTNA | -0.61549 | 0.000264 | 0.000264 | -4.22394 | Down | dystrobrevin alpha |
| ILMN_2157219 | AASS | -0.55808 | 0.000265 | 0.000265 | -4.2237 | Down | aminoadipate-semialdehyde synthase |
| ILMN_2042595 | PCM1 | -0.40898 | 0.000265 | 0.000265 | -4.2224 | Down | pericentriolar material 1 |
| ILMN_1740024 | NAALAD2 | -1.17474 | 0.000266 | 0.000266 | -4.2222 | Down | N-acetylated alpha-linked acidic dipeptidase 2 |
| ILMN_1682316 | TRIM33 | -0.40627 | 0.000266 | 0.000266 | -4.22211 | Down | tripartite motif containing 33 |
| ILMN_1734897 | SLC4A4 | -0.62874 | 0.000268 | 0.000268 | -4.21926 | Down | solute carrier family 4 member 4 |
| ILMN_2276952 | TSC22D3 | -0.78215 | 0.000272 | 0.000272 | -4.21282 | Down | TSC22 domain family member 3 |
| ILMN_2294978 | ANG | -0.79321 | 0.000276 | 0.000276 | -4.20706 | Down | angiogenin |
| ILMN_2080751 | ADNP2 | -0.4253 | 0.00028 | 0.00028 | -4.20216 | Down | ADNP homeobox 2 |
| ILMN_1735979 | BCKDHA | -0.54967 | 0.000283 | 0.000283 | -4.19813 | Down | branched chain keto acid dehydrogenase E1, alpha polypeptide |
| ILMN_1761560 | PHF13 | -0.58385 | 0.000285 | 0.000285 | -4.1947 | Down | PHD finger protein 13 |
| ILMN_1810436 | DNAJC27 | -0.40591 | 0.000295 | 0.000295 | -4.18259 | Down | DnaJ heat shock protein family (Hsp40) member C27 |
| ILMN_1690586 | HNRNPA1P4 | -0.57304 | 0.000296 | 0.000296 | -4.18026 | Down | heterogeneous nuclear ribonucleoprotein A1 pseudogene 4 |
| ILMN_1683660 | EIF3H | -0.40346 | 0.000309 | 0.000309 | -4.16481 | Down | eukaryotic translation initiation factor 3 subunit H |
| ILMN_2327795 | RERE | -0.42563 | 0.000312 | 0.000312 | -4.16001 | Down | arginine-glutamic acid dipeptide repeats |
| ILMN_1802096 | ABTB1 | -0.52379 | 0.000328 | 0.000328 | -4.14094 | Down | ankyrin repeat and BTB domain containing 1 |
| ILMN_2325978 | HDGFL2 | -0.53491 | 0.00034 | 0.00034 | -4.12766 | Down | HDGF like 2 |
| ILMN_1746929 | DNAH17 | -0.47091 | 0.000342 | 0.000342 | -4.12492 | Down | dynein axonemal heavy chain 17 |
| ILMN_2348975 | NASP | -0.40415 | 0.000344 | 0.000344 | -4.12336 | Down | nuclear autoantigenic sperm protein |
| ILMN_2207539 | RPS17 | -0.42621 | 0.000344 | 0.000344 | -4.12328 | Down | ribosomal protein S17 |
| ILMN_1727633 | NECTIN3 | -0.40189 | 0.000351 | 0.000351 | -4.11545 | Down | nectin cell adhesion molecule 3 |
| ILMN_1768575 | SFTPD | -0.72081 | 0.000351 | 0.000351 | -4.11531 | Down | surfactant protein D |
| ILMN_1795856 | ELF2 | -0.47007 | 0.000357 | 0.000357 | -4.10893 | Down | E74 like ETS transcription factor 2 |
| ILMN_1756417 | ANKRD37 | -0.4384 | 0.000361 | 0.000361 | -4.10435 | Down | ankyrin repeat domain 37 |
| ILMN_1767422 | POLR1D | -0.54704 | 0.000365 | 0.000365 | -4.10011 | Down | RNA polymerase I and III subunit D |
| ILMN_1707339 | BTG3 | -0.44002 | 0.000377 | 0.000377 | -4.08847 | Down | BTG anti-proliferation factor 3 |
| ILMN_1800461 | CSNK2B | -0.39465 | 0.000385 | 0.000385 | -4.08031 | Down | casein kinase 2 beta |
| ILMN_1654060 | MKNK2 | -0.51063 | 0.000401 | 0.000401 | -4.06434 | Down | MAP kinase interacting serine/threonine kinase 2 |
| ILMN_1671603 | MED30 | -0.42207 | 0.000408 | 0.000408 | -4.05791 | Down | mediator complex subunit 30 |
| ILMN_1652846 | PCYT2 | -0.58494 | 0.000424 | 0.000424 | -4.04302 | Down | phosphate cytidylyltransferase 2, ethanolamine |
| ILMN_1672743 | ZNF334 | -0.44795 | 0.000426 | 0.000426 | -4.04083 | Down | zinc finger protein 334 |
| ILMN_1789809 | RPL13AP6 | -0.5148 | 0.000429 | 0.000429 | -4.03806 | Down | ribosomal protein L13a pseudogene 6 |
| ILMN_1740960 | MACROD1 | -0.48775 | 0.000432 | 0.000432 | -4.03593 | Down | MACRO domain containing 1 |
| ILMN_1652409 | SPATA7 | -0.59601 | 0.000433 | 0.000433 | -4.03481 | Down | spermatogenesis associated 7 |
| ILMN_1741881 | C9orf72 | -0.57025 | 0.000471 | 0.000471 | -4.00247 | Down | chromosome 9 open reading frame 72 |
| ILMN_1699644 | MARCH3 | -0.5546 | 0.000472 | 0.000472 | -4.00193 | Down | membrane associated ring-CH-type finger 3 |
| ILMN_1714170 | SPSB1 | -0.58359 | 0.000477 | 0.000477 | -3.99765 | Down | splA/ryanodine receptor domain and SOCS box containing 1 |
| ILMN_1655796 | MARCH3 | -0.61363 | 0.000496 | 0.000496 | -3.98239 | Down | membrane associated ring-CH-type finger 3 |
| ILMN_1711069 | YPEL5 | -0.40328 | 0.000501 | 0.000501 | -3.97844 | Down | yippee like 5 |
| ILMN_2194627 | GMCL1 | -0.68208 | 0.000505 | 0.000505 | -3.97585 | Down | germ cell-less, spermatogenesis associated 1 |
| ILMN_2110167 | POLR1E | -0.39568 | 0.000531 | 0.000531 | -3.95636 | Down | RNA polymerase I subunit E |
| ILMN_2305112 | CTH | -0.79808 | 0.000544 | 0.000544 | -3.94675 | Down | cystathionine gamma-lyase |
| ILMN_2182148 | CNRIP1 | -0.71975 | 0.000557 | 0.000557 | -3.93763 | Down | cannabinoid receptor interacting protein 1 |
| ILMN_1793410 | SNTB1 | -0.48614 | 0.000561 | 0.000561 | -3.9354 | Down | syntrophin beta 1 |
| ILMN_1708098 | LIX1L | -0.48995 | 0.000563 | 0.000563 | -3.93347 | Down | limb and CNS expressed 1 like |
| ILMN_1780132 | PELI2 | -0.68127 | 0.00058 | 0.00058 | -3.92231 | Down | pellino E3 ubiquitin protein ligase family member 2 |
| ILMN_1702821 | TTLL7 | -0.41216 | 0.000586 | 0.000586 | -3.91858 | Down | tubulin tyrosine ligase like 7 |
| ILMN_1723843 | CSNK2A2 | -0.47468 | 0.000595 | 0.000595 | -3.91234 | Down | casein kinase 2 alpha 2 |
| ILMN_1801441 | RFTN2 | -0.62656 | 0.000596 | 0.000596 | -3.91203 | Down | raftlin family member 2 |
| ILMN_2311278 | ADD3 | -0.52845 | 0.000603 | 0.000603 | -3.9073 | Down | adducin 3 |
| ILMN_1729142 | CENPV | -0.58292 | 0.000624 | 0.000624 | -3.89406 | Down | centromere protein V |
| ILMN_2038772 | RPS9 | -0.51676 | 0.00063 | 0.00063 | -3.89034 | Down | ribosomal protein S9 |
| ILMN_1722059 | SAFB | -0.45526 | 0.000656 | 0.000656 | -3.87472 | Down | scaffold attachment factor B |
| ILMN_1656682 | AZIN1 | -0.42329 | 0.000656 | 0.000656 | -3.87459 | Down | antizyme inhibitor 1 |
| ILMN_1794875 | GPAT3 | -1.48566 | 0.000664 | 0.000664 | -3.87013 | Down | glycerol-3-phosphate acyltransferase 3 |
| ILMN_1717420 | UBE2W | -0.51123 | 0.000689 | 0.000689 | -3.8557 | Down | ubiquitin conjugating enzyme E2 W |
| ILMN_1662318 | CCDC59 | -0.44764 | 0.000697 | 0.000697 | -3.85146 | Down | coiled-coil domain containing 59 |
| ILMN_1786242 | RPL14 | -0.39564 | 0.000698 | 0.000698 | -3.85088 | Down | ribosomal protein L14 |
| ILMN_1814661 | PHLPP1 | -0.45914 | 0.000699 | 0.000699 | -3.84995 | Down | PH domain and leucine rich repeat protein phosphatase 1 |
| ILMN_1665132 | CD36 | -0.92302 | 0.000708 | 0.000708 | -3.84519 | Down | CD36 molecule |
| ILMN_1685357 | RTN2 | -0.46378 | 0.000709 | 0.000709 | -3.84451 | Down | reticulon 2 |
| ILMN_1670532 | GMCL1 | -0.63244 | 0.000711 | 0.000711 | -3.84355 | Down | germ cell-less, spermatogenesis associated 1 |
| ILMN_2376403 | TSC22D3 | -0.93577 | 0.000745 | 0.000745 | -3.82526 | Down | TSC22 domain family member 3 |
| ILMN_1771734 | GMPS | -0.40677 | 0.000751 | 0.000751 | -3.82208 | Down | guanine monophosphate synthase |
| ILMN_1777660 | RNF144A | -0.66466 | 0.000765 | 0.000765 | -3.81523 | Down | ring finger protein 144A |
| ILMN_1677228 | TMLHE | -0.41087 | 0.000781 | 0.000781 | -3.80705 | Down | trimethyllysine hydroxylase, epsilon |
| ILMN_1683447 | RPL35 | -0.40252 | 0.000798 | 0.000798 | -3.7986 | Down | ribosomal protein L35 |
| ILMN_1749081 | AUTS2 | -0.61612 | 0.000799 | 0.000799 | -3.79821 | Down | AUTS2, activator of transcription and developmental regulator |
| ILMN_1679949 | SLC25A23 | -0.55426 | 0.0008 | 0.0008 | -3.79792 | Down | solute carrier family 25 member 23 |
| ILMN_1655557 | INTS6 | -0.50525 | 0.000801 | 0.000801 | -3.79754 | Down | integrator complex subunit 6 |
| ILMN_1797893 | N4BP2L2 | -0.43567 | 0.000802 | 0.000802 | -3.79699 | Down | NEDD4 binding protein 2 like 2 |
| ILMN_1746819 | C5 | -0.6768 | 0.000805 | 0.000805 | -3.79521 | Down | complement C5 |
| ILMN_1683575 | TMLHE | -0.49273 | 0.000814 | 0.000814 | -3.79109 | Down | trimethyllysine hydroxylase, epsilon |
| ILMN_1792885 | CTSC | -0.91588 | 0.000842 | 0.000842 | -3.77782 | Down | cathepsin C |
| ILMN_1804679 | KAT8 | -0.45615 | 0.000842 | 0.000842 | -3.77777 | Down | lysine acetyltransferase 8 |
| ILMN_1713682 | FBXO11 | -0.39613 | 0.000844 | 0.000844 | -3.7772 | Down | F-box protein 11 |
| ILMN_1695110 | BCAT2 | -0.55233 | 0.000881 | 0.000881 | -3.76017 | Down | branched chain amino acid transaminase 2 |
| ILMN_1791097 | RSBN1 | -0.43263 | 0.000887 | 0.000887 | -3.75766 | Down | round spermatid basic protein 1 |
| ILMN_1782543 | EEF1D | -0.44289 | 0.000891 | 0.000891 | -3.75596 | Down | eukaryotic translation elongation factor 1 delta |
| ILMN_1757338 | PLSCR4 | -0.48847 | 0.000891 | 0.000891 | -3.75577 | Down | phospholipid scramblase 4 |
| ILMN_1701134 | PTEN | -0.5057 | 0.000927 | 0.000927 | -3.74036 | Down | phosphatase and tensin homolog |
| ILMN_2228732 | CCNG2 | -0.68275 | 0.000933 | 0.000933 | -3.7381 | Down | cyclin G2 |
| ILMN_1713764 | #N/A | -0.58118 | 0.000935 | 0.000935 | -3.73719 | Down | NA |
| ILMN_1796962 | PPP3R1 | -0.45421 | 0.000939 | 0.000939 | -3.7357 | Down | protein phosphatase 3 regulatory subunit B, alpha |
| ILMN_1700316 | RPS12 | -0.51024 | 0.000943 | 0.000943 | -3.73387 | Down | ribosomal protein S12 |
| ILMN_1756999 | RBL2 | -0.42764 | 0.000953 | 0.000953 | -3.72994 | Down | RB transcriptional corepressor like 2 |
| ILMN_1697510 | ACSS2 | -0.43839 | 0.000958 | 0.000958 | -3.72791 | Down | acyl-CoA synthetase short chain family member 2 |
| ILMN_1697448 | TXNIP | -0.41817 | 0.000968 | 0.000968 | -3.72371 | Down | thioredoxin interacting protein |
| ILMN_1681703 | FOXO3 | -0.40924 | 0.00097 | 0.00097 | -3.72296 | Down | forkhead box O3 |
| ILMN_2347193 | GSDMB | -0.87834 | 0.00097 | 0.00097 | -3.72284 | Down | gasdermin B |
| ILMN_1666739 | RBM15 | -0.39717 | 0.000973 | 0.000973 | -3.72161 | Down | RNA binding motif protein 15 |
| ILMN_1778836 | SRSF7 | -0.63062 | 0.000988 | 0.000988 | -3.71565 | Down | serine and arginine rich splicing factor 7 |
| ILMN_1732074 | #N/A | -0.74016 | 0.001026 | 0.001026 | -3.70082 | Down | NA |
| ILMN_1661173 | TRIP4 | -0.40095 | 0.001026 | 0.001026 | -3.70082 | Down | thyroid hormone receptor interactor 4 |
| ILMN_1681634 | PXMP2 | -0.83494 | 0.001028 | 0.001028 | -3.70021 | Down | peroxisomal membrane protein 2 |
| ILMN_1733305 | EIF2A | -0.41666 | 0.001029 | 0.001029 | -3.69992 | Down | eukaryotic translation initiation factor 2A |
| ILMN_2318638 | TGIF1 | -0.45231 | 0.001031 | 0.001031 | -3.69897 | Down | TGFB induced factor homeobox 1 |
| ILMN_1660847 | PFKFB3 | -0.98614 | 0.001042 | 0.001042 | -3.69509 | Down | 6-phosphofructo-2-kinase/fructose-2,6-biphosphatase 3 |
| ILMN_1767142 | ZNF280D | -0.6049 | 0.001066 | 0.001066 | -3.68608 | Down | zinc finger protein 280D |
| ILMN_2401253 | FAM13A | -0.67261 | 0.001092 | 0.001092 | -3.67663 | Down | family with sequence similarity 13 member A |
| ILMN_1677607 | SC5D | -0.81449 | 0.001095 | 0.001095 | -3.67563 | Down | sterol-C5-desaturase |
| ILMN_1810864 | PMP22 | -0.63497 | 0.001096 | 0.001096 | -3.67519 | Down | peripheral myelin protein 22 |
| ILMN_1709132 | ELP2 | -0.43637 | 0.001107 | 0.001107 | -3.6712 | Down | elongatoracetyltransferase complex subunit 2 |
| ILMN_1726250 | CPAMD8 | -0.45219 | 0.001127 | 0.001127 | -3.6644 | Down | C3 and PZP like, alpha-2-macroglobulin domain containing 8 |
| ILMN_2278433 | LOC285074 | -0.54057 | 0.001137 | 0.001137 | -3.66096 | Down | anaphase promoting complex subunit 1 pseudogene |
| ILMN_1719205 | FBL | -0.43794 | 0.001168 | 0.001168 | -3.65035 | Down | fibrillarin |
| ILMN_1751785 | DMRT2 | -0.75395 | 0.001185 | 0.001185 | -3.64476 | Down | doublesex and mab-3 related transcription factor 2 |
| ILMN_1760714 | RPS3 | -0.44835 | 0.001187 | 0.001187 | -3.64418 | Down | ribosomal protein S3 |
| ILMN_2264681 | LETMD1 | -0.51143 | 0.001207 | 0.001207 | -3.63751 | Down | LETM1 domain containing 1 |
| ILMN_1789171 | EEF2K | -0.63414 | 0.00123 | 0.00123 | -3.63021 | Down | eukaryotic elongation factor 2 kinase |
| ILMN_1704537 | PHGDH | -1.18282 | 0.001241 | 0.001241 | -3.62676 | Down | phosphoglycerate dehydrogenase |
| ILMN_2146761 | FABP5 | -0.51494 | 0.001249 | 0.001249 | -3.62413 | Down | fatty acid binding protein 5 |
| ILMN_1657373 | P3H2 | -0.6377 | 0.001252 | 0.001252 | -3.62331 | Down | prolyl 3-hydroxylase 2 |
| ILMN_1695598 | RPL18 | -0.46798 | 0.001256 | 0.001256 | -3.62204 | Down | ribosomal protein L18 |
| ILMN_1773964 | H1FX | -0.41349 | 0.00127 | 0.00127 | -3.61761 | Down | H1 histone family member X |
| ILMN_1748124 | TSC22D3 | -0.77823 | 0.001316 | 0.001316 | -3.60369 | Down | TSC22 domain family member 3 |
| ILMN_1769319 | CNBP | -0.47448 | 0.001332 | 0.001332 | -3.59896 | Down | CCHC-type zinc finger nucleic acid binding protein |
| ILMN_1703565 | NOP53 | -0.42352 | 0.001341 | 0.001341 | -3.59639 | Down | NOP53 ribosome biogenesis factor |
| ILMN_1772876 | ZNF395 | -0.44848 | 0.00136 | 0.00136 | -3.59063 | Down | zinc finger protein 395 |
| ILMN_2351638 | BEX4 | -0.53061 | 0.001378 | 0.001378 | -3.5857 | Down | brain expressed X-linked 4 |
| ILMN_2338480 | RHOT1 | -0.58486 | 0.001382 | 0.001382 | -3.58439 | Down | ras homolog family member T1 |
| ILMN_1719696 | PLD1 | -0.47509 | 0.001387 | 0.001387 | -3.58296 | Down | phospholipase D1 |
| ILMN_2325168 | ARRB1 | -0.39681 | 0.001494 | 0.001494 | -3.5538 | Down | arrestin beta 1 |
| ILMN_1813530 | AGT | -0.5035 | 0.001525 | 0.001525 | -3.54573 | Down | angiotensinogen |
| ILMN_1799015 | PXMP2 | -0.84383 | 0.001573 | 0.001573 | -3.53362 | Down | peroxisomal membrane protein 2 |
| ILMN_2334042 | THYN1 | -0.57793 | 0.001587 | 0.001587 | -3.53007 | Down | thymocyte nuclear protein 1 |
| ILMN_1744347 | AL590609.1 | -0.45678 | 0.001595 | 0.001595 | -3.52796 | Down | ribosomal protein L36 (RPL36) pseudogene |
| ILMN_1657515 | RPS6KA5 | -0.52375 | 0.001607 | 0.001607 | -3.52518 | Down | ribosomal protein S6 kinase A5 |
| ILMN_2054607 | CYP4V2 | -0.59585 | 0.001635 | 0.001635 | -3.51834 | Down | cytochrome P450 family 4 subfamily V member 2 |
| ILMN_2323801 | MOCS1 | -0.7044 | 0.001651 | 0.001651 | -3.51439 | Down | molybdenum cofactor synthesis 1 |
| ILMN_1695034 | HNRNPA1P37 | -0.71138 | 0.001653 | 0.001653 | -3.51391 | Down | heterogeneous nuclear ribonucleoprotein A1 pseudogene 37 |
| ILMN_1666206 | GSDMB | -1.03517 | 0.001664 | 0.001664 | -3.51145 | Down | gasdermin B |
| ILMN_1749834 | SMIM1 | -0.53971 | 0.001697 | 0.001697 | -3.50354 | Down | small integral membrane protein 1 (Vel blood group) |
| ILMN_1698934 | CMTM7 | -0.59983 | 0.001701 | 0.001701 | -3.5028 | Down | CKLF like MARVEL transmembrane domain containing 7 |
| ILMN_1754149 | LETMD1 | -0.40275 | 0.001741 | 0.001741 | -3.4934 | Down | LETM1 domain containing 1 |
| ILMN_1710885 | RPSAP12 | -0.39686 | 0.001827 | 0.001827 | -3.47443 | Down | ribosomal protein SA pseudogene 12 |
| ILMN_1788689 | PHIP | -0.52812 | 0.001853 | 0.001853 | -3.46884 | Down | pleckstrin homology domain interacting protein |
| ILMN_2407346 | LDHD | -0.78788 | 0.001859 | 0.001859 | -3.46766 | Down | lactate dehydrogenase D |
| ILMN_1654289 | ELK1 | -0.4093 | 0.001901 | 0.001901 | -3.45883 | Down | ELK1, ETS transcription factor |
| ILMN_1719661 | MSRB1 | -0.42174 | 0.001905 | 0.001905 | -3.45788 | Down | methionine sulfoxidereductase B1 |
| ILMN_1676091 | HNRNPA1P7 | -0.58631 | 0.001975 | 0.001975 | -3.44365 | Down | heterogeneous nuclear ribonucleoprotein A1 pseudogene 7 |
| ILMN_1765500 | NDUFV3 | -0.47187 | 0.001982 | 0.001982 | -3.44214 | Down | NADH:ubiquinoneoxidoreductase subunit V3 |
| ILMN_1765032 | MUC20-OT1 | -0.54689 | 0.002004 | 0.002004 | -3.4379 | Down | MUC20 overlapping transcript |
| ILMN_1702363 | SULF1 | -1.08556 | 0.002014 | 0.002014 | -3.43577 | Down | sulfatase 1 |
| ILMN_1743620 | RARRES1 | -0.52985 | 0.002029 | 0.002029 | -3.43282 | Down | retinoic acid receptor responder 1 |
| ILMN_1657760 | SYT17 | -0.47881 | 0.002082 | 0.002082 | -3.42268 | Down | synaptotagmin 17 |
| ILMN_1655163 | STK24 | -0.52056 | 0.002104 | 0.002104 | -3.4184 | Down | serine/threonine kinase 24 |
| ILMN_1722089 | RNF217 | -0.49485 | 0.002117 | 0.002117 | -3.41611 | Down | ring finger protein 217 |
| ILMN_2402817 | ZBTB16 | -0.77388 | 0.002137 | 0.002137 | -3.41224 | Down | zinc finger and BTB domain containing 16 |
| ILMN_1783956 | ATP8B4 | -0.6446 | 0.00214 | 0.00214 | -3.41172 | Down | ATPase phospholipid transporting 8B4 (putative) |
| ILMN_2183938 | LEMD3 | -0.53026 | 0.002156 | 0.002156 | -3.40882 | Down | LEM domain containing 3 |
| ILMN_1698685 | MATN2 | -0.40191 | 0.002157 | 0.002157 | -3.40864 | Down | matrilin 2 |
| ILMN_1654690 | HDHD5 | -0.39979 | 0.00217 | 0.00217 | -3.40619 | Down | haloaciddehalogenase like hydrolase domain containing 5 |
| ILMN_1700831 | SLC27A2 | -1.11722 | 0.002174 | 0.002174 | -3.40554 | Down | solute carrier family 27 member 2 |
| ILMN_1679041 | SLC3A2 | -0.49613 | 0.002178 | 0.002178 | -3.40467 | Down | solute carrier family 3 member 2 |
| ILMN_1776363 | ANK2 | -0.6836 | 0.002187 | 0.002187 | -3.40315 | Down | ankyrin 2 |
| ILMN_1786197 | NR2F1 | -0.50821 | 0.00219 | 0.00219 | -3.40251 | Down | nuclear receptor subfamily 2 group F member 1 |
| ILMN_1678323 | AASS | -0.51408 | 0.002208 | 0.002208 | -3.39938 | Down | aminoadipate-semialdehyde synthase |
| ILMN_1657810 | PPM1M | -0.50248 | 0.002231 | 0.002231 | -3.39512 | Down | protein phosphatase, Mg2+/Mn2+ dependent 1M |
| ILMN_1695590 | ADRB2 | -0.86384 | 0.002236 | 0.002236 | -3.39424 | Down | adrenoceptor beta 2 |
| ILMN_1807554 | EYA1 | -0.45309 | 0.00224 | 0.00224 | -3.39358 | Down | EYA transcriptional coactivator and phosphatase 1 |
| ILMN_1844692 | FOXO3 | -0.4053 | 0.002262 | 0.002262 | -3.38971 | Down | forkhead box O3 |
| ILMN_1701947 | GPR34 | -0.63372 | 0.002273 | 0.002273 | -3.38773 | Down | G protein-coupled receptor 34 |
| ILMN_1765208 | GLUL | -0.48308 | 0.002351 | 0.002351 | -3.37425 | Down | glutamate-ammonia ligase |
| ILMN_1747244 | CCNG2 | -0.46567 | 0.002393 | 0.002393 | -3.36729 | Down | cyclin G2 |
| ILMN_1798624 | MOCS1 | -0.55467 | 0.002445 | 0.002445 | -3.35871 | Down | molybdenum cofactor synthesis 1 |
| ILMN_1680196 | LAPTM4B | -0.44371 | 0.002502 | 0.002502 | -3.34953 | Down | lysosomal protein transmembrane 4 beta |
| ILMN_1737025 | PLCL2 | -0.47585 | 0.002545 | 0.002545 | -3.34262 | Down | phospholipase C like 2 |
| ILMN_1810433 | FAM207A | -0.40305 | 0.002599 | 0.002599 | -3.33437 | Down | family with sequence similarity 207 member A |
| ILMN_2083588 | TTC32 | -0.40982 | 0.002602 | 0.002602 | -3.33388 | Down | tetratricopeptide repeat domain 32 |
| ILMN_1880406 | PTEN | -0.44058 | 0.002689 | 0.002689 | -3.32067 | Down | phosphatase and tensin homolog |
| ILMN_2348367 | FGFRL1 | -0.62279 | 0.002736 | 0.002736 | -3.31382 | Down | fibroblast growth factor receptor like 1 |
| ILMN_1683263 | TSPAN8 | -0.61312 | 0.002774 | 0.002774 | -3.30821 | Down | tetraspanin 8 |
| ILMN_1742788 | PKD1L2 | -1.01131 | 0.002779 | 0.002779 | -3.30747 | Down | polycystin 1 like 2 (gene/pseudogene) |
| ILMN_1780236 | PMM1 | -0.63809 | 0.00282 | 0.00282 | -3.30165 | Down | phosphomannomutase 1 |
| ILMN_1791467 | PLIN5 | -0.41569 | 0.002865 | 0.002865 | -3.29524 | Down | perilipin 5 |
| ILMN_2290808 | RPL21 | -0.59943 | 0.002867 | 0.002867 | -3.29498 | Down | ribosomal protein L21 |
| ILMN_1710124 | CMTM8 | -0.80058 | 0.002943 | 0.002943 | -3.28454 | Down | CKLF like MARVEL transmembrane domain containing 8 |
| ILMN_1779486 | FAM126B | -0.47129 | 0.002978 | 0.002978 | -3.2797 | Down | family with sequence similarity 126 member B |
| ILMN_2255310 | RPS15A | -0.63234 | 0.002987 | 0.002987 | -3.27853 | Down | ribosomal protein S15a |
| ILMN_1653496 | GLUL | -0.72238 | 0.00305 | 0.00305 | -3.27012 | Down | glutamate-ammonia ligase |
| ILMN_1758281 | CALCRL | -0.78586 | 0.003177 | 0.003177 | -3.25369 | Down | calcitonin receptor like receptor |
| ILMN_2049303 | ECI1 | -0.55796 | 0.003187 | 0.003187 | -3.25251 | Down | enoyl-CoA delta isomerase 1 |
| ILMN_1854349 | RNF217 | -0.51519 | 0.003187 | 0.003187 | -3.25248 | Down | ring finger protein 217 |
| ILMN_1798654 | MCM6 | -0.45891 | 0.003246 | 0.003246 | -3.2451 | Down | minichromosome maintenance complex component 6 |
| ILMN_1781638 | HDDC3 | -0.42977 | 0.003264 | 0.003264 | -3.2429 | Down | HD domain containing 3 |
| ILMN_1753534 | RPS18 | -0.4732 | 0.003407 | 0.003407 | -3.22559 | Down | ribosomal protein S18 |
| ILMN_1692177 | TSC22D1 | -0.54834 | 0.003461 | 0.003461 | -3.21918 | Down | TSC22 domain family member 1 |
| ILMN_1801795 | RPL18A | -0.47586 | 0.003522 | 0.003522 | -3.21216 | Down | ribosomal protein L18a |
| ILMN_1715024 | LSS | -0.48264 | 0.003722 | 0.003722 | -3.18981 | Down | lanosterol synthase |
| ILMN_1761322 | FHOD3 | -0.85631 | 0.003793 | 0.003793 | -3.18216 | Down | formin homology 2 domain containing 3 |
| ILMN_1779751 | FMC1 | -0.45529 | 0.003804 | 0.003804 | -3.18097 | Down | formation of mitochondrial complex V assembly factor 1 homolog |
| ILMN_1785284 | ALDH6A1 | -0.6169 | 0.003926 | 0.003926 | -3.16823 | Down | aldehyde dehydrogenase 6 family member A1 |
| ILMN_1660832 | RPL18A | -0.42124 | 0.003963 | 0.003963 | -3.16437 | Down | ribosomal protein L18a |
| ILMN_1653001 | CABLES1 | -0.40265 | 0.004025 | 0.004025 | -3.15807 | Down | Cdk5 and Abl enzyme substrate 1 |
| ILMN_2384807 | LRRCC1 | -0.44474 | 0.004046 | 0.004046 | -3.15595 | Down | leucine rich repeat and coiled-coil centrosomal protein 1 |
| ILMN_1675460 | RPL29 | -0.39808 | 0.004048 | 0.004048 | -3.15575 | Down | ribosomal protein L29 |
| ILMN_1654609 | EPB41L4A-AS1 | -0.51803 | 0.004077 | 0.004077 | -3.15286 | Down | EPB41L4A antisense RNA 1 |
| ILMN_1787248 | SIVA1 | -0.41798 | 0.004263 | 0.004263 | -3.13477 | Down | SIVA1 apoptosis inducing factor |
| ILMN_1773119 | CCNF | -0.43811 | 0.004344 | 0.004344 | -3.12704 | Down | cyclin F |
| ILMN_1740717 | ADH1C | -0.588 | 0.004462 | 0.004462 | -3.11609 | Down | alcohol dehydrogenase 1C (class I), gamma polypeptide |
| ILMN_2305407 | ZBTB16 | -0.68894 | 0.004508 | 0.004508 | -3.11199 | Down | zinc finger and BTB domain containing 16 |
| ILMN_1670134 | FADS1 | -1.09346 | 0.004556 | 0.004556 | -3.10763 | Down | fatty acid desaturase 1 |
| ILMN_1783684 | RBBP4 | -0.40939 | 0.00479 | 0.00479 | -3.08718 | Down | RB binding protein 4, chromatin remodeling factor |
| ILMN_1671478 | CKB | -0.63338 | 0.004827 | 0.004827 | -3.08404 | Down | creatine kinase B |
| ILMN_1755909 | GID8 | -0.40117 | 0.005017 | 0.005017 | -3.06817 | Down | GID complex subunit 8 homolog |
| ILMN_1813456 | PCBD1 | -0.64387 | 0.005022 | 0.005022 | -3.06778 | Down | pterin-4 alpha-carbinolaminedehydratase 1 |
| ILMN_1694840 | MATN2 | -0.49009 | 0.005145 | 0.005145 | -3.05787 | Down | matrilin 2 |
| ILMN_1692464 | TTC38 | -0.48834 | 0.005183 | 0.005183 | -3.05487 | Down | tetratricopeptide repeat domain 38 |
| ILMN_1664014 | STOX1 | -0.81909 | 0.005378 | 0.005378 | -3.03966 | Down | storkhead box 1 |
| ILMN_1731064 | COQ8A | -0.67616 | 0.005413 | 0.005413 | -3.03698 | Down | coenzyme Q8A |
| ILMN_1700733 | FAM214A | -0.45812 | 0.005537 | 0.005537 | -3.0277 | Down | family with sequence similarity 214 member A |
| ILMN_1662174 | ORMDL3 | -0.74365 | 0.00558 | 0.00558 | -3.02449 | Down | ORMDL sphingolipid biosynthesis regulator 3 |
| ILMN_1716797 | CD302 | -0.55642 | 0.005698 | 0.005698 | -3.01591 | Down | CD302 molecule |
| ILMN_2340131 | MAPK10 | -0.45954 | 0.005724 | 0.005724 | -3.014 | Down | mitogen-activated protein kinase 10 |
| ILMN_1718629 | NRIP1 | -0.63132 | 0.005777 | 0.005777 | -3.01023 | Down | nuclear receptor interacting protein 1 |
| ILMN_1757467 | H1F0 | -0.43213 | 0.00612 | 0.00612 | -2.98641 | Down | H1 histone family member 0 |
| ILMN_1773063 | OSBPL1A | -0.39462 | 0.006163 | 0.006163 | -2.98347 | Down | oxysterol binding protein like 1A |
| ILMN_2305116 | CTH | -0.39952 | 0.006295 | 0.006295 | -2.97474 | Down | cystathionine gamma-lyase |
| ILMN_1736911 | TMOD1 | -0.55465 | 0.006324 | 0.006324 | -2.97281 | Down | tropomodulin 1 |
| ILMN_1696347 | CTSC | -0.63671 | 0.006354 | 0.006354 | -2.97085 | Down | cathepsin C |
| ILMN_1835017 | GLUL | -0.52146 | 0.006647 | 0.006647 | -2.95216 | Down | glutamate-ammonia ligase |
| ILMN_1707062 | REV1 | -0.4136 | 0.006976 | 0.006976 | -2.93207 | Down | REV1, DNA directed polymerase |
| ILMN_1714384 | PCCA | -0.42758 | 0.007366 | 0.007366 | -2.90939 | Down | propionyl-CoA carboxylase subunit alpha |
| ILMN_1738207 | CISH | -0.82272 | 0.007368 | 0.007368 | -2.90929 | Down | cytokine inducible SH2 containing protein |
| ILMN_1783598 | CAB39L | -0.51195 | 0.00786 | 0.00786 | -2.88226 | Down | calcium binding protein 39 like |
| ILMN_1653494 | S100A1 | -0.75357 | 0.008027 | 0.008027 | -2.87347 | Down | S100 calcium binding protein A1 |
| ILMN_2218277 | RPS2 | -0.45343 | 0.008061 | 0.008061 | -2.87169 | Down | ribosomal protein S2 |
| ILMN_1754272 | GINS3 | -0.55926 | 0.008196 | 0.008196 | -2.86473 | Down | GINS complex subunit 3 |
| ILMN_1783149 | CDH23 | -0.49907 | 0.008324 | 0.008324 | -2.85821 | Down | cadherin related 23 |
| ILMN_1721580 | TBX15 | -0.52139 | 0.00849 | 0.00849 | -2.8499 | Down | T-box 15 |
| ILMN_1703123 | CSRNP1 | -0.48515 | 0.008494 | 0.008494 | -2.84975 | Down | cysteine and serine rich nuclear protein 1 |
| ILMN_1691966 | LINC02076 | -0.50304 | 0.008554 | 0.008554 | -2.84675 | Down | long intergenic non-protein coding RNA 2076 |
| ILMN_1695311 | HLA-DMA | -0.73553 | 0.00856 | 0.00856 | -2.84645 | Down | major histocompatibility complex, class II, DM alpha |
| ILMN_1750496 | ZBTB16 | -0.61415 | 0.008644 | 0.008644 | -2.84236 | Down | zinc finger and BTB domain containing 16 |
| ILMN_1739083 | SIRT1 | -0.43004 | 0.008765 | 0.008765 | -2.83651 | Down | sirtuin 1 |
| ILMN_1770085 | BTG2 | -0.62761 | 0.008768 | 0.008768 | -2.83636 | Down | BTG anti-proliferation factor 2 |
| ILMN_2401618 | MLX | -0.40216 | 0.008865 | 0.008865 | -2.83173 | Down | MLX, MAX dimerization protein |
| ILMN_2305225 | NDRG4 | -1.14352 | 0.008955 | 0.008955 | -2.82749 | Down | NDRG family member 4 |
| ILMN_1729033 | RPL9 | -0.44385 | 0.008985 | 0.008985 | -2.82605 | Down | ribosomal protein L9 |
| ILMN_1800091 | RARRES1 | -0.53546 | 0.00924 | 0.00924 | -2.81424 | Down | retinoic acid receptor responder 1 |
| ILMN_1791847 | DAPK2 | -0.52118 | 0.009391 | 0.009391 | -2.8074 | Down | death associated protein kinase 2 |
| ILMN_1676159 | STK26 | -0.62873 | 0.009423 | 0.009423 | -2.80595 | Down | serine/threonine kinase 26 |
| ILMN_1718977 | GADD45B | -0.59259 | 0.00965 | 0.00965 | -2.79591 | Down | growth arrest and DNA damage inducible beta |
| ILMN_1715555 | DBP | -0.53512 | 0.009791 | 0.009791 | -2.78974 | Down | D-box binding PAR bZIP transcription factor |
| ILMN_1698259 | TMEM100 | -0.77227 | 0.009942 | 0.009942 | -2.78326 | Down | transmembrane protein 100 |
| ILMN_1684368 | SMIM3 | -0.53983 | 0.010068 | 0.010068 | -2.7779 | Down | small integral membrane protein 3 |
| ILMN_1735367 | HSD17B11 | -0.42718 | 0.010203 | 0.010203 | -2.77228 | Down | hydroxysteroid 17-beta dehydrogenase 11 |
| ILMN_1679093 | ZNF581 | -0.4191 | 0.010916 | 0.010916 | -2.74352 | Down | zinc finger protein 581 |
| ILMN_1707649 | MPDZ | -0.44065 | 0.01123 | 0.01123 | -2.7314 | Down | multiple PDZ domain crumbs cell polarity complex component |
| ILMN_1671092 | SOX17 | -0.54376 | 0.011244 | 0.011244 | -2.73088 | Down | SRY-box 17 |
| ILMN_2328776 | STK26 | -0.49827 | 0.011334 | 0.011334 | -2.72746 | Down | serine/threonine kinase 26 |
| ILMN_1728083 | EIF4EBP2 | -0.4121 | 0.011407 | 0.011407 | -2.72471 | Down | eukaryotic translation initiation factor 4E binding protein 2 |
| ILMN_1709486 | SRPX | -0.59773 | 0.011581 | 0.011581 | -2.71826 | Down | sushi repeat containing protein X-linked |
| ILMN_1706660 | HYI | -0.47577 | 0.011645 | 0.011645 | -2.71588 | Down | hydroxypyruvateisomerase (putative) |
| ILMN_1738816 | FOXO1 | -0.50839 | 0.011736 | 0.011736 | -2.71257 | Down | forkhead box O1 |
| ILMN_1678535 | ESR1 | -0.48298 | 0.011741 | 0.011741 | -2.71236 | Down | estrogen receptor 1 |
| ILMN_1652246 | NACAD | -0.56561 | 0.012028 | 0.012028 | -2.702 | Down | NAC alpha domain containing |
| ILMN_1791222 | GLYCTK | -0.6914 | 0.012173 | 0.012173 | -2.69688 | Down | glycerate kinase |
| ILMN_1729749 | HERC5 | -0.74104 | 0.012635 | 0.012635 | -2.68089 | Down | HECT and RLD domain containing E3 ubiquitin protein ligase 5 |
| ILMN_1781819 | PAPSS1 | -0.41286 | 0.012711 | 0.012711 | -2.67828 | Down | 3'-phosphoadenosine 5'-phosphosulfate synthase 1 |
| ILMN_2229214 | STOX1 | -0.6532 | 0.01281 | 0.01281 | -2.67495 | Down | storkhead box 1 |
| ILMN_1900520 | #N/A | -0.47028 | 0.013101 | 0.013101 | -2.66528 | Down | NA |
| ILMN_1811933 | SHMT1 | -0.57643 | 0.013105 | 0.013105 | -2.66513 | Down | serine hydroxymethyltransferase 1 |
| ILMN_1700546 | ELOVL6 | -0.64894 | 0.013212 | 0.013212 | -2.66164 | Down | ELOVL fatty acid elongase 6 |
| ILMN_1814464 | RAMP2 | -0.44254 | 0.01341 | 0.01341 | -2.65519 | Down | receptor activity modifying protein 2 |
| ILMN_1781010 | ARHGEF3 | -0.67277 | 0.013631 | 0.013631 | -2.64813 | Down | Rho guanine nucleotide exchange factor 3 |
| ILMN_1778444 | FKBP5 | -1.05059 | 0.013648 | 0.013648 | -2.64759 | Down | FK506 binding protein 5 |
| ILMN_2220283 | HNRNPA1P10 | -0.47457 | 0.013952 | 0.013952 | -2.63806 | Down | heterogeneous nuclear ribonucleoprotein A1 pseudogene 10 |
| ILMN_1660635 | LACTB2 | -0.47503 | 0.014165 | 0.014165 | -2.63149 | Down | lactamase beta 2 |
| ILMN_1776213 | RGMB | -0.52099 | 0.014885 | 0.014885 | -2.60998 | Down | repulsive guidance molecule BMP co-receptor b |
| ILMN_2061452 | ORC2 | -0.47703 | 0.015166 | 0.015166 | -2.60185 | Down | origin recognition complex subunit 2 |
| ILMN_1743784 | SHMT1 | -0.47754 | 0.01575 | 0.01575 | -2.58537 | Down | serine hydroxymethyltransferase 1 |
| ILMN_1695299 | PDLIM3 | -0.42944 | 0.016438 | 0.016438 | -2.56668 | Down | PDZ and LIM domain 3 |
| ILMN_2131861 | SOCS2 | -0.64331 | 0.016571 | 0.016571 | -2.56315 | Down | suppressor of cytokine signaling 2 |
| ILMN_2242463 | CTSC | -0.54644 | 0.016581 | 0.016581 | -2.56289 | Down | cathepsin C |
| ILMN_1794914 | UBTD1 | -0.46864 | 0.01716 | 0.01716 | -2.54783 | Down | ubiquitin domain containing 1 |
| ILMN_1787567 | TSC22D1 | -0.41504 | 0.017272 | 0.017272 | -2.54497 | Down | TSC22 domain family member 1 |
| ILMN_2051972 | GPC3 | -0.47873 | 0.01777 | 0.01777 | -2.53246 | Down | glypican 3 |
| ILMN_1728799 | FBP1 | -0.43121 | 0.018124 | 0.018124 | -2.52375 | Down | fructose-bisphosphatase 1 |
| ILMN_1811598 | ADH1B | -0.64322 | 0.018502 | 0.018502 | -2.51465 | Down | alcohol dehydrogenase 1B (class I), beta polypeptide |
| ILMN_1690096 | PPP1R1B | -0.39748 | 0.018632 | 0.018632 | -2.51157 | Down | protein phosphatase 1 regulatory inhibitor subunit 1B |
| ILMN_1781256 | LEFTY2 | -0.42676 | 0.01877 | 0.01877 | -2.5083 | Down | left-right determination factor 2 |
| ILMN_1799280 | BDH1 | -0.43115 | 0.019319 | 0.019319 | -2.49554 | Down | 3-hydroxybutyrate dehydrogenase 1 |
| ILMN_1793543 | CIART | -0.50833 | 0.019494 | 0.019494 | -2.49156 | Down | circadian associated repressor of transcription |
| ILMN_1727087 | GJA1 | -0.62741 | 0.019563 | 0.019563 | -2.48998 | Down | gap junction protein alpha 1 |
| ILMN_1652955 | RPS27 | -0.51384 | 0.02033 | 0.02033 | -2.47289 | Down | ribosomal protein S27 |
| ILMN_1709613 | IGF1 | -0.5613 | 0.02055 | 0.02055 | -2.46811 | Down | insulin like growth factor 1 |
| ILMN_1702168 | HSD17B12 | -0.40602 | 0.021038 | 0.021038 | -2.45765 | Down | hydroxysteroid 17-beta dehydrogenase 12 |
| ILMN_1768534 | BHLHE40 | -0.47888 | 0.021039 | 0.021039 | -2.45763 | Down | basic helix-loop-helix family member e40 |
| ILMN_2230025 | PDLIM3 | -0.58435 | 0.022514 | 0.022514 | -2.42734 | Down | PDZ and LIM domain 3 |
| ILMN_1742917 | NXNL1 | -0.44529 | 0.022528 | 0.022528 | -2.42705 | Down | nucleoredoxin like 1 |
| ILMN_2331636 | ACACA | -0.54723 | 0.022716 | 0.022716 | -2.42333 | Down | acetyl-CoA carboxylase alpha |
| ILMN_1756928 | RTN1 | -0.52782 | 0.023225 | 0.023225 | -2.41338 | Down | reticulon 1 |
| ILMN_1725791 | HACD1 | -0.48778 | 0.023407 | 0.023407 | -2.40987 | Down | 3-hydroxyacyl-CoA dehydratase 1 |
| ILMN_1814526 | ADD3 | -0.54021 | 0.023431 | 0.023431 | -2.4094 | Down | adducin 3 |
| ILMN_2359345 | NET1 | -0.66813 | 0.023703 | 0.023703 | -2.40421 | Down | neuroepithelial cell transforming 1 |
| ILMN_2221006 | RAD21 | -0.67963 | 0.023956 | 0.023956 | -2.39942 | Down | RAD21 cohesin complex component |
| ILMN_1757882 | PPP1R16A | -0.52488 | 0.024408 | 0.024408 | -2.39099 | Down | protein phosphatase 1 regulatory subunit 16A |
| ILMN_2326324 | LDHC | -0.46033 | 0.024413 | 0.024413 | -2.39091 | Down | lactate dehydrogenase C |
| ILMN_1694780 | GCHFR | -0.57882 | 0.024843 | 0.024843 | -2.38302 | Down | GTP cyclohydrolase I feedback regulator |
| ILMN_2186061 | PFKFB3 | -0.53663 | 0.025098 | 0.025098 | -2.3784 | Down | 6-phosphofructo-2-kinase/fructose-2,6-biphosphatase 3 |
| ILMN_1694776 | SCP2 | -0.39618 | 0.025742 | 0.025742 | -2.36693 | Down | sterol carrier protein 2 |
| ILMN_1816634 | AC004160.1 | -0.47774 | 0.025816 | 0.025816 | -2.36563 | Down | novel transcript, antisense to THSD7A |
| ILMN_1763852 | ACACB | -0.42465 | 0.026406 | 0.026406 | -2.35538 | Down | acetyl-CoA carboxylase beta |
| ILMN_1749875 | AC141557.1 | -0.58299 | 0.026502 | 0.026502 | -2.35372 | Down | ovostatin (OVOS) pseudogene |
| ILMN_1772910 | GAS1 | -0.59307 | 0.027065 | 0.027065 | -2.34416 | Down | growth arrest specific 1 |
| ILMN_1701173 | KCNK6 | -0.465 | 0.027711 | 0.027711 | -2.33342 | Down | potassium two pore domain channel subfamily K member 6 |
| ILMN_1768505 | IL13RA1 | -0.39597 | 0.027731 | 0.027731 | -2.33309 | Down | interleukin 13 receptor subunit alpha 1 |
| ILMN_1772123 | ACACA | -0.5172 | 0.028001 | 0.028001 | -2.32866 | Down | acetyl-CoA carboxylase alpha |
| ILMN_1676765 | RPL18A | -0.45199 | 0.028068 | 0.028068 | -2.32756 | Down | ribosomal protein L18a |
| ILMN_1789558 | ZC2HC1A | -0.4103 | 0.028262 | 0.028262 | -2.32443 | Down | zinc finger C2HC-type containing 1A |
| ILMN_1743445 | FAM107A | -0.57785 | 0.028483 | 0.028483 | -2.32086 | Down | family with sequence similarity 107 member A |
| ILMN_1760574 | RAI2 | -0.44939 | 0.028548 | 0.028548 | -2.31981 | Down | retinoic acid induced 2 |
| ILMN_1800540 | CD55 | -0.49697 | 0.02916 | 0.02916 | -2.31011 | Down | CD55 molecule (Cromer blood group) |
| ILMN_1660890 | ACADL | -0.42123 | 0.02924 | 0.02924 | -2.30885 | Down | acyl-CoA dehydrogenase long chain |
| ILMN_1665510 | ERRFI1 | -0.49543 | 0.029318 | 0.029318 | -2.30764 | Down | ERBB receptor feedback inhibitor 1 |
| ILMN_1676822 | C2orf40 | -0.51359 | 0.029379 | 0.029379 | -2.30669 | Down | chromosome 2 open reading frame 40 |
| ILMN_1755808 | RPS27 | -0.59398 | 0.029664 | 0.029664 | -2.30225 | Down | ribosomal protein S27 |
| ILMN_1715401 | MT1G | -0.61901 | 0.029679 | 0.029679 | -2.30202 | Down | metallothionein 1G |
| ILMN_1782305 | NR4A2 | -0.66741 | 0.030329 | 0.030329 | -2.29206 | Down | nuclear receptor subfamily 4 group A member 2 |
| ILMN_1703102 | ST13P15 | -0.42368 | 0.030763 | 0.030763 | -2.28553 | Down | ST13, Hsp70 interacting protein pseudogene 15 |
| ILMN_2403237 | CHN2 | -0.42851 | 0.030784 | 0.030784 | -2.28522 | Down | chimerin 2 |
| ILMN_1797793 | BLVRB | -0.47037 | 0.031521 | 0.031521 | -2.27431 | Down | biliverdinreductase B |
| ILMN_2408572 | RNASE4 | -0.4736 | 0.035171 | 0.035171 | -2.22348 | Down | ribonuclease A family member 4 |
| ILMN_1684982 | PDK4 | -0.42956 | 0.038313 | 0.038313 | -2.18341 | Down | pyruvate dehydrogenase kinase 4 |
| ILMN_2371055 | EFNA1 | -0.49597 | 0.039014 | 0.039014 | -2.17488 | Down | ephrin A1 |
| ILMN_2267535 | NET1 | -0.48927 | 0.039057 | 0.039057 | -2.17437 | Down | neuroepithelial cell transforming 1 |
| ILMN_1776490 | C17orf53 | -0.39414 | 0.041169 | 0.041169 | -2.14949 | Down | chromosome 17 open reading frame 53 |
| ILMN_1652466 | WNT3 | -0.60148 | 0.045134 | 0.045134 | -2.10573 | Down | Wnt family member 3 |
| ILMN_1698554 | AACS | -0.50824 | 0.045299 | 0.045299 | -2.10398 | Down | acetoacetyl-CoA synthetase |
| ILMN_1722156 | RWDD2A | -0.42053 | 0.045756 | 0.045756 | -2.09917 | Down | RWD domain containing 2A |
| ILMN_2111187 | ELOVL6 | -0.7318 | 0.049464 | 0.049464 | -2.06169 | Down | ELOVL fatty acid elongase 6 |

**Table 3** The enriched pathway terms of the up-regulated differentially expressed genes

| **BIOCYC** | | | | |
| --- | --- | --- | --- | --- |
| **Pathway ID** | **Pathway Name** | **LogP** | **Gene Count** | **Genes** |
| 545361 | thyroid hormone metabolism II (via conjugation and/or degradation) | 2.643466898 | 02 | SULT1A1,SULT1A2 |
| 545353 | serotonin degradation | 2.25690676 | 02 | SULT1A1,SULT1A2 |
| 545356 | melatonin degradation I | 1.802432827 | 02 | SULT1A1,SULT1A2 |
| 545359 | superpathway of melatonin degradation | 1.720921143 | 02 | SULT1A1,SULT1A2 |
| 142378 | L-serine degradation | 1.695428398 | 01 | SDSL |
| 142400 | lactose degradation III | 1.695428398 | 01 | GLB1 |
| 835392 | superpathway of tryptophan utilization | 1.605692623 | 03 | QPRT,SULT1A1,SULT1A2 |
| 545334 | histamine degradation | 1.407410907 | 01 | HNMT |
| 547502 | glutamine degradation/glutamate biosynthesis | 1.407410907 | 01 | GLS |
| 545306 | dolichol and dolichyl phosphate biosynthesis | 1.407410907 | 01 | DHDDS |
| **KEGG** | | | | |
| 83068 | ECM-receptor interaction | 10 | 11 | COL1A2,COL6A1,COL6A2,DAG1,ITGAV,ITGB5,LAMA5,LAMB2,LAMB3,SPP1,THBS1 |
| 83067 | Focal adhesion | 10 | 18 | ACTN1,CCND1,CCND2,COL1A2,COL6A1,COL6A2,ERBB2,FLNC,ITGAV,ITGB5,LAMA5,LAMB2,LAMB3,MYL9,PDGFD,SOS1,SPP1,THBS1 |
| 692234 | PI3K-Akt signaling pathway | 4.828155946 | 20 | ANGPT1,CCND1,CCND2,COL1A2,COL6A1,COL6A2,FGF11,GNG2,ITGAV,ITGB5,LAMA5,LAMB2,LAMB3,PDGFD,PPP2R1B,SGK1,SOS1,SPP1,THBS1,YWHAH |
| 172847 | Protein digestion and absorption | 4.157653601 | 09 | COL11A1,COL12A1,COL15A1,COL1A2,COL3A1,COL5A2,COL6A1,COL6A2,ELN |
| 673221 | Chemical carcinogenesis | 3.67876368 | 08 | ALDH1A3,GSTM5,HSD11B1,MGST1,SULT1A1,SULT1A2,SULT1A3,SULT1A4 |
| 153910 | Phagosome | 3.620143426 | 11 | C1R,HLA-A,ITGAV,ITGB2,ITGB5,RAB7B,SCARB1,THBS1,TUBB2A,TUBB2B,TUBB8 |
| 167324 | Amoebiasis | 3.21097747 | 08 | ACTN1,COL1A2,COL3A1,ITGB2,LAMA5,LAMB2,LAMB3,RAB7B |
| 782000 | Proteoglycans in cancer | 3.14746694 | 12 | CCND1,ERBB2,FLNC,GPC1,ITGAV,ITGB5,LUM,MMP9,MSN,SOS1,STAT3,THBS1 |
| 83115 | Bladder cancer | 2.916353651 | 05 | CCND1,ERBB2,MMP9,THBS1,TYMP |
| 169642 | Toxoplasmosis | 2.748907163 | 08 | CYCS,HSPA8,IRAK1,LAMA5,LAMB2,LAMB3,STAT1,STAT3 |
| **Pathway Interaction Database** | | | | |
| 137932 | IL6-mediated signaling events | 2.821528426 | 05 | LBP,SOS1,STAT1,STAT3,TIMP1 |
| 138010 | Glypican 1 network | 2.801555589 | 04 | FGR,GPC1,SLIT2,TGFBR2 |
| 138009 | Plasma membrane estrogen receptor signaling | 2.801555589 | 04 | GNG2,MMP9,MSN,SOS1 |
| 137945 | amb2 Integrin signaling | 2.6061022 | 05 | FGR,HP,ITGB2,MMP9,THY1 |
| 138064 | Syndecan-4-mediated signaling events | 2.511901482 | 04 | ACTN1,CXCL12,MMP9,THBS1 |
| 137910 | CXCR4-mediated signaling events | 2.41318701 | 06 | CXCL12,FGR,GNG2,MMP9,STAT1,STAT3 |
| 169348 | Signaling events mediated by focal adhesion kinase | 2.381190903 | 05 | ACTN1,CCND1,ITGAV,ITGB5,SOS1 |
| 138011 | CXCR3-mediated signaling events | 2.363501461 | 04 | CXCL10,CXCL9,GNG2,ITGB2 |
| 169349 | Validated transcriptional targets of AP1 family members Fra1 and Fra2 | 2.363501461 | 04 | CCND1,COL1A2,HMOX1,MMP9 |
| 169352 | Regulation of Wnt-mediated beta catenin signaling and target gene transcription | 2.33010021 | 06 | CCND1,CCND2,KLF4,MMP9,TLE2,YWHAH |
| **REACTOME** | | | | |
| 1270245 | Collagen formation | 10 | 14 | COL11A1,COL12A1,COL15A1,COL16A1,COL1A2,COL3A1,COL5A2,COL6A1,COL6A2,CRTAP,LAMB3,LOX,MMP9,SERPINH1 |
| 1269897 | Binding and Uptake of Ligands by Scavenger Receptors | 10 | 09 | COL1A2,COL3A1,FTH1,HBA1,HBB,HP,SAA1,SCARB1,SPARC |
| 1270244 | Extracellular matrix organization | 10 | 34 | ACTN1,ADAMTS5,COL11A1,COL12A1,COL15A1,COL16A1,COL1A2,COL3A1,COL5A2,COL6A1,COL6A2,CRTAP,DAG1,EFEMP1,ELN,FBLN2,FMOD,HTRA1,ITGAV,ITGB2,ITGB5,LAMA5,LAMB2,LAMB3,LOX,LUM,MFAP4,MFAP5,MMP9,SERPINH1,SPARC,SPP1,THBS1,TIMP1 |
| 1270251 | Elastic fibre formation | 5.863438649 | 08 | EFEMP1,ELN,FBLN2,ITGAV,ITGB5,LOX,MFAP4,MFAP5 |
| 1470923 | Interleukin-4 and 13 signaling | 5.599166845 | 12 | CCND1,COL1A2,HMOX1,HSPA8,ITGB2,LAMA5,LBP,MMP9,SAA1,STAT1,STAT3,TIMP1 |
| 1270254 | Non-integrin membrane-ECM interactions | 5.548528425 | 08 | ACTN1,DAG1,ITGAV,ITGB5,LAMA5,LAMB2,LAMB3,THBS1 |
| 1269310 | Cytokine Signaling in Immune system | 5.166203341 | 34 | ANGPT1,APBB1IP,CCL3L3,CCND1,COL1A2,CSF2RA,CXCL10,ERBB2,GRIN2B,HAVCR2,HLA-A,HMOX1,HSPA8,IFIT2,IL1RN,IRAK1,IRF8,ITGB2,LAMA5,LBP,MMP9,MT2A,NUP188,PPP2R1B,SAA1,SOS1,SPTAN1,STAT1,STAT3,TIMP1,TNFRSF11B,TNFRSF12A,TNFRSF25,TRIM6 |
| 1457780 | Neutrophil degranulation | 4.840148593 | 25 | CD68,CST3,FGR,FTH1,GLB1,GNS,HBB,HLA-A,HP,HSPA8,ITGAV,ITGB2,LYZ,MGST1,MMP9,MVP,PLEKHO2,PYGL,RAB31,S100A11,S100A9,SLC2A5,SPTAN1,TNFAIP6,TYROBP |
| 1270256 | ECM proteoglycans | 4.82830206 | 08 | DAG1,FMOD,ITGAV,ITGB5,LAMA5,LAMB2,LUM,SPARC |
| 1269318 | Signaling by Interleukins | 4.289720048 | 25 | ANGPT1,APBB1IP,CCL3L3,CCND1,COL1A2,CSF2RA,CXCL10,ERBB2,GRIN2B,HAVCR2,HMOX1,HSPA8,IL1RN,IRAK1,ITGB2,LAMA5,LBP,MMP9,PPP2R1B,SAA1,SOS1,SPTAN1,STAT1,STAT3,TIMP1 |
| **GenMAPP** | | | | |
| MAP00140 | C21 Steroid hormone metabolism | 1.802432827 | 02 | CYP11A1,HSD11B1 |
| MAP00531 | Glycosaminoglycan degradation | 1.802432827 | 02 | GLB1,GNS |
| MAP00510 | N Glycans biosynthesis | 1.462554363 | 02 | B4GALT5,MGAT3 |
| **MSigDB C2 BIOCARTA** | | | | |
| M3005 | Genes encoding collagen proteins | 10 | 09 | COL11A1,COL12A1,COL15A1,COL16A1,COL1A2,COL3A1,COL5A2,COL6A1,COL6A2 |
| M3008 | Genes encoding structural ECM glycoproteins | 10 | 21 | CTHRC1,EFEMP1,ELN,FBLN2,IGFBP5,IGFBP6,LAMA5,LAMB2,LAMB3,MFAP4,MFAP5,MFGE8,MXRA5,RSPO3,SLIT2,SPARC,SPP1,SRPX2,SVEP1,THBS1,TNFAIP6 |
| M5889 | Ensemble of genes encoding extracellular matrix and extracellular matrix-associated proteins | 10 | 63 | ADAMTS5,ADAMTSL4,ANGPT1,CCL3L3,CLEC3B,COL11A1,COL12A1,COL15A1,COL16A1,COL1A2,COL3A1,COL5A2,COL6A1,COL6A2,CST3,CTHRC1,CXCL10,CXCL12,CXCL9,EFEMP1,EGFL6,ELN,FBLN2,FGF11,FMOD,FRZB,FSTL3,GPC1,HTRA1,IGFBP5,IGFBP6,IL1RN,INHBB,ITIH5,LAMA5,LAMB2,LAMB3,LEP,LOX,LUM,MFAP4,MFAP5,MFGE8,MMP9,MXRA5,PDGFD,PLXNA1,RSPO3,S100A11,S100A3,S100A4,S100A9,SCUBE2,SEMA3G,SERPINH1,SFRP4,SLIT2,SPARC,SPP1,SRPX2,SVEP1,THBS1,TIMP1,TNFAIP6 |
| M5884 | Ensemble of genes encoding core extracellular matrix including ECM glycoproteins, collagens and proteoglycans | 10 | 32 | COL11A1,COL12A1,COL15A1,COL16A1,COL1A2,COL3A1,COL5A2,COL6A1,COL6A2,CTHRC1,EFEMP1,ELN,FBLN2,FMOD,IGFBP5,IGFBP6,LAMA5,LAMB2,LAMB3,LUM,MFAP4,MFAP5,MFGE8,MXRA5,RSPO3,SLIT2,SPARC,SPP1,SRPX2,SVEP1,THBS1,TNFAIP6 |
| M5885 | Ensemble of genes encoding ECM-associated proteins including ECM-affilaited proteins, ECM regulators and secreted factors | 4.474941666 | 32 | ADAMTS5,ADAMTSL4,ANGPT1,CCL3L3,CLEC3B,CST3,CXCL10,CXCL12,CXCL9,EGFL6,FGF11,FRZB,FSTL3,GPC1,HTRA1,IL1RN,INHBB,ITIH5,LEP,LOX,MMP9,PDGFD,PLXNA1,S100A11,S100A3,S100A4,S100A9,SCUBE2,SEMA3G,SERPINH1,SFRP4,TIMP1 |
| M5883 | Genes encoding secreted soluble factors | 4.27355877 | 19 | ANGPT1,CCL3L3,CXCL10,CXCL12,CXCL9,EGFL6,FGF11,FRZB,FSTL3,IL1RN,INHBB,LEP,PDGFD,S100A11,S100A3,S100A4,S100A9,SCUBE2,SFRP4 |
| M5887 | Genes encoding structural components of basement membranes | 3.91235413 | 06 | COL15A1,COL6A1,COL6A2,LAMA5,LAMB2,LAMB3 |
| M6778 | IL-10 Anti-inflammatory Signaling Pathway | 2.37453478 | 03 | HMOX1,STAT1,STAT3 |
| M18342 | G alpha i Pathway | 2.317474526 | 04 | CFB,DAG1,SOS1,STAT3 |
| M917 | Complement Pathway | 2.233353975 | 03 | C1R,C1S,CFB |
| **PantherDB** | | | | |
| P00034 | Integrin signalling pathway | 10 | 19 | ACTN1,COL11A1,COL12A1,COL15A1,COL16A1,COL1A2,COL3A1,COL5A2,COL6A1,COL6A2,ITGAV,ITGB2,ITGB5,LAMA5,LAMB2,LAMB3,RHOC,RND3,SOS1 |
| P00008 | Axon guidance mediated by Slit/Robo | 2.301673714 | 03 | CXCL12,RHOC,SLIT2 |
| P00049 | Parkinson disease | 2.127254382 | 06 | FGR,HSPA5,HSPA8,SNCG,UCHL1,YWHAH |
| P00005 | Angiogenesis | 2.006215782 | 08 | ANGPT1,FRZB,GRB14,PDGFD,RHOC,SOS1,STAT1,STAT3 |
| P00031 | Inflammation mediated by chemokine and cytokine signaling pathway | 1.864244118 | 09 | CCL3L3,COL12A1,COL6A1,COL6A2,CXCL10,GNG2,RHOC,SOS1,STAT3 |
| P04395 | Vasopressin synthesis | 1.720921143 | 02 | CPE,OXT |
| P00038 | JAK/STAT signaling pathway | 1.410153707 | 02 | STAT1,STAT3 |
| P04393 | Ras Pathway | 1.343390185 | 04 | RHOC,SOS1,STAT1,STAT3 |
| P00016 | Cytoskeletal regulation by Rho GTPase | 1.323968148 | 04 | RHOC,TUBB2A,TUBB2B,TUBB8 |
| **Pathway Ontology** | | | | |
| PW:0000021 | hypertension | 4.522214868 | 03 | HMOX1,LOX,SOD2 |
| PW:0000286 | integrin signaling | 2.188340257 | 04 | APBB1IP,ITGAV,ITGB2,ITGB5 |
| PW:0000498 | reverse cholesterol transport | 1.802432827 | 02 | PLTP,SCARB1 |
| PW:0000378 | oxidative stress responses | 1.695428398 | 01 | NQO1 |
| PW:0000138 | Vitamin B6 metabolic | 1.695428398 | 01 | AOX1 |
| PW:0000346 | CADASIL | 1.695428398 | 01 | NOTCH3 |
| PW:0000300 | renal disease, renal disorder | 1.695428398 | 01 | SPP1 |
| PW:0000413 | heme catabolic | 1.695428398 | 01 | HMOX1 |
| PW:0000169 | nerve growth factor signaling | 1.695428398 | 01 | SOS1 |
| PW:0000238 | insulin-like growth factor signaling | 1.647369002 | 02 | IGFBP5,IGFBP6 |
| **SMPDB** | | | | |
| SMP00101 | Suprofen Pathway | 2.116454504 | 02 | AKR1C3,PTGDS |
| SMP00109 | Mefanamic acid Pathway | 2.116454504 | 02 | AKR1C3,PTGDS |
| SMP00104 | Indomethacin Pathway | 2.116454504 | 02 | AKR1C3,PTGDS |
| SMP00077 | Piroxicam Pathway | 2.116454504 | 02 | AKR1C3,PTGDS |
| SMP00093 | Diclofenac Pathway | 2.116454504 | 02 | AKR1C3,PTGDS |
| SMP00120 | Naproxen Pathway | 2.116454504 | 02 | AKR1C3,PTGDS |
| SMP00085 | Ketoprofen Pathway | 2.116454504 | 02 | AKR1C3,PTGDS |
| SMP00130 | Steroidogenesis | 1.997182265 | 02 | CYP11A1,HSD11B1 |
| SMP00371 | Congenital Lipoid Adrenal Hyperplasia (CLAH) or Lipoid CAH | 1.695428398 | 01 | CYP11A1 |
| SMP00202 | MNGIE (Mitochondrial Neurogastrointestinal Encephalopathy) | 1.695428398 | 01 | TYMP |

**Table 4** The enriched pathway terms of the down-regulated differentially expressed genes

| **BIOCYC** | | | | |
| --- | --- | --- | --- | --- |
| **Pathway ID** | **Pathway Name** | **LogP** | **Gene Count** | **Genes** |
| 142373 | superpathway of methionine degradation | 4.000296992 | 04 | BCKDHA,CBS,MAT2B,PCCA |
| 142421 | fatty acid beta-oxidation | 3.86108057 | 04 | ACADL,ECI1,SCP2,SLC27A2 |
| **KEGG** | | | | |
| 83036 | Ribosome | 10 | 18 | RPL14,RPL17,RPL18,RPL18A,RPL21,RPL29,RPL35,RPL9,RPS12,RPS15A,RPS17,RPS18,RPS2,RPS27,RPS29,RPS3,RPS9,RSL24D1 |
| 83004 | Propanoate metabolism | 5.564719729 | 07 | ACACA,ACACB,ACSS2,ALDH6A1,BCKDHA,LDHC,PCCA |
| 989139 | AMPK signaling pathway | 4.488377132 | 11 | ACACA,ACACB,CAB39L,CD36,EEF2K,FBP1,FOXO1,FOXO3,IGF1,PFKFB3,SIRT1 |
| 82949 | Glycine, serine and threonine metabolism | 3.864500032 | 06 | CBS,CTH,GATM,GLYCTK,PHGDH,SHMT1 |
| 83002 | Glyoxylate and dicarboxylate metabolism | 3.667203873 | 05 | GLUL,GLYCTK,HYI,PCCA,SHMT1 |
| 921162 | FoxOsignaling pathway | 3.474041745 | 10 | CCNG2,CDKN1B,FOXO1,FOXO3,GADD45B,IGF1,MAPK10,PTEN,RBL2,SIRT1 |
| 377873 | Herpes simplex infection | 3.430846606 | 12 | C5,CSNK2A2,CSNK2B,EEF1D,HLA-DMA,MAPK10,NOP53,PER2,PER3,SRSF3,SRSF4,SRSF7 |
| 868084 | Fatty acid metabolism | 3.41818559 | 07 | ACACA,ACADL,ELOVL6,FADS1,HACD1,HSD17B12 |
| 132956 | Metabolic pathways | 3.326019848 | 43 | AASS,ACACA,ACACB,ACADL,ACSS2,ADH1B,ADH1C,ALDH6A1,BCAT2,BCKDHA,BDH1,BLVRB,CBS,CDS2,CKB,CTH,FBP1,GATM,GLUL,GLYCTK,GMPS,GPAT3,GPHN,HSD17B12,HYI,LDHC,LSS,MAT2B,MOCS1,NDUFV3,PAPSS1,PCCA,PCYT2,PHGDH,PLD1,PMM1,POLR1D,POLR1E,POLR3C,SC5D,SCP2,SHMT1,ST3GAL6 |
| 790012 | Biosynthesis of amino acids | 3.113855172 | 07 | BCAT2,CBS,CTH,GLUL,MAT2B,PHGDH,SHMT1 |
| **Pathway Interaction Database** | | | | |
| 138036 | FoxO family signaling | 4.344156036 | 07 | CDKN1B,FOXO1,FOXO3,MAPK10,RBL2,SIRT1,ZFAND5 |
| 137956 | HIF-2-alpha transcription factor network | 3.258024576 | 05 | BHLHE40,EFNA1,ELK1,SIRT1,TWIST1 |
| 137963 | Regulation of nuclear SMAD2/3 signaling | 3.045281998 | 07 | ESR1,FOXO1,FOXO3,NR3C1,RBBP4,SIN3A,TGIF1 |
| 137972 | Signaling events mediated by HDAC Class III | 2.977804515 | 05 | ACSS2,FOXO1,FOXO3,HOXA10,SIRT1 |
| 137979 | FOXA1 transcription factor network | 2.782996939 | 05 | CDKN1B,ESR1,NDUFV3,NRIP1,SFTPD |
| 138020 | Class I PI3K signaling events mediated by Akt | 2.332504648 | 04 | CDKN1B,FOXO1,FOXO3,MAP3K5 |
| 138027 | Regulation of Androgen receptor activity | 1.744963151 | 04 | FOXO1,NR3C1,SIRT1,TRIM24 |
| 137916 | Regulation of retinoblastoma protein | 1.582589283 | 04 | CDKN1B,RBBP4,SFTPD,SIRT1 |
| 138062 | Signaling events mediated by HDAC Class II | 1.514289673 | 03 | ESR1,HDAC9,NR3C1 |
| 137997 | Signaling events mediated by HDAC Class I | 1.356649762 | 04 | HDAC9,RBBP4,SIN3A,SIRT1 |
| **REACTOME** | | | | |
| 1268690 | Eukaryotic Translation Elongation | 10 | 19 | EEF1D,EEF1G,RPL14,RPL17,RPL18,RPL18A,RPL21,RPL29,RPL35,RPL9,RPS12,RPS15A,RPS17,RPS18,RPS2,RPS27,RPS29,RPS3,RPS9 |
| 1270158 | Metabolism of amino acids and derivatives | 10 | 34 | AASS,ALDH6A1,AZIN1,BCAT2,BCKDHA,CBS,CKB,CTH,GATM,GLUL,NAALAD2,PAPSS1,PCBD1,PHGDH,RPL14,RPL17,RPL18,RPL18A,RPL21,RPL29,RPL35,RPL9,RPS12,RPS15A,RPS17,RPS18,RPS2,RPS27,RPS29,RPS3,RPS9,SHMT1,SLC3A2,TMLHE |
| 1270010 | Fatty acid, triacylglycerol, and ketone body metabolism | 10 | 19 | AACS,ACACA,ACADL,AGT,BDH1,CD36,ECI1,ELOVL6,FADS1,GPAT3,GPD1L,HACD1,HSD17B12,MED16,MED28,MED30,MED6,PCCA,SIN3A |
| 1270001 | Metabolism of lipids and lipoproteins | 4.373325922 | 34 | AACS,ACACA,ACACB,ACADL,AGT,BDH1,CD36,CDS2,CSNK2A2,CSNK2B,ECI1,ELOVL6,FABP5,FADS1,GPAT3,GPD1L,HACD1,HSD17B11,HSD17B12,LSS,MED16,MED28,MED30,MED6,ORMDL3,OSBPL1A,PCCA,PCYT2,PLD1,PTEN,SC5D,SCP2,SIN3A,SLC27A2 |
| 1270161 | Branched-chain amino acid catabolism | 4.002640203 | 05 | ALDH6A1,BCAT2,BCKDHA,SHMT1,TMLHE |
| 1268854 | Disease | 2.588390587 | 30 | ACACA,ARRB1,CCDC59,CD36,CDKN1B,FOXO1,FOXO3,GPC3,HDAC9,PAPSS1,PCCA,RPL14,RPL17,RPL18,RPL18A,RPL21,RPL29,RPL35,RPL9,RPS12,RPS15A,RPS17,RPS18,RPS2,RPS27,RPS29,RPS3,RPS9,SFTPD,TRIM24 |
| 1269871 | Circadian Clock | 2.518155723 | 06 | BHLHE40,DBP,NR3C1,NRIP1,PER2,SIRT1 |
| 1269652 | Nuclear Receptor transcription pathway | 2.452176105 | 05 | ESR1,NR2F1,NR3C1,NR4A2,RORB |
| 1270168 | Carnitine synthesis | 2.410152815 | 02 | SHMT1,TMLHE |
| 1270029 | Regulation of lipid metabolism by Peroxisome proliferator-activated receptor alpha (PPARalpha) | 2.380171215 | 08 | AGT,CD36,FADS1,MED16,MED28,MED30,MED6,SIN3A |
| **GenMAPP** | | | | |
| MAP00640 | Propanoate metabolism | 4.411975751 | 05 | ACACA,ACACB,ALDH6A1,LDHC,PCCA |
| MAP00450 | Selenoamino acid metabolism | 2.239863267 | 02 | CBS,CTH |
| MAP00910 | Nitrogen metabolism | 2.08446222 | 03 | CA3,CTH,GLUL |
| MAP00271 | Methionine metabolism | 1.980370918 | 02 | CBS,CTH |
| MAP00272 | Cysteine metabolism | 1.87699911 | 02 | CTH,LDHC |
| MAP00280 | Valineleucine and isoleucine degradation | 1.825387093 | 03 | ALDH6A1,BCKDHA,PCCA |
| MAP00100 | Sterol biosynthesis | 1.785852022 | 02 | LSS,SC5D |
| MAP00010 | Glycolysis Gluconeogenesis | 1.688196341 | 04 | ADH1B,ADH1C,FBP1,LDHC |
| MAP00620 | Pyruvate metabolism | 1.619507881 | 03 | ACACA,ACACB,LDHC |
| MAP00310 | Lysine degradation | 1.394254793 | 02 | AASS,SHMT1 |
| **MSigDB C2 BIOCARTA** | | | | |
| M6682 | CDK Regulation of DNA Replication | 2.277315691 | 03 | CDKN1B,MCM6,ORC2 |
| M10145 | PTEN dependent cell cycle arrest and apoptosis | 2.277315691 | 03 | CDKN1B,FOXO3,PTEN |
| M12012 | p38 MAPK Pathway | 2.199389482 | 04 | EEF2K,ELK1,MAP3K5,MKNK2 |
| M4170 | Interferon gamma pathway. | 1.785852022 | 02 | CISH,ELP2 |
| M5374 | The SARS-coronavirus Life Cycle | 1.785852022 | 02 | FBL,LDHC |
| M287 | Erk1/Erk2 MapkSignaling pathway | 1.737787907 | 03 | ELK1,MKNK2,RPS6KA5 |
| M13863 | MAPKinaseSignaling Pathway | 1.507327629 | 05 | ELK1,MAP3K5,MAPK10,MKNK2,RPS6KA5 |
| M2499 | CARM1 and Regulation of the Estrogen Receptor | 1.481705587 | 03 | ESR1,HDAC9,NRIP1 |
| M13158 | The IGF-1 Receptor and Longevity | 1.446542824 | 02 | FOXO3,IGF1 |
| M14532 | PI3K Pathway | 1.419841166 | 03 | CD55,FOXO1,PTEN |
| **PantherDB** | | | | |
| P05918 | p38 MAPK pathway | 3.140376991 | 05 | EEF2K,ELK1,MAP3K5,MKNK2,RPS6KA5 |
| P02776 | Serine glycine biosynthesis | 2.410152815 | 02 | PHGDH,SHMT1 |
| P00015 | Circadian clock system | 1.87699911 | 02 | PER2,PER3 |
| P00035 | Interferon-gamma signaling pathway | 1.737787907 | 03 | CISH,MAPK10,SOCS2 |
| P00032 | Insulin/IGF pathway-mitogen activated protein kinase kinase/MAP kinase cascade | 1.696738708 | 03 | ELK1,IGF1,RPS6KA5 |
| P02737 | Cysteine biosynthesis | 1.68650448 | 01 | CBS |
| P00033 | Insulin/IGF pathway-protein kinase B signaling cascade | 1.514289673 | 03 | FOXO3,IGF1,PTEN |
| P05911 | Angiotensin II-stimulated signaling through G proteins and beta-arrestin | 1.481705587 | 03 | AGT,ARRB1,ELK1 |
| P00036 | Interleukin signaling pathway | 1.417751205 | 05 | CDKN1B,ELK1,FOXO3,IL13RA1,MKNK2 |
| P02724 | Alanine biosynthesis | 1.398756651 | 01 | BCAT2 |
| **Pathway Ontology** | | | | |
| PW:0000047 | glycine, serine and threonine metabolic | 5.257885042 | 05 | CBS,CTH,GATM,PHGDH,SHMT1 |
| PW:0000400 | transulfuration of homocysteine metabolism | 3.383735276 | 02 | CBS,CTH |
| PW:0000198 | p38 MAPK signaling | 3.052433182 | 04 | IGF1,MAP3K5,MKNK2,RPS6KA5 |
| PW:0000427 | molybdenum cofactor biosynthetic | 2.626189633 | 02 | GPHN,MOCS1 |
| PW:0000510 | altered insulin-like growth factor signaling | 1.68650448 | 01 | IGF1 |
| PW:0000371 | calcium homeostasis | 1.68650448 | 01 | S100A1 |
| PW:0000250 | peptidoglycan biosynthetic | 1.68650448 | 01 | GLUL |
| PW:0000076 | pathway of urea cycle and metabolism of amino groups | 1.564200004 | 02 | CKB,GATM |
| PW:0000008 | Wntsignaling | 1.548065942 | 03 | MAPK10,WNT11,WNT3 |
| PW:0000440 | glycine metabolic | 1.398756651 | 01 | SHMT1 |
| **SMPDB** | | | | |
| SMP00004 | Glycine, Serine and Threonine Metabolism | 5.090440722 | 06 | CBS,CTH,GATM,GLYCTK,PHGDH,SHMT1 |
| SMP00029 | Selenoamino Acid Metabolism | 3.206449708 | 02 | CBS,CTH,PAPSS1 |
| SMP00033 | Methionine Metabolism | 2.511627198 | 03 | CBS,CTH,SHMT1 |
| SMP00071 | Ketone Body Metabolism | 2.410152815 | 02 | BDH1,CTH |
| SMP00384 | MethylmalonateSemialdehyde Dehydrogenase Deficiency | 1.68650448 | 01 | ALDH6A1 |
| SMP00239 | Saccharopinuria/Hyperlysinemia II | 1.68650448 | 01 | AASS |
| SMP00362 | Arginine: Glycine Amidinotransferase Deficiency (AGAT Deficiency) | 1.68650448 | 01 | GATM |
| SMP00177 | Cystathionine Beta-Synthase Deficiency | 1.68650448 | 01 | CBS |
| SMP00032 | Valine, Leucine and Isoleucine Degradation | 1.657349818 | 03 | ALDH6A1,BCKDHA,PCCA |
| SMP00016 | Propanoate Metabolism | 1.631017126 | 02 | ALDH6A1,PCCA |

**Table 5** The enriched GO terms of the up-regulated differentially expressed genes

| **GO ID** | **CATEGORY** | **GO Name** | **logP** | **Gene Count** | **Genes** |
| --- | --- | --- | --- | --- | --- |
| GO:0048514 | BP | blood vessel morphogenesis | 10 | 32 | AMOT,ANGPT1,CCDC80,COL15A1,COL3A1,CXCL10,CXCL12,ERBB2,HMOX1,ITGAV,ITGB2,KLF4,LAMA5,LEP,MFGE8,MMP9,NOTCH3,NRCAM,NRP2,PTGER4,RSPO3,SLIT2,SOS1,SPARC,SRPX2,STAT1,TGFBR2,THBS1,THY1,TNFRSF12A,TNMD,TYMP |
| GO:0072358 | BP | cardiovascular system development | 10 | 45 | AMOT,ANGPT1,APLNR,CCDC80,COL11A1,COL15A1,COL1A2,COL3A1,CPE,CRIP1,CXCL10,CXCL12,ERBB2,HMOX1,ITGAV,ITGB2,KLF4,LAMA5,LEP,LOX,MARCKS,MFGE8,MMP9,NOTCH3,NPY5R,NRCAM,NRP2,OXT,PDGFD,PTGER4,RSPO3,SLIT2,SMYD2,SOD2,SOS1,SPARC,SRPX2,STAT1,TGFBR2,THBS1,THY1,TNFRSF12A,TNMD,TYMP,VLDLR |
| GO:0051241 | BP | negative regulation of multicellular organismal process | 10 | 47 | AMOT,ANGPT1,COL3A1,COL5A2,CORO1C,CPE,CST3,CXCL10,EFEMP1,ERBB2,FRZB,FSTL3,GPNMB,HAVCR2,HLA-A,HMOX1,HOXB8,IGFBP5,INHBB,KLF4,LBP,LEP,LMO2,LRRC32,MBP,NOTCH3,OXT,PTGDS,PTGER4,SEMA3G,SLIT2,SPARC,SPP1,STAT1,STAT3,STC2,STMN2,TGFBR2,THBS1,THY1,TIMP1,TMEM178A,TNFRSF11B,TNMD,VASN,WWC1,YWHAH |
| GO:0072359 | BP | circulatory system development | 10 | 45 | AMOT,ANGPT1,APLNR,CCDC80,COL11A1,COL15A1,COL1A2,COL3A1,CPE,CRIP1,CXCL10,CXCL12,ERBB2,HMOX1,ITGAV,ITGB2,KLF4,LAMA5,LEP,LOX,MARCKS,MFGE8,MMP9,NOTCH3,NPY5R,NRCAM,NRP2,OXT,PDGFD,PTGER4,RSPO3,SLIT2,SMYD2,SOD2,SOS1,SPARC,SRPX2,STAT1,TGFBR2,THBS1,THY1,TNFRSF12A,TNMD,TYMP,VLDLR |
| GO:0001944 | BP | vasculature development | 10 | 35 | AMOT,ANGPT1,CCDC80,COL15A1,COL1A2,COL3A1,CXCL10,CXCL12,ERBB2,HMOX1,ITGAV,ITGB2,KLF4,LAMA5,LEP,LOX,MFGE8,MMP9,NOTCH3,NRCAM,NRP2,PDGFD,PTGER4,RSPO3,SLIT2,SOS1,SPARC,SRPX2,STAT1,TGFBR2,THBS1,THY1,TNFRSF12A,TNMD,TYMP |
| GO:0040012 | BP | regulation of locomotion | 10 | 42 | AMOT,ANGPT1,CARMIL1,COL3A1,CORO1C,CXCL10,CXCL12,CXCL9,DAG1,EFEMP1,ERBB2,FGR,GLIPR2,HMOX1,HSPA5,IGFBP5,IL1RN,ITGAV,KLF4,LAMA5,LBP,MMP9,MSN,NRP2,PDGFD,PLA2G7,PLXNA1,PTGER4,SCARB1,SEMA3G,SGK1,SH3BGRL3,SHTN1,SLIT2,SPARC,SRPX2,STAT3,TGFBR2,THBS1,THY1,TIMP1,TNFAIP6 |
| GO:0043062 | BP | extracellular structure organization | 10 | 36 | ADAMTS5,ADAMTSL4,CCDC80,COL11A1,COL12A1,COL16A1,COL1A2,COL3A1,COL5A2,COL6A1,COL6A2,CST3,DAG1,EGFL6,ELN,FMOD,HSPA8,HTRA1,ITGAV,ITGB2,ITGB5,LAMA5,LAMB2,LAMB3,LCP1,LOX,LUM,MFAP4,MFAP5,MMP9,SERPINH1,SPARC,SPP1,THBS1,TIMP1,TNFRSF11B |
| GO:0016477 | BP | cell migration | 10 | 69 | AMOT,ANGPT1,BAMBI,CARMIL1,CCL3L3,CD248,COL1A2,COL3A1,CORO1C,CTHRC1,CXCL10,CXCL12,CXCL9,DAG1,EFEMP1,EMX2,FAT1,FGR,GLIPR2,GRB14,HMOX1,HSPA5,IGFBP5,IL1RN,ITGAV,ITGB2,KLF4,LAMA5,LBP,LCP1,LEP,LHX6,MMP9,MSN,NAV1,NRCAM,NRP2,PALLD,PDGFD,PLA2G7,PLPP1,PLXNA1,PTGER4,RHOC,S100A9,SAA1,SAA2,SAA4,SCARB1,SEMA3G,SGK1,SH3BGRL3,SHTN1,SLIT2,SOS1,SPARC,SPP1,SRPX2,STAT1,STAT3,TGFBR2,THBS1,THY1,TIMP1,TNFAIP6,TNFRSF12A,TNS3,TUBB2B,WWC1 |
| GO:0033993 | BP | response to lipid | 10 | 42 | AKR1C3,ALPL,CCND1,CXCL10,CXCL9,GNG2,HAVCR2,HMOX1,HNMT,IL1RN,INHBB,IRAK1,IRF8,KLF4,LBP,LEP,LOX,MBP,MFGE8,MGST1,MSN,NQO1,OXT,PLPP1,PMEPA1,PTGDS,PTGER4,SCARB1,SGK1,SLIT2,SPARC,SPP1,STAT1,STAT3,STC2,TGFBR2,THBS1,TNFRSF11B,TNFRSF25,TRIM6,VLDLR,YWHAH |
| GO:0031589 | BP | cell-substrate adhesion | 10 | 21 | ACTN1,ANGPT1,CARMIL1,CCDC80,COL16A1,COL3A1,CORO1C,DAG1,EGFL6,EPDR1,FBLN2,ITGAV,ITGB2,ITGB5,LAMA5,MARCKS,SGCE,SPP1,THBS1,THY1,TNFRSF12A |
| GO:0031012 | CC | extracellular matrix | 10 | 41 | ADAMTS5,ADAMTSL4,ALPL,CCDC80,CD248,CLEC3B,COL11A1,COL12A1,COL15A1,COL16A1,COL1A2,COL3A1,COL5A2,COL6A1,COL6A2,CRTAP,CST3,CTHRC1,DAG1,EFEMP1,EGFL6,ELN,FBLN2,FCGBP,FMOD,GPC1,HTRA1,LAMA5,LAMB2,LAMB3,LOX,LUM,MFAP4,MFAP5,MFGE8,MMP9,SLIT2,SPARC,THBS1,TIMP1,TNFRSF11B |
| GO:0044420 | CC | extracellular matrix component | 10 | 21 | CCDC80,COL11A1,COL12A1,COL15A1,COL1A2,COL3A1,COL5A2,COL6A1,CST3,DAG1,EFEMP1,EGFL6,ELN,LAMA5,LAMB2,LAMB3,LUM,MFAP4,MFAP5,SPARC,TIMP1 |
| GO:0005615 | CC | extracellular space | 10 | 87 | ACTN1,ADAMTS5,AIFM2,ALPL,ANGPT1,C1R,C1RL,C1S,CCL3L3,CD9,CES1,CFB,CFH,CHI3L2,CLEC3B,CMTM3,COL12A1,COL15A1,COL1A2,COL3A1,COL6A2,CPE,CRTAP,CSN1S1,CST3,CTHRC1,CXCL10,CXCL12,CXCL9,DAG1,EFEMP1,EGFL6,FMOD,FRZB,FSTL3,GLB1,GPC1,HBA1,HBB,HLA-A,HMOX1,HP,HSPA8,HTRA1,IGFBP5,IGFBP6,IL1RN,INHBB,LAMA5,LBP,LCP1,LEP,LHCGR,LOX,LUM,LYZ,MFGE8,MMP9,MSN,OXT,PDGFD,PLA2G7,PLTP,PPFIBP2,PTGDS,S100A11,S100A4,S100A9,SAA1,SAA2,SAA4,SCUBE2,SEMA3G,SERPINH1,SFRP4,SLIT2,SPARC,SPP1,SRPX2,STC2,THBS1,TIMP1,TLE2,TNFAIP6,TNFRSF11B,VASN,VLDLR |
| GO:0005788 | CC | endoplasmic reticulum lumen | 10 | 19 | ADAMTS5,ADAMTSL4,CALU,CES1,COL11A1,COL12A1,COL15A1,COL16A1,COL1A2,COL3A1,COL5A2,COL6A1,COL6A2,CRTAP,HSPA5,LYZ,PDGFD,SERPINH1,THBS1 |
| GO:0005581 | CC | collagen trimer | 10 | 12 | COL11A1,COL12A1,COL15A1,COL16A1,COL1A2,COL3A1,COL5A2,COL6A1,COL6A2,CTHRC1,LOX,LUM |
| GO:0005912 | CC | adherens junction | 5.456429066 | 25 | ACTN1,ALCAM,APBB1IP,CADM3,CD9,CORO1C,DAG1,FAM129B,FAT1,FLNC,HSPA5,HSPA8,ITGAV,ITGB5,LCP1,MARCKS,MSN,PALLD,PDLIM1,RND3,SPTAN1,SYNPO2,THY1,TLE2,TNS3 |
| GO:0005925 | CC | focal adhesion | 5.418442901 | 22 | ACTN1,ALCAM,APBB1IP,CD9,CORO1C,DAG1,FAT1,FLNC,HSPA5,HSPA8,ITGAV,ITGB5,LCP1,MARCKS,MSN,PALLD,PDLIM1,RND3,SYNPO2,THY1,TLE2,TNS3 |
| GO:0005604 | CC | basement membrane | 5.289701566 | 11 | CCDC80,COL15A1,CST3,DAG1,EFEMP1,EGFL6,LAMA5,LAMB2,LAMB3,SPARC,TIMP1 |
| GO:0071682 | CC | endocytic vesicle lumen | 4.986142167 | 05 | HBA1,HBB,HP,SAA1,SPARC |
| GO:0048471 | CC | perinuclear region of cytoplasm | 3.401186527 | 27 | CST3,DOCK6,ERBB2,FAT1,GLB1,HMOX1,HSPA8,INF2,INHBB,MAP1B,MSN,MT2A,MVP,NANOS3,PTGDS,S100A4,SGK1,SHTN1,SLC2A10,SNCG,SPP1,STAT1,STC2,STMN2,SYNC,VLDLR,WWC1 |
| GO:0019838 | MF | growth factor binding | 10 | 15 | COL1A2,COL3A1,COL6A1,ERBB2,GPC1,HTRA1,IGFBP5,IGFBP6,IL1RN,ITGAV,NRP2,SRPX2,TGFBR2,THBS1,VASN |
| GO:0005178 | MF | integrin binding | 10 | 13 | ACTN1,ADAMTS5,CD9,COL16A1,COL3A1,EGFL6,GPNMB,ITGB5,LAMA5,LAMB2,MFGE8,THBS1,THY1 |
| GO:0050839 | MF | cell adhesion molecule binding | 10 | 19 | ACTN1,ADAMTS5,CADM3,CD9,COL16A1,COL3A1,CPE,EGFL6,GPNMB,GRIN2B,ITGB2,ITGB5,LAMA5,LAMB2,MFGE8,MSN,SHTN1,THBS1,THY1 |
| GO:0050840 | MF | extracellular matrix binding | 10 | 11 | ADAMTS5,CD248,COL11A1,DAG1,FBLN2,GPC1,ITGAV,SLIT2,SPARC,SPP1,THBS1 |
| GO:0005201 | MF | extracellular matrix structural constituent | 5.80617847 | 10 | COL11A1,COL12A1,COL15A1,COL1A2,COL3A1,COL5A2,ELN,FBLN2,LUM,MFAP5 |
| GO:0005509 | MF | calcium ion binding | 5.576968244 | 32 | ACTN1,C1R,C1S,CALB2,CALU,CD248,CLEC3B,DAG1,EFEMP1,EGFL6,EPDR1,FAT1,FBLN2,HSPA5,LCP1,MYL9,NOTCH3,PCDH9,S100A11,S100A3,S100A4,S100A9,SCIN,SCUBE2,SLC24A3,SLIT2,SPARC,SPTAN1,SUSD1,SVEP1,THBS1,VLDLR |
| GO:0005539 | MF | glycosaminoglycan binding | 4.874249306 | 15 | ADAMTS5,CCDC80,CFH,CLEC3B,CXCL10,FMOD,GNS,GPNMB,NRP2,RSPO3,SAA1,SLIT2,TGFBR2,THBS1,TNFAIP6 |
| GO:0003779 | MF | actin binding | 4.582186708 | 21 | ACTN1,CORO1C,DAG1,ENC1,FLNC,INF2,LCP1,LMOD1,MAP1B,MARCKS,MSN,PALLD,S100A4,SCIN,SHTN1,SPTAN1,STK38L,SYNPO2,TAGLN,TPM2,YWHAH |
| GO:0001968 | MF | fibronectin binding | 4.577634254 | 06 | CCDC80,FSTL3,IGFBP5,ITGAV,MMP9,THBS1 |
| GO:0001786 | MF | phosphatidylserine binding | 4.577634254 | 06 | HSPA8,MARCKS,MFGE8,SCARB1,SCIN,THBS1 |

BP – Biological processes; CC – Cellular Component; MF –Molecular Function

**Table 6** The enriched GO terms of the down-regulated differentially expressed genes

| **GO ID** | **CATEGORY** | **GO Name** | **logP** | **Gene Count** | **Genes** |
| --- | --- | --- | --- | --- | --- |
| GO:0016053 | BP | organic acid biosynthetic process | 10 | 25 | ACACA,ACACB,ACADL,ACSS2,AGT,BCAT2,CBS,CTH,ELOVL6,FADS1,GATM,GLUL,HACD1,HSD17B12,LDHC,NAALAD2,PDK4,PER2,PHGDH,SC5D,SCP2,SHMT1,SIRT1,SLC27A2,TMLHE |
| GO:0043603 | BP | cellular amide metabolic process | 10 | 45 | ABTB1,ACACA,ACACB,AGT,ANG,BTG2,CTH,EEF1D,EEF1G,EEF2K,EIF2A,EIF3H,EIF4B,EIF4EBP2,ESR1,FOXO3,IGF1,KHDRBS1,LARP6,MKNK2,MRPL55,ORMDL3,PCCA,PER2,RPL14,RPL17,RPL18,RPL18A,RPL21,RPL29,RPL35,RPL9,RPS12,RPS15A,RPS17,RPS18,RPS2,RPS27,RPS29,RPS3,RPS9,RSL24D1,SHMT1,SLC25A23,ST3GAL6 |
| GO:0072599 | BP | establishment of protein localization to endoplasmic reticulum | 10 | 17 | RPL14,RPL17,RPL18,RPL18A,RPL21,RPL29,RPL35,RPL9,RPS12,RPS15A,RPS17,RPS18,RPS2,RPS27,RPS29,RPS3,RPS9 |
| GO:0072330 | BP | monocarboxylic acid biosynthetic process | 10 | 18 | ACACA,ACACB,ACADL,ACSS2,AGT,ELOVL6,FADS1,HACD1,HSD17B12,LDHC,PDK4,PER2,SC5D,SCP2,SHMT1,SIRT1,SLC27A2,TMLHE |
| GO:0006412 | BP | translation | 10 | 37 | ABTB1,ANG,BTG2,EEF1D,EEF1G,EEF2K,EIF2A,EIF3H,EIF4B,EIF4EBP2,ESR1,FOXO3,IGF1,KHDRBS1,LARP6,MKNK2,MRPL55,PER2,RPL14,RPL17,RPL18,RPL18A,RPL21,RPL29,RPL35,RPL9,RPS12,RPS15A,RPS17,RPS18,RPS2,RPS27,RPS29,RPS3,RPS9,RSL24D1,SLC25A23 |
| GO:0006413 | BP | translational initiation | 10 | 22 | EIF2A,EIF3H,EIF4B,EIF4EBP2,KHDRBS1,RPL14,RPL17,RPL18,RPL18A,RPL21,RPL29,RPL35,RPL9,RPS12,RPS15A,RPS17,RPS18,RPS2,RPS27,RPS29,RPS3,RPS9 |
| GO:0019752 | BP | carboxylic acid metabolic process | 10 | 43 | AACS,AASS,ACACA,ACACB,ACADL,ACSS2,ADH1C,AGT,ALDH6A1,AZIN1,BCAT2,BCKDHA,CBS,CD36,CKB,CTH,CYP4V2,ECI1,ELOVL6,FADS1,FBP1,GATM,GLUL,GMPS,HACD1,HSD17B12,IGF1,LDHC,NAALAD2,PCBD1,PCCA,PDK4,PER2,PFKFB3,PHGDH,PLIN5,SC5D,SCP2,SHMT1,SIRT1,SLC27A2,TMLHE,TWIST1 |
| GO:0009057 | BP | macromolecule catabolic process | 4.553158507 | 45 | ARRB1,AZIN1,BTG2,C2orf40,CCNF,CDKN1B,CSNK2A2,CTSC,EIF4B,FBXO11,FOXO1,GJA1,GPC3,H1F0,HERC5,HMGB2,PTEN,RNF125,RNF144A,RNF217,RNF38,RPL14,RPL17,RPL18,RPL18A,RPL21,RPL29,RPL35,RPL9,RPS12,RPS15A,RPS17,RPS18,RPS2,RPS27,RPS29,RPS3,RPS9,SIRT1,SOX17,TRIM24,UBE2W,UBR2,USP13,ZHX2 |
| GO:0006633 | BP | fatty acid biosynthetic process | 4.538876498 | 12 | ACACA,ACACB,ACADL,ACSS2,AGT,ELOVL6,FADS1,HACD1,HSD17B12,PDK4,SC5D,SIRT1 |
| GO:1903507 | BP | negative regulation of nucleic acid-templated transcription | 4.521620083 | 47 | ARRB1,BHLHE40,BTG2,CD36,CDKN1B,CIART,EFNA1,ELF2,ELP2,ESR1,FOXO1,FOXO3,HDAC9,HMGB2,KAT8,KHDRBS1,MAPK10,MKX,MLX,N4BP2L2,NR2F1,NR4A2,NRIP1,ORC2,PER2,PER3,RBM15,RERE,RORB,RPS6KA5,S100A1,SIN3A,SIRT1,SIVA1,SOX17,TBX15,TGIF1,TRIM24,TRIM33,TSC22D3,TWIST1,TXNIP,UBR2,WNT11,ZBTB16,ZHX2,ZNF280D |
| GO:0022627 | CC | cytosolic small ribosomal subunit | 10 | 09 | RPS12,RPS15A,RPS17,RPS18,RPS2,RPS27,RPS29,RPS3,RPS9 |
| GO:0005840 | CC | ribosome | 10 | 19 | MRPL55,RPL14,RPL17,RPL18,RPL18A,RPL21,RPL29,RPL35,RPL9,RPS12,RPS15A,RPS17,RPS18,RPS2,RPS27,RPS29,RPS3,RPS9,RSL24D1 |
| GO:0005730 | CC | nucleolus | 4.057557776 | 34 | ABTB1,ACACA,ANG,BHLHE40,EEF1D,FBL,FBXO11,GTF3C3,H1F0,H1FX,HMGB2,MKNK2,NOP53,NRIP1,PER2,POLR1D,POLR1E,RBL2,RPL18,RPL21,RPL35,RPL9,RPS2,RPS3,RPS9,RSL24D1,SIN3A,SIRT1,STAG2,STK24,STOX1,THYN1,TRIM24,ZBTB16 |
| GO:0000785 | CC | chromatin | 2.602659466 | 19 | AHCTF1,ARRB1,DPF2,ESR1,H1F0,H1FX,HMGB2,NASP,NRIP1,ORC2,RAD21,RBBP4,RBL2,RBMX,SIN3A,SIRT1,STAG2,TRIM24,UBR2 |
| GO:0044451 | CC | nucleoplasm part | 2.467015328 | 25 | CIART,ELP2,FBL,HDAC9,INTS6,KAT14,KAT8,MED16,MED28,MED30,MED6,MKNK2,NRIP1,POLR1D,POLR3C,PTEN,RBBP4,RBM15,RERE,SIN3A,SIRT1,SRSF3,SRSF4,TBX15,ZBTB16 |
| GO:0017053 | CC | transcriptional repressor complex | 2.414075613 | 06 | ELP2,N4BP2L2,RBBP4,SIN3A,TBX15,ZBTB16 |
| GO:1902494 | CC | catalytic complex | 2.343912583 | 33 | ACVR1,ARRB1,BCKDHA,CCNF,CDKN1B,DCAF7,DNAH17,ELP2,FBXO11,GPD1L,HDAC9,INTS6,KAT14,KAT8,MAP3K5,MAT2B,MED30,MED6,NDUFV3,NRIP1,ORMDL3,PAPSS1,POLR1D,POLR1E,POLR3C,PPP3R1,RBBP4,RERE,RNF144A,RNF217,SIN3A,SIRT1,UBR2 |
| GO:1990234 | CC | transferasecomplex | 2.245632262 | 24 | ACVR1,CCNF,CDKN1B,DCAF7,ELP2,FBXO11,HDAC9,INTS6,KAT14,KAT8,MAP3K5,MAT2B,MED30,MED6,ORMDL3,PAPSS1,POLR1D,POLR1E,POLR3C,RBBP4,RNF144A,RNF217,SIRT1,UBR2 |
| GO:0042383 | CC | sarcolemma | 2.070467563 | 08 | ADRB2,ANK2,CD36,DTNA,ESR1,PPP3R1,RTN2,SNTB1 |
| GO:0005694 | CC | chromosome | 1.671134405 | 27 | ADD3,AHCTF1,ARRB1,CCNB1IP1,CENPV,DPF2,ESR1,FBL,FBXO11,H1F0,H1FX,HMGB2,KAT8,MCM6,MIS18BP1,NASP,NRIP1,ORC2,RAD21,RBBP4,RBL2,RBMX,SIN3A,SIRT1,STAG2,TRIM24,UBR2 |
| GO:0003735 | MF | structural constituent of ribosome | 10 | 20 | MRPL55,RPL14,RPL17,RPL18,RPL18A,RPL21,RPL29,RPL35,RPL9,RPS12,RPS15A,RPS17,RPS18,RPS2,RPS27,RPS29,RPS3,RPS9,RSL24D1,SLC25A23 |
| GO:0008134 | MF | transcription factor binding | 5.500226582 | 29 | AIP,ARRB1,BHLHE40,CSNK2B,EEF1D,ELK1,ESR1,FBL,FOXO1,FOXO3,HDAC9,HMGB2,KAT8,MED16,MED30,MED6,MKX,MLX,NR3C1,NR4A2,NRIP1,POLR1E,RORB,RPS3,SIN3A,SIRT1,SOX17,TSC22D3,TWIST1 |
| GO:0044212 | MF | transcription regulatory region DNA binding | 4.619830311 | 35 | ARRB1,BHLHE40,CIART,DBP,DMRT2,DPF2,ELK1,ESR1,FOXO3,HMGB2,HOXA10,KLF9,MKX,MLX,NR2F1,NR3C1,NR4A2,NRIP1,PER2,RBBP4,RBMX,RFX1,RFX2,SAFB,SIN3A,SIRT1,SOX17,STOX1,TBX15,TGIF1,TRIM24,TWIST1,WNT11,ZBTB16,ZNF395 |
| GO:0016829 | MF | lyase activity | 3.979976654 | 13 | BCKDHA,CA3,CBS,CENPV,CTH,GLUL,HACD1,MACROD1,MOCS1,PCBD1,RPS3,SHMT1,SIRT1 |
| GO:0043565 | MF | sequence-specific DNA binding | 3.820984116 | 39 | CIART,CSRNP1,DBP,DMRT2,DPF2,ELF2,ELK1,ESR1,FOXN2,FOXO1,FOXO3,HLF,HMGB2,HOXA10,KLF9,MKX,MLX,NR2F1,NR3C1,NR4A2,NRIP1,ORC2,PER2,RBBP4,RERE,RFX1,RFX2,RORB,SAFB,SIN3A,SIRT1,SOX17,STOX1,TBX15,TGIF1,TRIM24,ZBTB16,ZNF394,ZNF395 |
| GO:0016874 | MF | ligase activity | 3.787075306 | 20 | AACS,ACACA,ACACB,ACSS2,CCNB1IP1,GLUL,GMPS,HERC5,MARCH3,PCCA,PELI2,RNF125,RNF144A,RNF217,RNF38,SLC27A2,TRIM24,TRIM33,TTLL7,UBR2 |
| GO:0000981 | MF | DNA-binding transcription factor activity, RNA polymerase II-specific | 3.202034757 | 26 | BHLHE40,BTG2,CSRNP1,DBP,ELF2,ELK1,ESR1,FOXO1,FOXO3,HOXA10,KLF9,MKX,MLX,NR2F1,NR3C1,NR4A2,RAD21,RFX1,RORB,SOX17,STAG2,TBX15,TGIF1,TWIST1,ZBTB16,ZNF280D |
| GO:0003700 | MF | DNA-binding transcription factor activity | 2.896608329 | 40 | AHCTF1,BHLHE40,BTG2,CNBP,CSRNP1,DBP,DMRT2,ELF2,ELK1,ESR1,FOXN2,FOXO1,FOXO3,HLF,HMGB2,HOXA10,KLF9,MKX,MLX,NR2F1,NR3C1,NR4A2,RAD21,RERE,RFX1,RFX2,RORB,SIN3A,SOX17,STAG2,TBX15,TGIF1,TSC22D1,TSC22D3,TWIST1,ZBTB16,ZHX2,ZNF280D,ZNF334,ZNF394 |
| GO:1990837 | MF | sequence-specific double-stranded DNA binding | 2.677080807 | 26 | CIART,DBP,DMRT2,DPF2,ELK1,ESR1,HMGB2,HOXA10,KLF9,MKX,MLX,NR3C1,NR4A2,NRIP1,ORC2,PER2,RBBP4,RFX1,RFX2,SIN3A,SIRT1,STOX1,TBX15,TGIF1,ZBTB16,ZNF395 |
| GO:0046914 | MF | transition metal ion binding | 2.551740448 | 43 | ADH1B,ADH1C,ANG,CA3,CCNB1IP1,CNBP,CYP4V2,DPF2,DTNA,ESR1,FBXO11,GLUL,MARCH3,MSRB1,MT1G,NR2F1,NR3C1,NR4A2,P3H2,PDLIM3,PHF13,PHF3,PPM1M,RERE,RNF125,RNF144A,RNF217,RNF38,RORB,RPS27,RPS29,SC5D,SIVA1,SRSF7,TMLHE,TRIM24,TRIM33,TRIP4,UBR2,USP13,ZDHHC11,ZFAND1,ZFAND5 |

BP – Biological processes; CC – Cellular Component; MF –Molecular Function

**Table 7** Topology table for up and down regulated genes

| **Regulation** | **Node** | **Degree** | **Betweenness** | **Stress** | **Closeness** |
| --- | --- | --- | --- | --- | --- |
| Up | HSPA8 | 646 | 0.131757 | 1.63E+08 | 0.3772 |
| Up | HSPA5 | 516 | 0.099887 | 1.14E+08 | 0.376106 |
| Up | ERBB2 | 292 | 0.063535 | 48663168 | 0.356491 |
| Up | STAT3 | 264 | 0.05522 | 47126446 | 0.354335 |
| Up | YWHAH | 264 | 0.045286 | 37963446 | 0.341753 |
| Up | LMO2 | 233 | 0.044111 | 33426870 | 0.33642 |
| Up | HLA-A | 220 | 0.014824 | 11862288 | 0.326107 |
| Up | ADAMTSL4 | 209 | 0.038316 | 19994762 | 0.327888 |
| Up | STAT1 | 180 | 0.028372 | 23747148 | 0.343822 |
| Up | SPTAN1 | 177 | 0.024448 | 27747372 | 0.350321 |
| Up | RAB9A | 165 | 0.028366 | 16595428 | 0.322962 |
| Up | NEK6 | 156 | 0.020457 | 28448632 | 0.313392 |
| Up | ACTN1 | 148 | 0.020209 | 23472660 | 0.328689 |
| Up | CEP19 | 141 | 0.023639 | 25737074 | 0.304756 |
| Up | TUBB2A | 137 | 0.015254 | 18211616 | 0.33286 |
| Up | IRAK1 | 124 | 0.015303 | 12259430 | 0.341367 |
| Up | CALU | 122 | 0.018218 | 12821024 | 0.339628 |
| Up | TAGLN2 | 122 | 0.015529 | 11548640 | 0.335163 |
| Up | AMOT | 117 | 0.014715 | 21956260 | 0.319553 |
| Up | MSN | 117 | 0.014992 | 11814740 | 0.333839 |
| Up | FLNC | 116 | 0.016791 | 14264076 | 0.327062 |
| Up | CCND1 | 115 | 0.017274 | 10796534 | 0.331599 |
| Up | VASN | 112 | 0.016584 | 11691916 | 0.320385 |
| Up | S100A9 | 110 | 0.013508 | 13323590 | 0.323624 |
| Up | TPM2 | 107 | 0.013762 | 11390822 | 0.313825 |
| Up | PPP2R1B | 107 | 0.015219 | 10094554 | 0.313122 |
| Up | HOXC8 | 107 | 0.01648 | 15471342 | 0.31475 |
| Up | TUBB8 | 104 | 0.012767 | 8605522 | 0.307178 |
| Up | FTH1 | 103 | 0.01708 | 14706820 | 0.312194 |
| Up | SGK1 | 99 | 0.011615 | 13595420 | 0.313136 |
| Up | CORO1C | 96 | 0.012278 | 9996916 | 0.3286 |
| Up | APLNR | 95 | 0.017519 | 18213738 | 0.28924 |
| Up | TGFBR2 | 94 | 0.014661 | 11193844 | 0.314627 |
| Up | PLIN3 | 93 | 0.014305 | 8824500 | 0.31656 |
| Up | SERPINH1 | 93 | 0.010233 | 10810052 | 0.333793 |
| Up | SPP1 | 92 | 0.015326 | 7687964 | 0.318447 |
| Up | SAR1A | 90 | 0.016122 | 14074460 | 0.317113 |
| Up | MVP | 89 | 0.013541 | 10850118 | 0.333486 |
| Up | TUBB2B | 83 | 0.006011 | 10710364 | 0.306893 |
| Up | EFEMP1 | 83 | 0.013576 | 7822462 | 0.317002 |
| Up | FAM189A2 | 78 | 0.012518 | 23336552 | 0.286627 |
| Up | IGFBP5 | 76 | 0.01285 | 6614978 | 0.288941 |
| Up | UCHL1 | 72 | 0.009707 | 7499836 | 0.333196 |
| Up | MAP1B | 69 | 0.004895 | 4706194 | 0.319961 |
| Up | VGLL3 | 69 | 0.007203 | 18517996 | 0.262443 |
| Up | NOTCH3 | 68 | 0.009978 | 10388152 | 0.307543 |
| Up | SOD2 | 67 | 0.007596 | 13310940 | 0.290512 |
| Up | ITGAV | 66 | 0.008412 | 5317186 | 0.319863 |
| Up | CYCS | 66 | 0.009031 | 4242804 | 0.312087 |
| Up | HAVCR2 | 64 | 0.009241 | 5323290 | 0.302763 |
| Up | ITGB5 | 63 | 0.006615 | 5660860 | 0.311658 |
| Up | CDR2L | 62 | 0.008588 | 10276842 | 0.286469 |
| Up | NAV1 | 61 | 0.007818 | 7186322 | 0.305705 |
| Up | HBB | 60 | 0.009359 | 4158840 | 0.308837 |
| Up | NCKAP5L | 60 | 0.005732 | 7955376 | 0.288953 |
| Up | GLS | 58 | 0.007723 | 6913514 | 0.293433 |
| Up | MBP | 58 | 0.005048 | 8226944 | 0.292618 |
| Up | SOS1 | 57 | 0.004934 | 5230050 | 0.304182 |
| Up | DAG1 | 56 | 0.007357 | 5343410 | 0.309113 |
| Up | S100A4 | 56 | 0.006973 | 9715070 | 0.299345 |
| Up | HMOX1 | 54 | 0.008308 | 5623900 | 0.291807 |
| Up | KIAA1671 | 54 | 0.0045 | 5540890 | 0.289033 |
| Up | QPRT | 53 | 0.006138 | 4832362 | 0.293599 |
| Up | CXCL9 | 52 | 0.006983 | 6107104 | 0.260505 |
| Up | TAGLN | 51 | 0.003826 | 2401428 | 0.299099 |
| Up | OSGIN1 | 50 | 0.004118 | 5186448 | 0.291351 |
| Up | IFIT2 | 50 | 0.003562 | 6888492 | 0.28333 |
| Up | SMYD2 | 49 | 0.005556 | 7843036 | 0.28708 |
| Up | GLB1 | 47 | 0.005936 | 5981072 | 0.297555 |
| Up | RAB6B | 46 | 0.00547 | 4701204 | 0.301094 |
| Up | ZNF219 | 46 | 0.005238 | 3326716 | 0.286277 |
| Up | PCBP3 | 45 | 0.004574 | 3845082 | 0.288815 |
| Up | SULT1A1 | 45 | 0.005119 | 2944316 | 0.296571 |
| Up | LYZ | 44 | 0.003086 | 5138132 | 0.302058 |
| Up | PALLD | 44 | 0.003814 | 2660068 | 0.311993 |
| Up | THBS1 | 44 | 0.00823 | 4538560 | 0.305757 |
| Up | CD9 | 43 | 0.005751 | 2964190 | 0.298239 |
| Up | AEN | 43 | 0.004667 | 7111924 | 0.272267 |
| Up | COPG2 | 43 | 0.004913 | 4775648 | 0.313082 |
| Up | FAT1 | 42 | 0.00404 | 5889734 | 0.276326 |
| Up | WWC1 | 42 | 0.003429 | 2836442 | 0.307386 |
| Up | IGFBP6 | 42 | 0.004231 | 3088344 | 0.288082 |
| Up | ITGB2 | 41 | 0.005357 | 4411146 | 0.303395 |
| Up | NUP188 | 41 | 0.003108 | 4907892 | 0.290617 |
| Up | COL1A2 | 40 | 0.005029 | 5099026 | 0.285883 |
| Up | HSPB8 | 40 | 0.003647 | 4517138 | 0.278775 |
| Up | SYNC | 40 | 0.004315 | 7896734 | 0.276295 |
| Up | PYGL | 39 | 0.003197 | 2471758 | 0.299901 |
| Up | PRSS23 | 39 | 0.004248 | 4905244 | 0.290966 |
| Up | PDLIM1 | 39 | 0.003659 | 2786872 | 0.299876 |
| Up | SFRP4 | 39 | 0.003767 | 2048128 | 0.298877 |
| Up | FAM98A | 37 | 0.00261 | 4955160 | 0.28916 |
| Up | RHOC | 37 | 0.004285 | 3441322 | 0.293599 |
| Up | TM4SF19 | 37 | 0.00657 | 2728698 | 0.298582 |
| Up | MARCKS | 35 | 0.003247 | 3052972 | 0.309508 |
| Up | DLGAP4 | 35 | 0.00253 | 4101184 | 0.292783 |
| Up | KLF4 | 34 | 0.002104 | 3387628 | 0.296499 |
| Up | FRMD6 | 34 | 0.003547 | 2173770 | 0.30067 |
| Up | INF2 | 34 | 0.001906 | 3741376 | 0.28604 |
| Up | NMT2 | 34 | 0.004925 | 5082380 | 0.285804 |
| Up | CYB561 | 34 | 0.004216 | 2892542 | 0.26327 |
| Up | PLTP | 33 | 0.003576 | 3913370 | 0.266437 |
| Up | GNG2 | 33 | 0.00334 | 8521644 | 0.258991 |
| Up | STMN2 | 33 | 0.002979 | 3380914 | 0.280173 |
| Up | RAB31 | 33 | 0.003188 | 4366610 | 0.279064 |
| Up | FAM129B | 31 | 0.00205 | 1963278 | 0.294946 |
| Up | LAMB2 | 31 | 0.002316 | 1445266 | 0.300434 |
| Up | S100A11 | 31 | 0.001135 | 1884242 | 0.294504 |
| Up | GNS | 31 | 0.003232 | 3132614 | 0.290048 |
| Up | FGR | 31 | 0.002564 | 1608758 | 0.306595 |
| Up | SNCG | 31 | 0.002583 | 1761424 | 0.285445 |
| Up | LHX6 | 30 | 0.004005 | 4295336 | 0.246849 |
| Up | MMP9 | 29 | 0.003533 | 1643986 | 0.276106 |
| Up | SCARB1 | 29 | 0.002413 | 3830600 | 0.280985 |
| Up | CCPG1 | 29 | 0.004637 | 5110776 | 0.259472 |
| Up | COL6A2 | 29 | 0.002815 | 3690848 | 0.252537 |
| Up | GRIN2B | 29 | 0.002302 | 1122488 | 0.285838 |
| Up | CRIP1 | 28 | 0.001512 | 1100488 | 0.275102 |
| Up | LOX | 28 | 0.003132 | 2234520 | 0.288769 |
| Up | THY1 | 28 | 0.001582 | 2195824 | 0.270061 |
| Up | CCND2 | 28 | 0.001204 | 1179300 | 0.283695 |
| Up | HP | 28 | 0.003646 | 2155794 | 0.295847 |
| Up | LAMA5 | 27 | 0.002587 | 1793790 | 0.277859 |
| Up | TNFAIP6 | 27 | 0.006221 | 4007336 | 0.269101 |
| Up | EPDR1 | 27 | 0.003089 | 1767128 | 0.292819 |
| Up | KCTD12 | 26 | 0.001742 | 1864764 | 0.281377 |
| Up | CRTAP | 26 | 0.001485 | 1239886 | 0.280562 |
| Up | NQO1 | 25 | 0.001997 | 3076618 | 0.283352 |
| Up | TMEM189 | 25 | 0.002319 | 1489396 | 0.29263 |
| Up | HSPA12A | 25 | 0.00251 | 2745446 | 0.270302 |
| Up | CCDC107 | 25 | 0.002939 | 2945106 | 0.271718 |
| Up | CDK14 | 25 | 0.002145 | 1096846 | 0.290978 |
| Up | CD68 | 25 | 0.002914 | 1383318 | 0.282131 |
| Up | PLXNA1 | 24 | 9.97E-04 | 1385474 | 0.270766 |
| Up | CSN1S1 | 24 | 0.003031 | 1574476 | 0.289355 |
| Up | TLE2 | 24 | 0.002913 | 4771720 | 0.250017 |
| Up | TNS3 | 24 | 0.001733 | 1610768 | 0.300832 |
| Up | ZMAT3 | 24 | 0.002518 | 3853494 | 0.282 |
| Up | CMTM3 | 24 | 0.003041 | 1808232 | 0.276495 |
| Up | FBLN2 | 23 | 0.001502 | 1075964 | 0.266359 |
| Up | COL6A1 | 23 | 0.001413 | 1973664 | 0.27114 |
| Up | S100A3 | 23 | 0.003098 | 2878998 | 0.266975 |
| Up | NABP1 | 23 | 0.00259 | 2546540 | 0.287888 |
| Up | TMEM54 | 23 | 0.003244 | 2905042 | 0.268504 |
| Up | SLC2A5 | 23 | 0.003375 | 3974418 | 0.245913 |
| Up | ELN | 22 | 0.002632 | 2145182 | 0.257094 |
| Up | LAMB3 | 22 | 0.001502 | 3046028 | 0.266681 |
| Up | SLIT2 | 22 | 0.00217 | 1500762 | 0.276895 |
| Up | IRF8 | 22 | 0.001595 | 2777354 | 0.276022 |
| Up | STK38L | 22 | 0.001104 | 2534630 | 0.285075 |
| Up | PLEKHO2 | 22 | 0.002217 | 2540732 | 0.280259 |
| Up | GPC1 | 21 | 0.002734 | 2130592 | 0.265163 |
| Up | PTGDS | 21 | 0.001473 | 2337746 | 0.283131 |
| Up | GRB14 | 21 | 0.001711 | 2329784 | 0.283242 |
| Up | KANK4 | 21 | 0.001599 | 1521196 | 0.263061 |
| Up | TYMP | 21 | 0.001517 | 1727486 | 0.26495 |
| Up | CYB561A3 | 20 | 0.002132 | 1166162 | 0.238198 |
| Up | HTRA1 | 20 | 0.002054 | 1885686 | 0.274614 |
| Up | B4GALT5 | 20 | 0.00169 | 2081630 | 0.25831 |
| Up | LCP1 | 20 | 9.58E-04 | 807902 | 0.287466 |
| Up | CPE | 20 | 0.001095 | 1580024 | 0.254127 |
| Up | KCNB1 | 20 | 0.00263 | 3492512 | 0.248759 |
| Up | OAF | 20 | 0.002196 | 1509446 | 0.24643 |
| Up | SYNPO2 | 20 | 0.0017 | 928102 | 0.313487 |
| Up | ENC1 | 20 | 0.001007 | 2393036 | 0.267545 |
| Up | DOCK6 | 20 | 0.002214 | 2355528 | 0.264247 |
| Up | STC2 | 19 | 0.001811 | 1655032 | 0.277233 |
| Up | MT2A | 19 | 0.002237 | 959090 | 0.268902 |
| Up | CTGF | 19 | 0.001764 | 1325662 | 0.294456 |
| Up | MYL9 | 19 | 0.001619 | 2835070 | 0.268912 |
| Up | SPARC | 18 | 8.80E-04 | 597658 | 0.277001 |
| Up | CLPTM1L | 18 | 0.001824 | 833924 | 0.285512 |
| Up | RND3 | 18 | 0.001204 | 1106966 | 0.265677 |
| Up | PDGFD | 18 | 0.001355 | 1414168 | 0.243393 |
| Up | CFH | 17 | 0.001731 | 2152476 | 0.272994 |
| Up | SKAP2 | 17 | 9.81E-04 | 1151386 | 0.283396 |
| Up | NRP2 | 17 | 9.07E-04 | 486396 | 0.251654 |
| Up | PTGFRN | 17 | 0.001198 | 1067934 | 0.269121 |
| Up | LUM | 17 | 0.001227 | 656762 | 0.25121 |
| Up | MSTO1 | 17 | 0.002208 | 3259790 | 0.257149 |
| Up | SULT1A4 | 17 | 6.90E-04 | 583300 | 0.273363 |
| Up | EGR2 | 17 | 0.00263 | 3566970 | 0.248682 |
| Up | HSD11B1 | 16 | 0.002065 | 1911100 | 0.262538 |
| Up | CLEC3B | 16 | 0.001399 | 1548088 | 0.268039 |
| Up | SPATA18 | 16 | 5.71E-04 | 672478 | 0.253799 |
| Up | LBP | 16 | 0.002333 | 1568590 | 0.268952 |
| Up | SLC15A3 | 15 | 4.37E-04 | 457084 | 0.256794 |
| Up | TNMD | 15 | 0.001379 | 1792380 | 0.231387 |
| Up | SULT1A3 | 15 | 6.90E-04 | 583300 | 0.273363 |
| Up | EGFL6 | 15 | 0.001449 | 606972 | 0.282801 |
| Up | TIMP1 | 14 | 9.63E-04 | 966968 | 0.260048 |
| Up | PCDH9 | 14 | 0.00128 | 1242150 | 0.259472 |
| Up | SCIN | 14 | 0.001524 | 2668388 | 0.272329 |
| Up | PPFIBP2 | 13 | 5.69E-04 | 812444 | 0.271941 |
| Up | CFB | 13 | 0.001023 | 696242 | 0.267703 |
| Up | MGST1 | 13 | 0.00132 | 678514 | 0.284306 |
| Up | FAR2 | 13 | 8.79E-04 | 732968 | 0.218776 |
| Up | SUSD1 | 13 | 0.001025 | 1337044 | 0.270887 |
| Up | TPGS2 | 13 | 0.001458 | 2285296 | 0.267575 |
| Up | TDP1 | 13 | 9.31E-04 | 1584550 | 0.265415 |
| Up | CRABP2 | 13 | 8.82E-04 | 942492 | 0.267536 |
| Up | OSTM1 | 13 | 7.99E-04 | 999584 | 0.26442 |
| Up | TMED3 | 13 | 0.001768 | 1105754 | 0.265813 |
| Up | COL3A1 | 12 | 2.61E-04 | 160032 | 0.26087 |
| Up | CXCL12 | 12 | 0.00126 | 1131608 | 0.249862 |
| Up | ALCAM | 12 | 7.48E-04 | 562268 | 0.281934 |
| Up | PLBD2 | 12 | 0.001267 | 1342160 | 0.26394 |
| Up | VLDLR | 12 | 9.88E-04 | 542830 | 0.238456 |
| Up | RARRES3 | 12 | 9.30E-04 | 856238 | 0.279161 |
| Up | CLIP3 | 12 | 6.70E-04 | 1115216 | 0.261593 |
| Up | TYROBP | 12 | 0.002505 | 2860066 | 0.223185 |
| Up | C1S | 11 | 6.79E-04 | 608832 | 0.25209 |
| Up | APBB1IP | 11 | 9.33E-04 | 1638836 | 0.25223 |
| Up | AKR1C3 | 11 | 0.001423 | 2534148 | 0.227205 |
| Up | GSTM5 | 11 | 8.52E-04 | 1238616 | 0.242426 |
| Up | PMEPA1 | 11 | 4.35E-04 | 769218 | 0.259983 |
| Up | CES1 | 11 | 6.23E-04 | 347800 | 0.256233 |
| Up | CST3 | 10 | 3.63E-04 | 602294 | 0.240246 |
| Up | C1R | 10 | 7.52E-04 | 640506 | 0.24426 |
| Up | TPST2 | 10 | 0.001334 | 791042 | 0.240063 |
| Up | IL1RN | 10 | 8.27E-04 | 447858 | 0.264161 |
| Up | FAM171A1 | 10 | 7.80E-04 | 810132 | 0.259472 |
| Up | WISP2 | 10 | 0.00128 | 834384 | 0.252994 |
| Up | BAMBI | 10 | 5.81E-04 | 1266400 | 0.209461 |
| Up | CYR61 | 10 | 4.24E-04 | 263992 | 0.274127 |
| Up | TRIM6 | 10 | 4.51E-04 | 534944 | 0.26087 |
| Up | ALPL | 10 | 5.55E-04 | 721174 | 0.261057 |
| Up | ITIH5 | 10 | 6.43E-04 | 776104 | 0.254243 |
| Up | LRRC32 | 10 | 0.001144 | 585708 | 0.29736 |
| Up | RAB7B | 10 | 7.26E-04 | 844758 | 0.254278 |
| Up | TNFRSF25 | 9 | 2.85E-04 | 685356 | 0.24732 |
| Up | MRAP | 9 | 7.09E-05 | 25240 | 0.224052 |
| Up | ABCC3 | 9 | 8.71E-04 | 1216854 | 0.26163 |
| Up | SULT1A2 | 9 | 6.57E-04 | 314992 | 0.251924 |
| Up | TMEM98 | 9 | 2.61E-04 | 155616 | 0.243727 |
| Up | OXT | 8 | 2.82E-04 | 94820 | 0.270736 |
| Up | ALDH1A3 | 8 | 4.74E-04 | 503556 | 0.264468 |
| Up | FRZB | 8 | 9.85E-04 | 1157822 | 0.241556 |
| Up | NRCAM | 8 | 5.47E-04 | 264746 | 0.26952 |
| Up | MXRA7 | 8 | 6.15E-04 | 756206 | 0.27286 |
| Up | CCL3L3 | 7 | 5.89E-04 | 701756 | 0.221863 |
| Up | GALNT16 | 7 | 8.61E-04 | 688996 | 0.20576 |
| Up | MFGE8 | 7 | 6.10E-05 | 57244 | 0.275666 |
| Up | NANOS3 | 7 | 0.00111 | 1372154 | 0.223116 |
| Up | SLC43A2 | 7 | 2.34E-04 | 412138 | 0.263023 |
| Up | SH3BGRL3 | 7 | 5.99E-04 | 384954 | 0.294528 |
| Up | GLIPR2 | 7 | 4.84E-04 | 529920 | 0.264719 |
| Up | SLC35G1 | 7 | 4.45E-04 | 474316 | 0.250595 |
| Up | AIFM2 | 7 | 4.98E-04 | 235562 | 0.277424 |
| Up | GPR137B | 7 | 8.43E-04 | 898792 | 0.225708 |
| Up | MSC | 7 | 6.42E-05 | 82488 | 0.258337 |
| Up | CHST3 | 6 | 1.06E-04 | 160442 | 0.256785 |
| Up | CSF2RA | 6 | 8.28E-04 | 886838 | 0.247733 |
| Up | HNMT | 6 | 3.42E-05 | 17242 | 0.256052 |
| Up | GPNMB | 6 | 3.16E-04 | 277798 | 0.209758 |
| Up | FMOD | 6 | 2.06E-04 | 211588 | 0.256667 |
| Up | LHCGR | 6 | 2.88E-04 | 385926 | 0.236786 |
| Up | AOX1 | 6 | 3.09E-04 | 272424 | 0.24166 |
| Up | PLAC9 | 6 | 6.27E-04 | 222890 | 0.244531 |
| Up | SVEP1 | 6 | 1.77E-04 | 275960 | 0.229465 |
| Up | TNFRSF12A | 6 | 4.39E-04 | 734164 | 0.234017 |
| Up | ARHGEF35 | 6 | 6.12E-04 | 505012 | 0.261838 |
| Up | COL12A1 | 6 | 5.77E-04 | 302930 | 0.236917 |
| Up | ANO3 | 6 | 3.43E-04 | 145774 | 0.283884 |
| Up | CYP11A1 | 6 | 4.21E-04 | 468402 | 0.22961 |
| Up | INHBB | 6 | 8.26E-04 | 1475316 | 0.197768 |
| Up | MFAP4 | 5 | 1.82E-04 | 253934 | 0.269101 |
| Up | PTGER4 | 5 | 8.66E-05 | 176032 | 0.251593 |
| Up | EMX2 | 5 | 8.39E-04 | 557038 | 0.207446 |
| Up | SGCE | 5 | 8.61E-05 | 130332 | 0.240437 |
| Up | COL15A1 | 5 | 3.88E-05 | 52870 | 0.252441 |
| Up | SDSL | 5 | 3.32E-06 | 14564 | 0.243034 |
| Up | MEDAG | 5 | 5.76E-04 | 526934 | 0.245531 |
| Up | DHDDS | 5 | 3.63E-04 | 153640 | 0.237443 |
| Up | CXCL10 | 4 | 1.67E-05 | 16262 | 0.195302 |
| Up | CCDC80 | 4 | 1.46E-05 | 25932 | 0.2422 |
| Up | BHMT2 | 4 | 2.82E-04 | 426448 | 0.252204 |
| Up | RSPO3 | 4 | 5.54E-04 | 508130 | 0.214551 |
| Up | ELMOD3 | 4 | 5.58E-04 | 492186 | 0.252739 |
| Up | FCGBP | 4 | 3.13E-05 | 39280 | 0.239952 |
| Up | MYEOV | 4 | 5.54E-04 | 423866 | 0.216025 |
| Up | CALB2 | 4 | 5.57E-06 | 11274 | 0.217571 |
| Up | CLIC6 | 4 | 8.26E-04 | 476328 | 0.205133 |
| Up | SLC22A12 | 4 | 3.13E-04 | 225200 | 0.230308 |
| Up | SAA2 | 3 | 2.46E-04 | 227126 | 0.206321 |
| Up | NPR3 | 3 | 5.50E-04 | 508058 | 0.198411 |
| Up | LRRN3 | 3 | 4.02E-05 | 40314 | 0.248776 |
| Up | FSTL3 | 3 | 5.50E-04 | 207682 | 0.203296 |
| Up | MN1 | 3 | 5.03E-05 | 16738 | 0.263547 |
| Up | NPY5R | 3 | 5.50E-04 | 624054 | 0.23441 |
| Up | LEP | 3 | 2.00E-05 | 6786 | 0.240079 |
| Up | TNFRSF11B | 3 | 5.50E-04 | 350406 | 0.174996 |
| Up | FGF11 | 3 | 5.50E-04 | 761782 | 0.20555 |
| Up | ESPNL | 3 | 1.27E-06 | 498 | 0.203478 |
| Up | CLCA2 | 3 | 3.05E-04 | 367670 | 0.228075 |
| Up | PEMT | 3 | 1.59E-04 | 62928 | 0.243646 |
| Up | IRX2 | 3 | 2.77E-04 | 309966 | 0.21852 |
| Up | STEAP1 | 3 | 4.53E-04 | 352508 | 0.215429 |
| Up | ZNF541 | 3 | 2.77E-04 | 274356 | 0.247572 |
| Up | SOBP | 3 | 2.77E-04 | 316828 | 0.222188 |
| Up | C1RL | 3 | 1.77E-04 | 66534 | 0.262557 |
| Up | COL16A1 | 2 | 2.04E-05 | 16970 | 0.239115 |
| Up | PLA2G7 | 2 | 2.75E-04 | 335702 | 0.24376 |
| Up | SAA1 | 2 | 2.75E-04 | 148902 | 0.195675 |
| Up | CTHRC1 | 2 | 6.09E-06 | 4436 | 0.211849 |
| Up | PRG4 | 2 | 2.46E-06 | 14846 | 0.264835 |
| Up | COL5A2 | 2 | 6.90E-07 | 1504 | 0.240955 |
| Up | MXRA5 | 2 | 1.03E-06 | 2100 | 0.230945 |
| Up | TRAM2 | 2 | 3.17E-05 | 31874 | 0.246623 |
| Up | SLC46A3 | 2 | 1.00E-05 | 7956 | 0.229255 |
| Up | CHI3L2 | 2 | 3.90E-06 | 1466 | 0.215327 |
| Up | SAA4 | 2 | 4.85E-05 | 50948 | 0.190364 |
| Up | SEMA3G | 2 | 2.75E-04 | 257800 | 0.19568 |
| Up | ANGPT1 | 2 | 2.61E-06 | 808 | 0.214406 |
| Up | MGAT3 | 2 | 3.91E-05 | 29366 | 0.244383 |
| Up | ANKDD1A | 2 | 9.60E-07 | 1688 | 0.2118 |
| Up | CD248 | 2 | 6.52E-06 | 10130 | 0.239873 |
| Up | HOXB8 | 2 | 4.46E-06 | 1564 | 0.202876 |
| Up | AQP11 | 1 | 0 | 0 | 0.203752 |
| Up | STEAP2 | 1 | 0 | 0 | 1 |
| Up | ZCCHC24 | 1 | 0 | 0 | 0.195664 |
| Up | ZNF404 | 1 | 0 | 0 | 0.216269 |
| Up | CADM3 | 1 | 0 | 0 | 0.193111 |
| Up | ADAMTS5 | 1 | 0 | 0 | 0.183304 |
| Up | CLDN15 | 1 | 0 | 0 | 0.189782 |
| Up | FILIP1L | 1 | 0 | 0 | 0.203216 |
| Up | ERV3-1 | 1 | 0 | 0 | 0.205306 |
| Up | SRPX2 | 1 | 0 | 0 | 0.200165 |
| Up | COL11A1 | 1 | 0 | 0 | 1 |
| Up | DSEL | 1 | 0 | 0 | 0.24154 |
| Up | CD52 | 1 | 0 | 0 | 0.213939 |
| Down | ESR1 | 806 | 0.153485 | 2.1E+08 | 0.391884 |
| Down | FBL | 381 | 0.043577 | 81882548 | 0.346337 |
| Down | CSNK2B | 292 | 0.043492 | 62952016 | 0.346503 |
| Down | ARRB1 | 284 | 0.043293 | 53941490 | 0.351245 |
| Down | PCM1 | 259 | 0.045751 | 77483406 | 0.325315 |
| Down | RPS2 | 258 | 0.021334 | 29889924 | 0.362321 |
| Down | NR3C1 | 253 | 0.03159 | 46690102 | 0.34422 |
| Down | ADRB2 | 250 | 0.03968 | 53808532 | 0.340109 |
| Down | RAD21 | 248 | 0.03088 | 38413802 | 0.337972 |
| Down | RPS3 | 245 | 0.017178 | 35272712 | 0.36097 |
| Down | SIRT1 | 244 | 0.034146 | 31338754 | 0.355876 |
| Down | PTEN | 241 | 0.037893 | 32252050 | 0.360594 |
| Down | RPL14 | 226 | 0.013916 | 21099462 | 0.354002 |
| Down | CSNK2A2 | 222 | 0.028742 | 33637672 | 0.343584 |
| Down | RBBP4 | 207 | 0.023381 | 32706132 | 0.348164 |
| Down | SIN3A | 205 | 0.025789 | 23294856 | 0.343942 |
| Down | RPL18 | 200 | 0.010425 | 18789086 | 0.348548 |
| Down | EEF1G | 198 | 0.024334 | 40864084 | 0.340859 |
| Down | RPL18A | 180 | 0.009178 | 15465286 | 0.337579 |
| Down | EEF1D | 170 | 0.017304 | 24337902 | 0.342257 |
| Down | KHDRBS1 | 169 | 0.021941 | 22978510 | 0.341725 |
| Down | TSC22D1 | 165 | 0.023146 | 19501674 | 0.327648 |
| Down | ZBTB16 | 162 | 0.0213 | 20359632 | 0.339647 |
| Down | RPS18 | 160 | 0.006517 | 12825590 | 0.353588 |
| Down | FKBP5 | 159 | 0.025477 | 16402018 | 0.340811 |
| Down | RPS9 | 156 | 0.008163 | 14334452 | 0.355127 |
| Down | RBMX | 154 | 0.015786 | 19978510 | 0.343454 |
| Down | SRSF3 | 147 | 0.012702 | 20302456 | 0.34695 |
| Down | CNBP | 143 | 0.008966 | 10463900 | 0.329148 |
| Down | GMCL1 | 142 | 0.026139 | 13929296 | 0.325213 |
| Down | DCAF7 | 133 | 0.015646 | 19452348 | 0.330181 |
| Down | RPS27 | 130 | 0.006414 | 10432460 | 0.333287 |
| Down | ANG | 125 | 0.012754 | 12253912 | 0.329746 |
| Down | RPS15A | 122 | 0.003647 | 7958980 | 0.346387 |
| Down | RPL21 | 119 | 0.003241 | 7186482 | 0.343114 |
| Down | MCM6 | 115 | 0.011128 | 16694928 | 0.318021 |
| Down | PHGDH | 114 | 0.0116 | 16787514 | 0.340651 |
| Down | BHLHE40 | 111 | 0.016058 | 12746680 | 0.327767 |
| Down | SRSF7 | 110 | 0.005523 | 11199958 | 0.334546 |
| Down | EIF3H | 100 | 0.007885 | 9251336 | 0.3279 |
| Down | PCBD1 | 99 | 0.013565 | 10286996 | 0.325169 |
| Down | ERRFI1 | 98 | 0.014097 | 15392316 | 0.321384 |
| Down | TRIM33 | 97 | 0.009891 | 16723318 | 0.319153 |
| Down | ZNF581 | 95 | 0.01075 | 17787620 | 0.300492 |
| Down | SAFB | 94 | 0.006963 | 9017284 | 0.340875 |
| Down | CDKN1B | 94 | 0.00783 | 14212380 | 0.324008 |
| Down | RPL35 | 94 | 0.002448 | 5082406 | 0.323994 |
| Down | RPL17 | 91 | 0.003522 | 6092462 | 0.336593 |
| Down | MAP3K5 | 91 | 0.008997 | 9878956 | 0.322655 |
| Down | EIF4B | 89 | 0.006598 | 9945562 | 0.327811 |
| Down | MED28 | 85 | 0.004511 | 6219300 | 0.311861 |
| Down | UBE2W | 82 | 0.010803 | 7628448 | 0.314508 |
| Down | RPS17 | 82 | 0.002298 | 4799800 | 0.332905 |
| Down | RPS12 | 80 | 0.002825 | 4036378 | 0.339934 |
| Down | ACACA | 80 | 0.009068 | 7691876 | 0.325782 |
| Down | CKB | 78 | 0.006241 | 16771888 | 0.309839 |
| Down | GTF3C3 | 74 | 0.007225 | 7434672 | 0.321883 |
| Down | SLC3A2 | 73 | 0.009036 | 10552728 | 0.326294 |
| Down | STK24 | 72 | 0.007434 | 10153518 | 0.312344 |
| Down | PLSCR4 | 72 | 0.010452 | 7631318 | 0.296772 |
| Down | POLR3C | 68 | 0.006634 | 7596830 | 0.30519 |
| Down | FBXO11 | 68 | 0.008173 | 17787740 | 0.308326 |
| Down | RBL2 | 67 | 0.005282 | 4511324 | 0.319729 |
| Down | FOXO3 | 67 | 0.004241 | 7009168 | 0.320378 |
| Down | ORC2 | 66 | 0.004554 | 4837818 | 0.305152 |
| Down | CBS | 66 | 0.006855 | 10225996 | 0.306349 |
| Down | HSD17B11 | 65 | 0.012449 | 11836966 | 0.310927 |
| Down | POLR1D | 64 | 0.00273 | 1440872 | 0.29313 |
| Down | H1FX | 64 | 0.002945 | 6401706 | 0.319223 |
| Down | UBR2 | 63 | 0.008831 | 13796534 | 0.289738 |
| Down | INTS6 | 63 | 0.005834 | 15502722 | 0.296311 |
| Down | CCDC59 | 62 | 0.004589 | 3595682 | 0.314781 |
| Down | TRIM24 | 62 | 0.006498 | 4516452 | 0.324515 |
| Down | GLYCTK | 62 | 0.00675 | 11693046 | 0.283838 |
| Down | NASP | 60 | 0.004695 | 4623040 | 0.316744 |
| Down | PMP22 | 60 | 0.011787 | 10774698 | 0.287604 |
| Down | FOXO1 | 60 | 0.004278 | 3088194 | 0.319125 |
| Down | RPL29 | 60 | 0.001898 | 2789638 | 0.330301 |
| Down | HDAC9 | 60 | 0.00473 | 3277096 | 0.320958 |
| Down | NRIP1 | 59 | 0.004763 | 3915702 | 0.331371 |
| Down | DPF2 | 59 | 0.004275 | 10395394 | 0.305408 |
| Down | GJA1 | 57 | 0.007603 | 9578982 | 0.29055 |
| Down | MED16 | 56 | 0.002612 | 1962778 | 0.316648 |
| Down | LEMD3 | 56 | 0.007232 | 9603698 | 0.301829 |
| Down | AIP | 56 | 0.006796 | 11292940 | 0.298577 |
| Down | FABP5 | 55 | 0.004222 | 5751334 | 0.308588 |
| Down | GID8 | 54 | 0.006384 | 5854498 | 0.309693 |
| Down | RPS6KA5 | 53 | 0.005533 | 4215266 | 0.320053 |
| Down | SYT17 | 52 | 0.0054 | 4982314 | 0.307829 |
| Down | MED6 | 52 | 0.001798 | 1731700 | 0.31349 |
| Down | USP13 | 52 | 0.004671 | 3189134 | 0.31589 |
| Down | SRSF4 | 51 | 0.001223 | 4103844 | 0.29794 |
| Down | PPP1R16A | 51 | 0.005746 | 8058742 | 0.284528 |
| Down | MAPRE2 | 50 | 0.003825 | 4616696 | 0.314468 |
| Down | SMIM3 | 50 | 0.008337 | 3707366 | 0.254227 |
| Down | RSBN1 | 49 | 0.003391 | 3832346 | 0.300418 |
| Down | HMGB2 | 49 | 0.00357 | 6132900 | 0.324501 |
| Down | TMOD1 | 48 | 0.003984 | 8904450 | 0.296299 |
| Down | MED30 | 48 | 0.001878 | 1464014 | 0.312626 |
| Down | CTH | 47 | 0.006011 | 3830276 | 0.296348 |
| Down | HSD17B12 | 47 | 0.005085 | 5578098 | 0.313937 |
| Down | RGS3 | 47 | 0.005772 | 3327954 | 0.303786 |
| Down | TSC22D3 | 47 | 0.005191 | 6455444 | 0.317965 |
| Down | FBP1 | 46 | 0.005464 | 7243238 | 0.290526 |
| Down | HERC5 | 46 | 0.002437 | 2306250 | 0.315383 |
| Down | MAPK10 | 46 | 0.004248 | 3264596 | 0.315986 |
| Down | MLX | 46 | 0.005762 | 3878434 | 0.314863 |
| Down | RBM15 | 45 | 0.002883 | 3510776 | 0.316372 |
| Down | GINS3 | 45 | 0.004967 | 3943546 | 0.295371 |
| Down | POLR1E | 45 | 0.00356 | 2068300 | 0.309074 |
| Down | MRPL55 | 45 | 0.001429 | 1326326 | 0.296215 |
| Down | TWIST1 | 44 | 0.003116 | 2544250 | 0.307347 |
| Down | MKNK2 | 44 | 0.002816 | 4837732 | 0.305216 |
| Down | STAG2 | 44 | 0.00376 | 3789932 | 0.303088 |
| Down | LDHD | 44 | 0.006194 | 16854336 | 0.276069 |
| Down | SMIM1 | 44 | 0.004525 | 9485444 | 0.238897 |
| Down | CASQ2 | 44 | 0.004898 | 4308174 | 0.281473 |
| Down | SCP2 | 43 | 0.005135 | 6506708 | 0.296869 |
| Down | PHLPP1 | 43 | 0.004994 | 3038530 | 0.311848 |
| Down | TRIP4 | 42 | 0.001657 | 1529960 | 0.313815 |
| Down | GMPS | 42 | 0.003893 | 7262370 | 0.309693 |
| Down | SHMT1 | 42 | 0.003936 | 8051056 | 0.287866 |
| Down | TXNIP | 42 | 0.003899 | 3198328 | 0.311567 |
| Down | SLC27A2 | 41 | 0.004286 | 7953682 | 0.281615 |
| Down | PLD1 | 41 | 0.004575 | 8671384 | 0.277281 |
| Down | STARD13 | 41 | 0.004088 | 7680918 | 0.28168 |
| Down | H1F0 | 39 | 0.002026 | 5049202 | 0.307725 |
| Down | RPS29 | 39 | 0.00166 | 1696418 | 0.30281 |
| Down | KAT8 | 39 | 0.002294 | 2594188 | 0.308562 |
| Down | PPP3R1 | 39 | 0.00613 | 3752894 | 0.303013 |
| Down | SIVA1 | 39 | 0.004978 | 8423944 | 0.300654 |
| Down | NDRG4 | 39 | 0.005926 | 3321224 | 0.2663 |
| Down | GLUL | 38 | 0.003974 | 7950262 | 0.297806 |
| Down | YPEL5 | 38 | 0.003258 | 3190842 | 0.303418 |
| Down | NDUFV3 | 38 | 0.005051 | 10700788 | 0.277578 |
| Down | TMEM243 | 38 | 0.003169 | 7642644 | 0.240719 |
| Down | AHCTF1 | 36 | 0.003267 | 9927400 | 0.285996 |
| Down | MPDZ | 36 | 0.004815 | 7322830 | 0.289785 |
| Down | PELI2 | 36 | 0.004349 | 8786880 | 0.283738 |
| Down | EIF2A | 35 | 0.002235 | 4455856 | 0.301228 |
| Down | RERE | 35 | 0.002532 | 3260444 | 0.288495 |
| Down | FAM107A | 35 | 0.001685 | 2737530 | 0.280538 |
| Down | ADD3 | 34 | 0.002352 | 3903388 | 0.29566 |
| Down | ACVR1 | 34 | 0.002812 | 1540502 | 0.281615 |
| Down | DTNA | 34 | 0.0046 | 6493716 | 0.268238 |
| Down | BCKDHA | 33 | 0.004981 | 3209648 | 0.297989 |
| Down | GPHN | 33 | 0.003321 | 1994352 | 0.29105 |
| Down | ORMDL3 | 33 | 0.005211 | 6879928 | 0.295263 |
| Down | ANK2 | 32 | 0.003754 | 5707286 | 0.299847 |
| Down | LAPTM4B | 32 | 0.004378 | 5216260 | 0.279943 |
| Down | CMTM7 | 32 | 0.003893 | 6019606 | 0.23971 |
| Down | ELP2 | 31 | 0.002629 | 4968302 | 0.277706 |
| Down | LETMD1 | 31 | 0.003704 | 7124402 | 0.291132 |
| Down | N4BP2L2 | 30 | 0.00334 | 4389434 | 0.264371 |
| Down | ELK1 | 30 | 0.002079 | 2769152 | 0.300667 |
| Down | NR2F1 | 29 | 0.002065 | 959324 | 0.312505 |
| Down | NR4A2 | 29 | 0.002998 | 2829956 | 0.296215 |
| Down | MACROD1 | 29 | 0.001235 | 3532516 | 0.281375 |
| Down | TMEM100 | 29 | 0.003454 | 6768494 | 0.24397 |
| Down | RFX1 | 28 | 0.002666 | 2779366 | 0.290352 |
| Down | TGIF1 | 28 | 0.001931 | 2216518 | 0.309048 |
| Down | RNF38 | 28 | 0.001869 | 2323162 | 0.295467 |
| Down | EEF2K | 27 | 0.001709 | 1732296 | 0.300754 |
| Down | CENPV | 27 | 7.64E-04 | 916218 | 0.300791 |
| Down | GPD1L | 27 | 0.0038 | 2065944 | 0.287764 |
| Down | RTN1 | 27 | 0.003472 | 3693402 | 0.24963 |
| Down | RNF144A | 26 | 0.001768 | 2193532 | 0.263097 |
| Down | PCCA | 26 | 0.002139 | 1140242 | 0.295756 |
| Down | AZGP1 | 26 | 0.001528 | 3469730 | 0.282975 |
| Down | CISH | 26 | 0.002792 | 5275460 | 0.291085 |
| Down | CDS2 | 26 | 0.002017 | 4473790 | 0.243055 |
| Down | UBTD1 | 26 | 0.003129 | 5391322 | 0.274297 |
| Down | CD55 | 26 | 0.002437 | 2011446 | 0.272956 |
| Down | PHIP | 25 | 5.08E-04 | 1172608 | 0.287912 |
| Down | CD36 | 25 | 0.003914 | 7406080 | 0.276006 |
| Down | S100A1 | 25 | 0.002661 | 2518130 | 0.289058 |
| Down | REV1 | 25 | 0.001251 | 2934360 | 0.289912 |
| Down | MTUS1 | 25 | 6.89E-04 | 974254 | 0.279404 |
| Down | PAPSS1 | 24 | 0.002838 | 1647244 | 0.29396 |
| Down | RSL24D1 | 24 | 3.60E-04 | 545300 | 0.29578 |
| Down | BDH1 | 24 | 0.004104 | 2618626 | 0.283285 |
| Down | PER2 | 24 | 0.001282 | 946214 | 0.309997 |
| Down | GADD45B | 24 | 0.002817 | 1193404 | 0.27519 |
| Down | RNF125 | 23 | 0.001332 | 1162624 | 0.296396 |
| Down | SOCS2 | 23 | 0.001435 | 940792 | 0.281702 |
| Down | SPSB1 | 23 | 0.001434 | 2592614 | 0.297867 |
| Down | CLDND1 | 22 | 0.003682 | 3938544 | 0.227122 |
| Down | LDHC | 22 | 0.001828 | 1166582 | 0.280019 |
| Down | MAT2B | 22 | 0.001968 | 1498338 | 0.307243 |
| Down | SPSB3 | 21 | 0.002693 | 1276008 | 0.293284 |
| Down | FAM207A | 21 | 9.49E-04 | 884202 | 0.279835 |
| Down | NDFIP2 | 21 | 0.002063 | 1524792 | 0.302357 |
| Down | LSS | 20 | 0.00202 | 2696762 | 0.281954 |
| Down | FBXO46 | 20 | 9.35E-04 | 2293462 | 0.275263 |
| Down | RHOT1 | 20 | 0.001825 | 2280866 | 0.282942 |
| Down | GPC3 | 20 | 0.002147 | 2749372 | 0.254762 |
| Down | LRRCC1 | 20 | 0.002064 | 854052 | 0.274826 |
| Down | AUTS2 | 20 | 9.02E-04 | 504644 | 0.298406 |
| Down | ZC2HC1A | 20 | 8.63E-04 | 1221736 | 0.296784 |
| Down | WNT11 | 20 | 0.001522 | 5218644 | 0.229389 |
| Down | EYA1 | 20 | 0.001835 | 2780896 | 0.275849 |
| Down | IGF1 | 19 | 0.001837 | 818638 | 0.268694 |
| Down | CTSC | 19 | 0.001819 | 3214564 | 0.267329 |
| Down | NBEA | 19 | 0.001923 | 1417408 | 0.27439 |
| Down | ALDH6A1 | 19 | 0.00243 | 3499248 | 0.246982 |
| Down | FGFRL1 | 19 | 0.001946 | 3042038 | 0.258913 |
| Down | LACTB2 | 19 | 0.002624 | 4365370 | 0.250026 |
| Down | ACACB | 18 | 0.00102 | 942534 | 0.30749 |
| Down | FADS1 | 18 | 0.002153 | 2374440 | 0.235818 |
| Down | ZFAND5 | 18 | 0.001584 | 2970634 | 0.287752 |
| Down | HOXA10 | 18 | 8.32E-04 | 1820868 | 0.273049 |
| Down | SLC25A23 | 18 | 0.003186 | 2107154 | 0.292233 |
| Down | PXMP2 | 18 | 0.001454 | 638106 | 0.254905 |
| Down | BTG2 | 18 | 0.001432 | 2446066 | 0.273398 |
| Down | HDDC3 | 18 | 0.00212 | 2271936 | 0.264207 |
| Down | ELF2 | 17 | 0.00103 | 1487840 | 0.297599 |
| Down | SNTB1 | 17 | 0.001109 | 1816642 | 0.234311 |
| Down | ZHX2 | 17 | 0.001792 | 2313992 | 0.272823 |
| Down | OSBPL1A | 17 | 8.03E-04 | 1264296 | 0.274732 |
| Down | AZIN1 | 17 | 0.002061 | 845410 | 0.287172 |
| Down | AGT | 17 | 0.001628 | 2370886 | 0.290294 |
| Down | BLVRB | 16 | 0.001054 | 1899696 | 0.266154 |
| Down | PFKFB3 | 16 | 7.87E-04 | 486422 | 0.305113 |
| Down | RTN2 | 16 | 0.001709 | 1185784 | 0.252319 |
| Down | CCNB1IP1 | 16 | 7.78E-04 | 750980 | 0.293984 |
| Down | PDLIM3 | 15 | 0.001191 | 1801652 | 0.250968 |
| Down | MIS18BP1 | 15 | 0.002176 | 783488 | 0.296553 |
| Down | RORB | 15 | 9.28E-04 | 516190 | 0.278334 |
| Down | PMM1 | 15 | 0.001477 | 1129998 | 0.249236 |
| Down | BCAT2 | 15 | 0.001308 | 1601362 | 0.275222 |
| Down | TBX15 | 15 | 0.001199 | 1804722 | 0.241335 |
| Down | CHN2 | 14 | 0.001172 | 1488748 | 0.258535 |
| Down | GCHFR | 14 | 0.001199 | 1748870 | 0.272148 |
| Down | MATN2 | 14 | 0.001676 | 2457288 | 0.229636 |
| Down | SERTAD4 | 14 | 0.00115 | 1587264 | 0.289484 |
| Down | CCNF | 14 | 5.90E-04 | 950744 | 0.272536 |
| Down | TMLHE | 14 | 7.34E-04 | 588780 | 0.285422 |
| Down | NACAD | 14 | 0.0013 | 1098172 | 0.288095 |
| Down | PCYT2 | 14 | 0.001419 | 1597954 | 0.257765 |
| Down | C5 | 14 | 0.002009 | 3259876 | 0.225088 |
| Down | ECI1 | 13 | 8.42E-04 | 694090 | 0.286153 |
| Down | ARHGEF3 | 13 | 4.02E-04 | 892554 | 0.275483 |
| Down | FOXN2 | 13 | 0.001323 | 710658 | 0.293533 |
| Down | RFX2 | 13 | 0.00122 | 697206 | 0.250353 |
| Down | BTG3 | 13 | 8.16E-04 | 1023508 | 0.264323 |
| Down | SC5D | 12 | 0.001169 | 1802206 | 0.205851 |
| Down | PHF3 | 12 | 4.47E-04 | 280086 | 0.291869 |
| Down | EIF4EBP2 | 12 | 5.49E-04 | 527474 | 0.279147 |
| Down | CNRIP1 | 12 | 0.001496 | 1776000 | 0.258388 |
| Down | ZDHHC11 | 12 | 8.57E-04 | 1377474 | 0.234728 |
| Down | CDH23 | 11 | 0.0014 | 2519498 | 0.242649 |
| Down | CCNG2 | 11 | 9.91E-04 | 1771946 | 0.255013 |
| Down | NET1 | 11 | 6.54E-04 | 395582 | 0.286537 |
| Down | TTC32 | 11 | 8.95E-04 | 1167886 | 0.251716 |
| Down | DBP | 11 | 0.001232 | 1420266 | 0.250026 |
| Down | HYI | 11 | 1.59E-04 | 237420 | 0.25304 |
| Down | PER3 | 11 | 7.84E-04 | 578572 | 0.256572 |
| Down | MOCS1 | 10 | 0.002204 | 4112338 | 0.213425 |
| Down | IL13RA1 | 10 | 0.001567 | 1617998 | 0.261449 |
| Down | ZNF394 | 10 | 9.41E-04 | 1269642 | 0.24557 |
| Down | CSRNP1 | 10 | 7.20E-04 | 882450 | 0.280181 |
| Down | AASS | 10 | 3.71E-04 | 610398 | 0.269382 |
| Down | ADNP2 | 10 | 5.77E-04 | 864076 | 0.283971 |
| Down | RFTN2 | 10 | 0.001209 | 2171452 | 0.231739 |
| Down | RAI2 | 10 | 7.37E-04 | 851334 | 0.268814 |
| Down | CAB39L | 10 | 0.001142 | 1670150 | 0.243414 |
| Down | PRUNE2 | 9 | 3.27E-04 | 759154 | 0.260885 |
| Down | THYN1 | 9 | 5.05E-04 | 704092 | 0.285523 |
| Down | PLCL2 | 9 | 0.00111 | 3025490 | 0.25565 |
| Down | ABTB1 | 9 | 4.22E-04 | 232970 | 0.282843 |
| Down | EFNA1 | 9 | 0.001128 | 792906 | 0.268555 |
| Down | SPATA7 | 9 | 6.10E-04 | 551960 | 0.275545 |
| Down | MKX | 9 | 8.77E-05 | 153692 | 0.292587 |
| Down | CD302 | 9 | 5.79E-05 | 82832 | 0.208678 |
| Down | STOX1 | 9 | 6.94E-04 | 829352 | 0.242901 |
| Down | DAPK2 | 9 | 0.001145 | 1532140 | 0.255049 |
| Down | GSDMB | 8 | 8.86E-04 | 639468 | 0.264602 |
| Down | MSRB1 | 8 | 5.94E-04 | 1094210 | 0.253712 |
| Down | PPP1R1B | 8 | 3.53E-04 | 586750 | 0.260501 |
| Down | FAM214A | 8 | 1.01E-04 | 112368 | 0.250595 |
| Down | TTLL7 | 8 | 1.93E-04 | 116868 | 0.28749 |
| Down | FAM13A | 8 | 8.98E-04 | 849488 | 0.248929 |
| Down | CABLES1 | 7 | 4.14E-04 | 563884 | 0.267773 |
| Down | NAALAD2 | 7 | 4.58E-04 | 222398 | 0.216867 |
| Down | ADH1B | 7 | 0.00125 | 1783716 | 0.261204 |
| Down | CALCRL | 7 | 0.00111 | 437762 | 0.187489 |
| Down | ZNF395 | 7 | 4.70E-05 | 69798 | 0.28017 |
| Down | PDK4 | 7 | 8.39E-04 | 1826048 | 0.236833 |
| Down | FAM126B | 7 | 3.34E-04 | 570254 | 0.284439 |
| Down | ST3GAL6 | 6 | 8.55E-04 | 1152862 | 0.256581 |
| Down | RUNDC3B | 6 | 4.17E-04 | 520596 | 0.25848 |
| Down | FBLN7 | 6 | 3.05E-04 | 519984 | 0.261195 |
| Down | SULF1 | 6 | 1.72E-04 | 242586 | 0.2663 |
| Down | LIX1L | 6 | 3.11E-04 | 615256 | 0.253358 |
| Down | CIART | 6 | 5.63E-04 | 638684 | 0.259311 |
| Down | PLIN5 | 6 | 8.30E-04 | 1453450 | 0.21646 |
| Down | RAMP2 | 6 | 4.80E-04 | 195708 | 0.197799 |
| Down | ACSS2 | 5 | 2.81E-04 | 453366 | 0.241728 |
| Down | RNF217 | 5 | 2.85E-04 | 487428 | 0.239844 |
| Down | SRPX | 5 | 2.61E-05 | 35474 | 0.269192 |
| Down | ACADL | 5 | 3.59E-04 | 454718 | 0.258812 |
| Down | SFTPD | 5 | 0.001101 | 1022596 | 0.195741 |
| Down | CYP4V2 | 5 | 5.82E-04 | 277440 | 0.261515 |
| Down | RWDD2A | 5 | 4.43E-05 | 98420 | 0.251193 |
| Down | TTC38 | 4 | 5.49E-06 | 6228 | 0.22522 |
| Down | GAS1 | 4 | 1.09E-04 | 69102 | 0.272802 |
| Down | ZNF280D | 4 | 6.05E-06 | 14918 | 0.242414 |
| Down | AACS | 4 | 3.19E-04 | 524692 | 0.216847 |
| Down | KLF9 | 4 | 3.18E-05 | 25494 | 0.279544 |
| Down | NXNL1 | 4 | 3.36E-06 | 9678 | 0.242252 |
| Down | HLF | 4 | 5.51E-04 | 279718 | 0.212831 |
| Down | CA3 | 4 | 6.16E-05 | 104110 | 0.244413 |
| Down | PHF13 | 4 | 1 | 12 | 1 |
| Down | SOX17 | 4 | 9.55E-06 | 16194 | 0.23657 |
| Down | LARP6 | 3 | 1.86E-05 | 10288 | 0.256137 |
| Down | FHOD3 | 3 | 2.12E-06 | 5676 | 0.252284 |
| Down | HLA-DMA | 3 | 2.75E-04 | 1237430 | 0.181505 |
| Down | DMRT2 | 3 | 6.54E-06 | 12390 | 0.230291 |
| Down | LEFTY2 | 3 | 5.51E-04 | 526654 | 0.2086 |
| Down | ELOVL6 | 3 | 2.78E-04 | 223110 | 0.201097 |
| Down | PKD1L2 | 2 | 1.32E-06 | 3454 | 0.235963 |
| Down | GATM | 2 | 2.75E-04 | 550962 | 0.189267 |
| Down | BEX4 | 2 | 2.75E-04 | 248730 | 0.202544 |
| Down | ZNF334 | 2 | 3.94E-06 | 6896 | 0.23337 |
| Down | ANKRD37 | 2 | 7.60E-07 | 164 | 0.206471 |
| Down | CMTM8 | 2 | 1.07E-05 | 39428 | 0.259719 |
| Down | ADH1C | 2 | 2.75E-04 | 372628 | 0.207125 |
| Down | SLC4A4 | 2 | 1.07E-06 | 826 | 0.210456 |
| Down | MT1G | 2 | 2.32E-06 | 1756 | 0.199533 |
| Down | ZFAND1 | 1 | 0 | 0 | 0.259942 |
| Down | DNAJC27 | 1 | 0 | 0 | 0.217653 |
| Down | WNT3 | 1 | 0 | 0 | 0.204258 |
| Down | RNASE4 | 1 | 0 | 0 | 0.222959 |
| Down | DNAH17 | 1 | 0 | 0 | 0.21384 |
| Down | C1QTNF7 | 1 | 0 | 0 | 0.218748 |

**Table 8** Target gene - miRNA interaction table

| **Regulation** | **Target gene** | **Degree** | **MicroRNA** | **Regulation** | **Target gene** | **Degree** | **MicroRNA** |
| --- | --- | --- | --- | --- | --- | --- | --- |
| Up | SOD2 | 257 | hsa-mir-3144-3p | Down | BTG2 | 247 | hsa-mir-6075 |
| Up | CCND1 | 251 | hsa-mir-7706 | Down | TXNIP | 228 | hsa-mir-3194-3p |
| Up | TUBB2A | 193 | hsa-mir-7162-3p | Down | MED28 | 203 | hsa-mir-6861-5p |
| Up | CCND2 | 179 | hsa-mir-5692c | Down | CNBP | 197 | hsa-mir-4651 |
| Up | TMEM189 | 146 | hsa-mir-548z | Down | MKNK2 | 195 | hsa-mir-3650 |
| Up | SAR1A | 145 | hsa-mir-3929 | Down | DCAF7 | 174 | hsa-mir-550b-2-5p |
| Up | ZMAT3 | 145 | hsa-mir-5009-3p | Down | CDKN1B | 146 | hsa-mir-2355-5p |
| Up | IGFBP5 | 144 | hsa-mir-548az-3p | Down | FAM126B | 141 | hsa-mir-7703 |
| Up | CYCS | 137 | hsa-mir-6836-5p | Down | GLUL | 126 | hsa-mir-7160-5p |
| Up | CCDC80 | 135 | hsa-mir-6126 | Down | HSD17B12 | 126 | hsa-mir-6840-3p |
| Up | TGFBR2 | 110 | hsa-mir-6758-5p | Down | PTEN | 125 | hsa-mir-217 |
| Up | MSN | 108 | hsa-mir-4739 | Down | ZNF394 | 122 | hsa-mir-4649-3p |
| Up | NAV1 | 108 | hsa-mir-7150 | Down | KCNK6 | 117 | hsa-mir-4731-5p |
| Up | CPE | 98 | hsa-mir-7160-5p | Down | SRSF7 | 108 | hsa-mir-30c-2-3p |
| Up | THBS1 | 95 | hsa-mir-6825-5p | Down | MED16 | 104 | hsa-mir-30c-1-3p |
| Up | HOXC8 | 95 | hsa-mir-7111-5p | Down | CCNF | 102 | hsa-mir-3155b |
| Up | FAM129B | 92 | hsa-mir-6855-3p | Down | FOXN2 | 101 | hsa-mir-3133 |
| Up | OSTM1 | 90 | hsa-mir-4794 | Down | NR3C1 | 100 | hsa-mir-3609 |
| Up | STC2 | 90 | hsa-mir-206 | Down | IGF1 | 98 | hsa-mir-299-3p |
| Up | HSPA8 | 86 | hsa-mir-5697 | Down | SHMT1 | 97 | hsa-mir-4477a |
| Up | SUSD1 | 86 | hsa-mir-4728-5p | Down | RPS6KA5 | 95 | hsa-mir-4423-5p |
| Up | DSEL | 85 | hsa-mir-7977 | Down | CBS | 94 | hsa-mir-6883-5p |
| Up | STAT3 | 80 | hsa-mir-6833-3p | Down | RNF125 | 92 | hsa-mir-4257 |
| Up | VLDLR | 77 | hsa-mir-548ad-5p | Down | LDHD | 91 | hsa-mir-6512-5p |
| Up | SERPINH1 | 77 | hsa-mir-6808-5p | Down | NDUFV3 | 91 | hsa-mir-548an |
| Up | BAMBI | 76 | hsa-mir-3649 | Down | RPL14 | 90 | hsa-mir-3960 |
| Up | KCNB1 | 74 | hsa-mir-5695 | Down | HMGB2 | 89 | hsa-mir-6894-3p |
| Up | ERBB2 | 73 | hsa-mir-3921 | Down | FKBP5 | 88 | hsa-mir-6852-5p |
| Up | SLC43A2 | 70 | hsa-mir-6852-5p | Down | ACACA | 86 | hsa-mir-378d |
| Up | MAP1B | 68 | hsa-mir-5697 | Down | RPL18A | 85 | hsa-mir-6784-5p |
| Up | CALU | 67 | hsa-mir-1537-5p | Down | RBBP4 | 84 | hsa-mir-5696 |
| Up | BHMT2 | 67 | hsa-mir-520h | Down | HOXA10 | 83 | hsa-mir-7113-3p |
| Up | CRTAP | 67 | hsa-mir-1273h-5p | Down | PER2 | 82 | hsa-mir-4433a-3p |
| Up | RHOC | 65 | hsa-mir-591 | Down | ELP2 | 80 | hsa-mir-7111-3p |
| Up | GNS | 64 | hsa-mir-6808-5p | Down | EIF4EBP2 | 78 | hsa-mir-944 |
| Up | WWC1 | 63 | hsa-mir-5697 | Down | RPS15A | 73 | hsa-mir-6720-5p |
| Up | MXRA7 | 61 | hsa-mir-203a-3p | Down | ESR1 | 72 | hsa-mir-3668 |
| Up | PMEPA1 | 59 | hsa-mir-6879-5p | Down | CD55 | 71 | hsa-mir-6813-3p |
| Up | AEN | 59 | hsa-mir-4725-3p | Down | FOXO1 | 68 | hsa-mir-4753-3p |
| Up | PLXNA1 | 58 | hsa-mir-940 | Down | FADS1 | 68 | hsa-mir-3189-5p |
| Up | GRIN2B | 58 | hsa-mir-4469 | Down | RGMB | 67 | hsa-mir-5681a |
| Up | C1RL | 57 | hsa-mir-3663-3p | Down | RTN2 | 66 | hsa-mir-4680-5p |
| Up | SGK1 | 57 | hsa-mir-3646 | Down | LIX1L | 65 | hsa-mir-4421 |
| Up | TAGLN2 | 57 | hsa-mir-1972 | Down | TGIF1 | 65 | hsa-mir-7-2-3p |
| Up | YWHAH | 55 | hsa-mir-6865-3p | Down | FOXO3 | 64 | hsa-mir-5590-3p |
| Up | SLC35G1 | 55 | hsa-mir-6854-5p | Down | EEF2K | 64 | hsa-mir-4728-5p |
| Up | GPR137B | 54 | hsa-mir-520c-3p | Down | ZNF581 | 63 | hsa-mir-6877-5p |
| Up | B4GALT5 | 54 | hsa-mir-4306 | Down | EFNA1 | 62 | hsa-mir-6893-5p |
| Up | PTGFRN | 53 | hsa-mir-6828-3p | Down | RSBN1 | 57 | hsa-mir-548as-5p |
| Up | SLC2A5 | 51 | hsa-mir-6893-5p | Down | PHF13 | 55 | hsa-mir-7977 |
| Up | PPP2R1B | 48 | hsa-mir-8082 | Down | GMPS | 54 | hsa-mir-4267 |
| Up | SCIN | 48 | hsa-mir-3163 | Down | ALDH6A1 | 54 | hsa-mir-6847-3p |
| Up | ZCCHC24 | 48 | hsa-mir-4290 | Down | ELK1 | 54 | hsa-mir-4704-3p |
| Up | HMOX1 | 47 | hsa-mir-4443 | Down | H1F0 | 52 | hsa-mir-548ah-5p |
| Up | PLIN3 | 45 | hsa-mir-4701-3p | Down | DNAH17 | 51 | hsa-mir-4658 |
| Up | AIFM2 | 44 | hsa-mir-6826-5p | Down | BTG3 | 50 | hsa-mir-4509 |
| Up | NABP1 | 43 | hsa-mir-3666 | Down | GATM | 49 | hsa-mir-548bb-3p |
| Up | STEAP2 | 43 | hsa-mir-6501-3p | Down | TWIST1 | 49 | hsa-mir-4672 |
| Up | TRAM2 | 43 | hsa-mir-3065-3p | Down | DMRT2 | 48 | hsa-mir-1253 |
| Up | HSPA5 | 42 | hsa-mir-4650-3p | Down | PPP3R1 | 48 | hsa-mir-548as-3p |
| Up | MARCKS | 41 | hsa-mir-4687-3p | Down | MLX | 48 | hsa-mir-6747-5p |
| Up | ADAMTS5 | 40 | hsa-mir-548am-3p | Down | RNF38 | 47 | hsa-mir-3166 |
| Up | SRPX2 | 40 | hsa-mir-3178 | Down | RFTN2 | 46 | hsa-mir-3689b-3p |
| Up | HLA-A | 39 | hsa-mir-2110 | Down | TMEM100 | 45 | hsa-mir-302c-3p |
| Up | FSTL3 | 39 | hsa-mir-6085 | Down | ANG | 45 | hsa-mir-6839-5p |
| Up | LYZ | 38 | hsa-mir-661 | Down | LETMD1 | 45 | hsa-mir-6885-3p |
| Up | QPRT | 38 | hsa-mir-4755-5p | Down | SPSB1 | 45 | hsa-mir-3924 |
| Up | MFRP | 36 | hsa-mir-6842-5p | Down | SC5D | 44 | hsa-mir-4793-3p |
| Up | KCTD12 | 35 | hsa-mir-6754-3p | Down | SIN3A | 44 | hsa-mir-1343-5p |
| Up | SKAP2 | 35 | hsa-mir-4778-5p | Down | MTUS1 | 43 | hsa-mir-4266 |
| Up | CDK14 | 35 | hsa-mir-4533 | Down | ZBTB16 | 43 | hsa-mir-6749-3p |
| Up | KIAA1671 | 34 | hsa-mir-6895-3p | Down | N4BP2L2 | 43 | hsa-mir-3149 |
| Up | CYB561 | 34 | hsa-mir-1827 | Down | PFKFB3 | 43 | hsa-mir-4644 |
| Up | CYR61 | 33 | hsa-mir-6516-5p | Down | PDLIM3 | 43 | hsa-mir-4755-3p |
| Up | EMX2 | 33 | hsa-mir-6867-3p | Down | TMLHE | 43 | hsa-mir-548ac |
| Up | ALDH1A3 | 32 | hsa-mir-1185-2-3p | Down | NRIP1 | 42 | hsa-mir-6511a-3p |
| Up | THY1 | 32 | hsa-mir-4685-3p | Down | ACADL | 42 | hsa-mir-3689c |
| Up | CXCL10 | 31 | hsa-mir-767-5p | Down | TRIM33 | 41 | hsa-mir-4768-5p |
| Up | CCPG1 | 31 | hsa-mir-558 | Down | ELOVL6 | 41 | hsa-mir-4252 |
| Up | TAGLN | 31 | hsa-mir-6785-5p | Down | CABLES1 | 40 | hsa-mir-548t-3p |
| Up | SOBP | 31 | hsa-mir-487b-3p | Down | ORMDL3 | 40 | hsa-mir-5695 |
| Up | TPM2 | 30 | hsa-mir-4801 | Down | PHIP | 38 | hsa-mir-4729 |
| Up | CTGF | 30 | hsa-mir-133b | Down | RNF217 | 38 | hsa-mir-5190 |
| Up | COL1A2 | 30 | hsa-mir-5010-5p | Down | ARRB1 | 38 | hsa-mir-3934-5p |
| Up | RAB31 | 30 | hsa-mir-3117-5p | Down | SIRT1 | 37 | hsa-mir-4474-5p |
| Up | HOXB8 | 30 | hsa-mir-129-1-3p | Down | NOP53 | 36 | hsa-mir-3617-5p |
| Up | RAB9A | 30 | hsa-mir-6511b-5p | Down | TSC22D3 | 35 | hsa-mir-4639-3p |
| Up | HAVCR2 | 30 | hsa-mir-1273h-5p | Down | GAS1 | 35 | hsa-mir-5692a |
| Up | TPST2 | 29 | hsa-mir-7151-3p | Down | CLDND1 | 35 | hsa-mir-1178-5p |
| Up | COL12A1 | 28 | hsa-mir-6079 | Down | AIP | 35 | hsa-mir-6870-5p |
| Up | MSC | 28 | hsa-mir-4745-5p | Down | BHLHE40 | 34 | hsa-mir-7113-5p |
| Up | CEP19 | 27 | hsa-mir-5688 | Down | EIF2A | 34 | hsa-mir-6790-3p |
| Up | KLF4 | 26 | hsa-mir-130a-3p | Down | AZIN1 | 34 | hsa-mir-6780b-5p |
| Up | FRMD6 | 26 | hsa-mir-2276-5p | Down | ERRFI1 | 34 | hsa-mir-3922-5p |
| Up | SOS1 | 26 | hsa-mir-5571-5p | Down | ZNF395 | 34 | hsa-mir-548o-5p |
| Up | CORO1C | 26 | hsa-mir-6751-3p | Down | THYN1 | 33 | hsa-mir-4500 |
| Up | TMEM54 | 26 | hsa-mir-4472 | Down | CCDC59 | 33 | hsa-mir-1972 |
| Up | MFAP5 | 26 | hsa-mir-5692b | Down | P3H2 | 33 | hsa-mir-6851-5p |
| Up | DHDDS | 26 | hsa-mir-4710 | Down | ACVR1 | 32 | hsa-mir-3666 |
| Up | CYB561A3 | 25 | hsa-mir-4433b-3p | Down | ZFAND5 | 31 | hsa-mir-4459 |
| Up | PALLD | 24 | hsa-mir-1298-5p | Down | MKX | 31 | hsa-mir-548g-5p |
| Up | LOX | 24 | hsa-mir-3591-5p | Down | EIF3H | 31 | hsa-mir-3187-5p |
| Up | SAA1 | 24 | hsa-mir-4441 | Down | RUNDC3B | 31 | hsa-mir-5088-3p |
| Up | S100A11 | 24 | hsa-mir-1207-5p | Down | CDS2 | 30 | hsa-mir-6852-3p |
| Up | AMOT | 23 | hsa-mir-6806-3p | Down | LAPTM4B | 30 | hsa-mir-548x-5p |
| Up | FTH1 | 23 | hsa-mir-6126 | Down | WNT3 | 30 | hsa-mir-6130 |
| Up | VASN | 23 | hsa-mir-449a | Down | PRUNE2 | 29 | hsa-mir-518a-5p |
| Up | FBLN2 | 23 | hsa-mir-548am-3p | Down | CCNB1IP1 | 29 | hsa-mir-6770-5p |
| Up | KLHL30 | 23 | hsa-mir-4695-3p | Down | TTC38 | 29 | hsa-mir-4770 |
| Up | NCKAP5L | 22 | hsa-mir-4640-5p | Down | PHF3 | 28 | hsa-mir-4530 |
| Up | TIMP1 | 22 | hsa-mir-8059 | Down | USP13 | 28 | hsa-mir-548aw |
| Up | ELN | 22 | hsa-mir-2115-3p | Down | RFX1 | 28 | hsa-mir-6801-5p |
| Up | CDR2L | 22 | hsa-mir-1295b-5p | Down | FAM107A | 28 | hsa-mir-6749-3p |
| Up | MMP9 | 21 | hsa-mir-211-5p | Down | RPS9 | 28 | hsa-mir-7111-3p |
| Up | SNCG | 21 | hsa-mir-6838-5p | Down | GINS3 | 28 | hsa-mir-3155a |
| Up | TRIM6 | 21 | hsa-mir-5186 | Down | RAD21 | 27 | hsa-mir-4428 |
| Up | SPARC | 21 | hsa-mir-7855-5p | Down | RPS3 | 27 | hsa-mir-6844 |
| Up | SH3BGRL3 | 21 | hsa-mir-4794 | Down | MSRB1 | 27 | hsa-mir-216b-5p |
| Up | RAB6B | 21 | hsa-mir-6797-5p | Down | TRIM24 | 26 | hsa-mir-548ap-3p |
| Up | CYP11A1 | 21 | hsa-mir-6766-5p | Down | PHLPP1 | 26 | hsa-mir-1307-3p |
| Up | INF2 | 20 | hsa-mir-4646-3p | Down | NASP | 26 | hsa-mir-3127-3p |
| Up | CXCL12 | 20 | hsa-mir-7152-3p | Down | NECTIN3 | 26 | hsa-mir-548b-5p |
| Up | LHX6 | 20 | hsa-mir-5690 | Down | RPL9 | 25 | hsa-mir-4729 |
| Up | TMEM98 | 20 | hsa-mir-5590-3p | Down | RPS2 | 25 | hsa-mir-196a-5p |
| Up | HSPB8 | 20 | hsa-mir-4657 | Down | MAPK10 | 25 | hsa-mir-6737-5p |
| Up | CLIC6 | 20 | hsa-mir-3689e | Down | RWDD2A | 25 | hsa-mir-548at-3p |
| Up | EPDR1 | 19 | hsa-mir-374a-5p | Down | RPS27 | 24 | hsa-mir-1260a |
| Up | STAT1 | 19 | hsa-mir-1183 | Down | PER3 | 24 | hsa-mir-4700-5p |
| Up | IRAK1 | 18 | hsa-mir-1226-3p | Down | SLC25A23 | 24 | hsa-mir-4441 |
| Up | FAM98A | 18 | hsa-mir-3158-5p | Down | STARD13 | 24 | hsa-mir-219a-1-3p |
| Up | VGLL3 | 18 | hsa-mir-4252 | Down | ADH1B | 24 | hsa-mir-300 |
| Up | PRSS23 | 18 | hsa-mir-4672 | Down | KLF9 | 23 | hsa-mir-6750-3p |
| Up | PLA2G7 | 18 | hsa-mir-4524b-3p | Down | NDFIP2 | 23 | hsa-mir-664b-3p |
| Up | CD68 | 18 | hsa-mir-7113-3p | Down | FAM13A | 23 | hsa-mir-3679-3p |
| Up | FAR2 | 18 | hsa-mir-3924 | Down | MAPRE2 | 23 | hsa-mir-3140-3p |
| Up | MN1 | 17 | hsa-mir-3667-3p | Down | SRSF3 | 23 | hsa-mir-6860 |
| Up | PLEKHO2 | 17 | hsa-mir-6515-3p | Down | UBE2W | 22 | hsa-mir-5197-3p |
| Up | ITIH5 | 17 | hsa-mir-4753-3p | Down | SOX17 | 22 | hsa-mir-3118 |
| Up | KANK4 | 17 | hsa-mir-1290 | Down | GJA1 | 21 | hsa-mir-1298-5p |
| Up | TNS3 | 16 | hsa-mir-6071 | Down | HLF | 21 | hsa-mir-664a-3p |
| Up | DBNDD1 | 16 | hsa-mir-4300 | Down | DPF2 | 20 | hsa-mir-5088-3p |
| Up | SLC46A3 | 16 | hsa-mir-6796-3p | Down | FGFRL1 | 20 | hsa-mir-6790-5p |
| Up | CHST3 | 16 | hsa-mir-6131 | Down | POLR1D | 20 | hsa-mir-1273e |
| Up | LRRC32 | 16 | hsa-mir-6779-3p | Down | MAP3K5 | 20 | hsa-mir-4775 |
| Up | SLC22A12 | 16 | hsa-mir-3189-5p | Down | CYP4V2 | 20 | hsa-mir-4324 |
| Up | PCDH9 | 14 | hsa-mir-4635 | Down | SNTB1 | 20 | hsa-mir-199b-5p |
| Up | SMYD2 | 14 | hsa-mir-4516 | Down | TTLL7 | 20 | hsa-mir-4677-3p |
| Up | SCARB1 | 14 | hsa-mir-5091 | Down | CD36 | 20 | hsa-mir-6516-3p |
| Up | MYL9 | 14 | hsa-mir-6764-5p | Down | RPL35 | 20 | hsa-mir-7157-3p |
| Up | CST3 | 13 | hsa-mir-4768-5p | Down | DNAJC27 | 19 | hsa-mir-27a-3p |
| Up | NRP2 | 13 | hsa-mir-374c-5p | Down | SERTAD4 | 19 | hsa-mir-6883-3p |
| Up | CD9 | 13 | hsa-mir-4727-3p | Down | POLR1E | 19 | hsa-mir-6893-5p |
| Up | TPGS2 | 12 | hsa-mir-1252-3p | Down | SLC4A4 | 19 | hsa-mir-939-3p |
| Up | DLGAP4 | 12 | hsa-mir-4768-3p | Down | PCYT2 | 18 | hsa-mir-3960 |
| Up | CRABP2 | 12 | hsa-mir-449b-5p | Down | ADD3 | 18 | hsa-mir-4776-3p |
| Up | SPATA18 | 12 | hsa-mir-6840-3p | Down | CTH | 18 | hsa-mir-548s |
| Up | MFGE8 | 12 | hsa-mir-4442 | Down | MARCH3 | 18 | hsa-mir-4715-3p |
| Up | COL3A1 | 11 | hsa-mir-29c-3p | Down | RSL24D1 | 18 | hsa-mir-4274 |
| Up | MT2A | 11 | hsa-mir-6823-5p | Down | GTF3C3 | 17 | hsa-mir-6760-5p |
| Up | ANKDD1A | 11 | hsa-mir-4695-5p | Down | BDH1 | 17 | hsa-mir-298 |
| Up | PPDPF | 11 | hsa-mir-5096 | Down | PLIN5 | 17 | hsa-mir-6890-3p |
| Up | LAMA5 | 11 | hsa-mir-1909-3p | Down | PHGDH | 17 | hsa-mir-4257 |
| Up | SLC24A3 | 11 | hsa-mir-7845-5p | Down | MED30 | 17 | hsa-mir-6130 |
| Up | EGR2 | 10 | hsa-mir-20a-5p | Down | ZFAND1 | 17 | hsa-mir-3622b-5p |
| Up | PTGER4 | 10 | hsa-mir-20b-5p | Down | SRSF4 | 17 | hsa-mir-6761-5p |
| Up | ENC1 | 10 | hsa-mir-6733-5p | Down | HDGFL2 | 17 | hsa-mir-2682-5p |
| Up | C1S | 10 | hsa-mir-548s | Down | RPL18 | 16 | hsa-mir-4661-3p |
| Up | SVEP1 | 10 | hsa-mir-6888-5p | Down | RPS17 | 16 | hsa-mir-4433b-5p |
| Up | TNFRSF12A | 9 | hsa-mir-92a-1-5p | Down | NR4A2 | 16 | hsa-mir-7157-5p |
| Up | DAG1 | 9 | hsa-mir-3676-3p | Down | STAG2 | 16 | hsa-mir-3651 |
| Up | GNG2 | 9 | hsa-mir-4713-5p | Down | RORB | 16 | hsa-mir-4282 |
| Up | SEMA3G | 9 | hsa-mir-4287 | Down | DAPK2 | 16 | hsa-mir-7161-5p |
| Up | NOTCH3 | 9 | hsa-mir-206 | Down | GPR146 | 16 | hsa-mir-660-3p |
| Up | OAF | 9 | hsa-mir-6868-3p | Down | SYT17 | 16 | hsa-mir-4311 |
| Up | IRX2 | 9 | hsa-mir-1305 | Down | SIVA1 | 15 | hsa-mir-581 |
| Up | FCMR | 9 | hsa-mir-1260a | Down | TSC22D1 | 15 | hsa-mir-590-3p |
| Up | CTHRC1 | 8 | hsa-mir-548l | Down | ACACB | 15 | hsa-mir-6797-3p |
| Up | RND3 | 8 | hsa-mir-200c-3p | Down | NDRG4 | 15 | hsa-mir-1299 |
| Up | NUP188 | 8 | hsa-mir-7847-3p | Down | PLSCR4 | 14 | hsa-mir-1178-3p |
| Up | CLPTM1L | 8 | hsa-mir-4694-3p | Down | SCP2 | 14 | hsa-mir-4776-3p |
| Up | FAM198B | 8 | hsa-mir-1273e | Down | GID8 | 14 | hsa-mir-340-5p |
| Up | TMED3 | 8 | hsa-mir-1260b | Down | SLC3A2 | 13 | hsa-mir-8068 |
| Up | ITGAV | 8 | hsa-mir-548c-3p | Down | FBXO11 | 13 | hsa-mir-4453 |
| Up | ALCAM | 8 | hsa-mir-148a-3p | Down | OSBPL1A | 13 | hsa-mir-5003-3p |
| Up | GSTM5 | 8 | hsa-mir-6793-3p | Down | MIS18BP1 | 13 | hsa-mir-3679-5p |
| Up | C1R | 8 | hsa-mir-6824-3p | Down | FMC1 | 13 | hsa-mir-5095 |
| Up | COL5A2 | 7 | hsa-mir-767-5p | Down | RERE | 13 | hsa-mir-4763-5p |
| Up | CD248 | 7 | hsa-mir-4436b-5p | Down | ARHGEF3 | 13 | hsa-mir-4793-5p |
| Up | LCP1 | 7 | hsa-mir-520a-3p | Down | PMP22 | 12 | hsa-mir-4769-5p |
| Up | S100A9 | 7 | hsa-mir-4252 | Down | ADNP2 | 12 | hsa-mir-5004-5p |
| Up | ZNF219 | 7 | hsa-mir-7845-5p | Down | STK24 | 12 | hsa-mir-766-3p |
| Up | TUBB8 | 7 | hsa-mir-5680 | Down | BEX4 | 12 | hsa-mir-5580-3p |
| Up | COL6A1 | 6 | hsa-mir-1301-3p | Down | CKB | 11 | hsa-mir-551a |
| Up | ACTN1 | 6 | hsa-let-7e-5p | Down | SLC27A2 | 11 | hsa-mir-7113-3p |
| Up | HTRA1 | 6 | hsa-mir-6774-5p | Down | RPL21 | 11 | hsa-mir-218-5p |
| Up | TUBB2B | 6 | hsa-mir-186-5p | Down | NBEA | 11 | hsa-mir-9-5p |
| Up | SPP1 | 6 | hsa-mir-146a-5p | Down | CD302 | 11 | hsa-mir-4690-3p |
| Up | SYNC | 6 | hsa-mir-3122 | Down | ORC2 | 11 | hsa-mir-6728-3p |
| Up | CADM3 | 6 | hsa-mir-5689 | Down | GMCL1 | 11 | hsa-mir-6864-3p |
| Up | NEK6 | 5 | hsa-mir-1307-3p | Down | ELF2 | 11 | hsa-mir-190a-3p |
| Up | UCHL1 | 5 | hsa-mir-181a-5p | Down | AACS | 10 | hsa-mir-1470 |
| Up | TDP1 | 5 | hsa-mir-320a | Down | LEFTY2 | 10 | hsa-mir-660-3p |
| Up | LEP | 5 | hsa-mir-7856-5p | Down | ATP8B4 | 10 | hsa-mir-208b-5p |
| Up | COL6A2 | 5 | hsa-mir-10a-5p | Down | TRIP4 | 10 | hsa-mir-4728-3p |
| Up | GPC1 | 5 | hsa-mir-140-3p | Down | RBL2 | 9 | hsa-mir-106a-5p |
| Up | IFIT2 | 5 | hsa-mir-645 | Down | REV1 | 9 | hsa-mir-20b-5p |
| Up | PLBD2 | 5 | hsa-mir-3607-3p | Down | PCCA | 9 | hsa-mir-1273e |
| Up | NPR3 | 4 | hsa-mir-16-5p | Down | RFX2 | 9 | hsa-mir-3131 |
| Up | TNFRSF11B | 4 | hsa-mir-181a-5p | Down | PCBD1 | 9 | hsa-mir-5584-5p |
| Up | FGF11 | 4 | hsa-mir-193b-3p | Down | TBX15 | 9 | hsa-mir-5582-5p |
| Up | CMTM3 | 4 | hsa-mir-24-3p | Down | ZC2HC1A | 9 | hsa-mir-1914-3p |
| Up | ANO3 | 4 | hsa-mir-186-5p | Down | EIF4B | 8 | hsa-mir-423-3p |
| Up | COL15A1 | 4 | hsa-mir-29b-3p | Down | RPS12 | 8 | hsa-mir-181b-5p |
| Up | SFRP4 | 4 | hsa-mir-103a-3p | Down | STK26 | 8 | hsa-mir-5000-5p |
| Up | PDLIM1 | 4 | hsa-mir-504-5p | Down | INTS6 | 8 | hsa-mir-4317 |
| Up | PYGL | 4 | hsa-mir-155-5p | Down | KHDRBS1 | 8 | hsa-mir-27b-3p |
| Up | FAM171A1 | 4 | hsa-mir-124-3p | Down | PELI2 | 8 | hsa-mir-151a-5p |
| Up | ABCC3 | 4 | hsa-mir-197-3p | Down | RPL29 | 8 | hsa-mir-92a-1-5p |
| Up | SPTAN1 | 4 | hsa-mir-128-3p | Down | MAT2B | 7 | hsa-mir-377-3p |
| Up | FAT1 | 4 | hsa-mir-222-3p | Down | ST3GAL6 | 7 | hsa-mir-6868-3p |
| Up | NMT2 | 4 | hsa-mir-181d-5p | Down | FBXO46 | 7 | hsa-mir-513a-5p |
| Up | STK38L | 4 | hsa-mir-130a-3p | Down | SAFB | 6 | hsa-mir-1229-3p |
| Up | FRZB | 4 | hsa-mir-30b-5p | Down | PDK4 | 6 | hsa-mir-182-5p |
| Up | TM4SF19 | 4 | hsa-mir-550a-3p | Down | RPL17 | 6 | hsa-mir-519d-3p |
| Up | TNFAIP6 | 4 | hsa-mir-19b-2-5p | Down | LEMD3 | 6 | hsa-mir-10b-5p |
| Up | C3orf14 | 4 | hsa-mir-6072 | Down | CAB39L | 6 | hsa-mir-10a-5p |
| Up | ITGB5 | 3 | hsa-let-7b-5p | Down | MCM6 | 6 | hsa-mir-1180-3p |
| Up | LAMB3 | 3 | hsa-mir-218-5p | Down | RHOT1 | 5 | hsa-mir-15a-5p |
| Up | PLPP1 | 3 | hsa-mir-124-3p | Down | HDHD5 | 5 | hsa-mir-484 |
| Up | LBP | 3 | hsa-mir-22-3p | Down | NET1 | 5 | hsa-mir-320a |
| Up | GLS | 3 | hsa-mir-7-5p | Down | KAT14 | 5 | hsa-mir-5196-3p |
| Up | ITGB2 | 3 | hsa-mir-146a-5p | Down | RPS18 | 5 | hsa-mir-421 |
| Up | CXCL9 | 3 | hsa-mir-34a-5p | Down | LSS | 5 | hsa-mir-30a-5p |
| Up | RAB7B | 3 | hsa-mir-92b-3p | Down | BCAT2 | 5 | hsa-mir-615-3p |
| Up | CFB | 3 | hsa-mir-210-5p | Down | FAM214A | 5 | hsa-mir-222-3p |
| Up | MSTO1 | 3 | hsa-mir-10b-5p | Down | CCNG2 | 5 | hsa-mir-93-3p |
| Up | DOCK6 | 3 | hsa-mir-361-5p | Down | CTSC | 5 | hsa-mir-199a-5p |
| Up | SYNPO2 | 3 | hsa-mir-144-5p | Down | SOCS2 | 4 | hsa-mir-16-5p |
| Up | CCDC107 | 3 | hsa-mir-5193 | Down | GPD1L | 4 | hsa-mir-210-3p |
| Up | GLB1 | 2 | hsa-mir-155-5p | Down | RNF144A | 4 | hsa-mir-24-3p |
| Up | APLNR | 2 | hsa-mir-16-5p | Down | RGS3 | 4 | hsa-mir-133a-3p |
| Up | FMOD | 2 | hsa-mir-21-5p | Down | CISH | 4 | hsa-mir-150-5p |
| Up | FILIP1L | 2 | hsa-mir-335-5p | Down | S100A1 | 4 | hsa-mir-138-5p |
| Up | PDGFD | 2 | hsa-mir-145-5p | Down | RPS29 | 4 | hsa-mir-652-3p |
| Up | HSD11B1 | 2 | hsa-mir-26b-5p | Down | EEF1D | 4 | hsa-mir-149-5p |
| Up | GPNMB | 2 | hsa-mir-508-5p | Down | CNRIP1 | 4 | hsa-mir-3977 |
| Up | PCBP3 | 2 | hsa-mir-26b-5p | Down | ANK2 | 4 | hsa-mir-122-5p |
| Up | HSPA12A | 2 | hsa-mir-339-5p | Down | ZNF280D | 4 | hsa-mir-505-5p |
| Up | MGST1 | 2 | hsa-mir-652-3p | Down | TMOD1 | 4 | hsa-mir-1185-1-3p |
| Up | AKR1C3 | 2 | hsa-mir-98-5p | Down | WNT11 | 4 | hsa-mir-5580-3p |
| Up | FAM189A2 | 2 | hsa-mir-192-5p | Down | LACTB2 | 3 | hsa-mir-30c-5p |
| Up | INHBB | 2 | hsa-mir-34a-5p | Down | FBL | 3 | hsa-mir-20a-5p |
| Up | SLIT2 | 2 | hsa-mir-330-3p | Down | PLD1 | 3 | hsa-mir-638 |
| Up | FLNC | 2 | hsa-mir-1-3p | Down | AUTS2 | 3 | hsa-mir-21-5p |
| Up | SHTN1 | 2 | hsa-mir-106b-5p | Down | PCM1 | 3 | hsa-mir-8055 |
| Up | MFAP4 | 2 | hsa-mir-449a | Down | H1FX | 3 | hsa-mir-1-3p |
| Up | WISP2 | 2 | hsa-mir-124-3p | Down | MED6 | 3 | hsa-mir-199a-3p |
| Up | GLIPR2 | 2 | hsa-mir-155-5p | Down | RNASE4 | 3 | hsa-mir-215-5p |
| Up | CYTOR | 2 | hsa-mir-376c-3p | Down | RBMX | 3 | hsa-mir-196a-5p |
| Up | GALNT16 | 2 | hsa-mir-140-5p | Down | GPC3 | 3 | hsa-mir-1271-5p |
| Up | LHCGR | 2 | hsa-mir-513a-3p | Down | HDAC9 | 3 | hsa-mir-29a-5p |
| Up | LAMB2 | 2 | hsa-mir-196b-5p | Down | PAPSS1 | 2 | hsa-mir-100-5p |
| Up | IRF8 | 2 | hsa-mir-646 | Down | GPHN | 2 | hsa-let-7b-5p |
| Up | NRCAM | 2 | hsa-mir-4282 | Down | AHCTF1 | 2 | hsa-mir-186-5p |
| Up | SULT1A1 | 1 | hsa-mir-631 | Down | CENPV | 2 | hsa-mir-28-5p |
| Up | CLCA2 | 1 | hsa-let-7b-5p | Down | RAI2 | 2 | hsa-let-7e-5p |
| Up | AOX1 | 1 | hsa-mir-26b-5p | Down | EEF1G | 2 | hsa-mir-218-5p |
| Up | GRB14 | 1 | hsa-mir-26b-5p | Down | ECI1 | 2 | hsa-mir-155-5p |
| Up | TYMP | 1 | hsa-mir-92a-3p | Down | BLVRB | 2 | hsa-mir-127-5p |
| Up | HBB | 1 | hsa-mir-92a-3p | Down | MPDZ | 2 | hsa-mir-26b-5p |
| Up | CES1 | 1 | hsa-mir-197-3p | Down | HSD17B11 | 2 | hsa-mir-1-3p |
| Up | MYEOV | 1 | hsa-mir-7-5p | Down | EYA1 | 2 | hsa-mir-101-3p |
| Up | COL16A1 | 1 | hsa-mir-181a-5p | Down | LRRCC1 | 2 | hsa-mir-215-5p |
| Up | LRRN3 | 1 | hsa-mir-181a-5p | Down | C5 | 2 | hsa-mir-10a-5p |
| Up | S100A4 | 1 | hsa-mir-187-3p | Down | NR2F1 | 2 | hsa-mir-149-5p |
| Up | ALPL | 1 | hsa-mir-204-5p | Down | **CA3** | 2 | hsa-mir-1-3p |
| Up | CCL3L3 | 1 | hsa-mir-215-5p | Down | CSNK2A2 | 2 | hsa-mir-1228-3p |
| Up | LMO2 | 1 | hsa-mir-223-3p | Down | HERC5 | 2 | hsa-mir-3529-3p |
| Up | ADAMTSL4 | 1 | hsa-mir-1-3p | Down | PXMP2 | 2 | hsa-mir-15b-5p |
| Up | TNFRSF25 | 1 | hsa-mir-124-3p | Down | SULF1 | 2 | hsa-mir-516a-3p |
| Up | MVP | 1 | hsa-mir-124-3p | Down | ABTB1 | 2 | hsa-mir-125b-5p |
| Up | PEMT | 1 | hsa-mir-124-3p | Down | IL13RA1 | 2 | hsa-mir-143-3p |
| Up | UNC13C | 1 | hsa-mir-128-3p | Down | BCKDHA | 2 | hsa-mir-29c-3p |
| Up | IL1RN | 1 | hsa-mir-125a-5p | Down | ZDHHC11 | 2 | hsa-mir-375 |
| Up | CFH | 1 | hsa-mir-146a-5p | Down | UBR2 | 2 | hsa-mir-5580-3p |
| Up | CARMIL1 | 1 | hsa-mir-149-5p | Down | GSDMB | 2 | hsa-mir-5692a |
| Up | RARRES3 | 1 | hsa-mir-30e-3p | Down | AASS | 2 | hsa-mir-591 |
| Up | AQP11 | 1 | hsa-mir-375 | Down | AGT | 1 | hsa-mir-26b-5p |
| Up | ESPNL | 1 | hsa-mir-375 | Down | SRPX | 1 | hsa-mir-26b-5p |
| Up | ZNF404 | 1 | hsa-mir-375 | Down | C17orf53 | 1 | hsa-mir-26b-5p |
| Up | CALB2 | 1 | hsa-mir-335-5p | Down | GCHFR | 1 | hsa-mir-92a-3p |
| Up | CHI3L2 | 1 | hsa-mir-335-5p | Down | TTC32 | 1 | hsa-mir-92a-3p |
| Up | COL11A1 | 1 | hsa-mir-335-5p | Down | FAM207A | 1 | hsa-mir-107 |
| Up | MGAT3 | 1 | hsa-mir-335-5p | Down | ZHX2 | 1 | hsa-mir-139-5p |
| Up | PLTP | 1 | hsa-mir-335-5p | Down | PLCL2 | 1 | hsa-mir-181a-5p |
| Up | PTGDS | 1 | hsa-mir-335-5p | Down | MACROD1 | 1 | hsa-mir-1-3p |
| Up | CLEC3B | 1 | hsa-mir-335-5p | Down | HYI | 1 | hsa-mir-1-3p |
| Up | LMOD1 | 1 | hsa-mir-335-5p | Down | PPP1R16A | 1 | hsa-mir-1-3p |
| Up | CLIP3 | 1 | hsa-mir-335-5p | Down | CALCRL | 1 | hsa-mir-124-3p |
| Up | SLC2A10 | 1 | hsa-mir-335-5p | Down | CMTM7 | 1 | hsa-mir-124-3p |
| Up | RSPO3 | 1 | hsa-mir-335-5p | Down | AZGP1 | 1 | hsa-mir-128-3p |
| Up | TMEM178A | 1 | hsa-mir-335-5p | Down | CASQ2 | 1 | hsa-mir-128-3p |
| Up | GPBAR1 | 1 | hsa-mir-335-5p | Down | C9orf72 | 1 | hsa-mir-142-3p |
| Up | SULT1A3 | 1 | hsa-mir-18a-3p | Down | MOCS1 | 1 | hsa-mir-320a |
| Up | MBP | 1 | hsa-mir-127-5p | Down | FBLN7 | 1 | hsa-mir-377-3p |
| Up | EFEMP1 | 1 | hsa-mir-338-5p | Down | GPR34 | 1 | hsa-mir-335-5p |
|  |  |  |  | Down | HLA-DMA | 1 | hsa-mir-335-5p |
|  |  |  |  | Down | SFTPD | 1 | hsa-mir-335-5p |
|  |  |  |  | Down | NAALAD2 | 1 | hsa-mir-335-5p |
|  |  |  |  | Down | CPAMD8 | 1 | hsa-mir-335-5p |
|  |  |  |  | Down | YPEL5 | 1 | hsa-mir-335-5p |
|  |  |  |  | Down | ZNF334 | 1 | hsa-mir-335-5p |
|  |  |  |  | Down | ACSS2 | 1 | hsa-mir-335-5p |
|  |  |  |  | Down | STOX1 | 1 | hsa-mir-335-5p |
|  |  |  |  | Down | COQ8A | 1 | hsa-mir-484 |
|  |  |  |  | Down | TMEM243 | 1 | hsa-mir-484 |
|  |  |  |  | Down | HACD1 | 1 | hsa-mir-652-3p |
|  |  |  |  | Down | LARP6 | 1 | hsa-mir-652-3p |
|  |  |  |  | Down | PPM1M | 1 | hsa-mir-744-5p |
|  |  |  |  | Down | CMTM8 | 1 | hsa-mir-877-5p |
|  |  |  |  | Down | GLYCTK | 1 | hsa-mir-940 |
|  |  |  |  | Down | FHOD3 | 1 | hsa-mir-1303 |
|  |  |  |  | Down | HDDC3 | 1 | hsa-mir-4708-3p |
|  |  |  |  | Down | DTNA | 1 | hsa-mir-8485 |

Degree – No of miRNA interact with target gene. We taken any one miRNA in table.

**Table 9** Target gene - miRNA interaction table

| **Regulation** | **TF** | **Degree** | **Target gene** | **Regulation** | **TF** | **Degree** | **Target gene** |
| --- | --- | --- | --- | --- | --- | --- | --- |
| Up | MAZ | 70 | YWHAH | Down | TFDP1 | 91 | EFNA1 |
| Up | TFDP1 | 62 | LYZ | Down | MAZ | 85 | MED16 |
| Up | KLF9 | 60 | HP | Down | KDM5B | 82 | RWDD2A |
| Up | KLF16 | 54 | TRAM2 | Down | SAP30 | 82 | ADD3 |
| Up | EZH2 | 51 | CCND1 | Down | PHF8 | 82 | AIP |
| Up | MXD4 | 49 | OAF | Down | SMAD5 | 77 | CCNF |
| Up | SAP30 | 49 | CCDC107 | Down | ZNF76 | 72 | ANG |
| Up | PHF8 | 49 | CEP19 | Down | ZNF580 | 69 | DBP |
| Up | KDM5B | 49 | HOXB8 | Down | SP1 | 68 | BCKDHA |
| Up | HBP1 | 48 | TNFRSF12A | Down | ZNF24 | 66 | HMGB2 |
| Up | ZNF580 | 48 | BAMBI | Down | ELF1 | 65 | GADD45B |
| Up | ZNF76 | 48 | CRABP2 | Down | GTF2E2 | 65 | ZFAND1 |
| Up | SIN3A | 47 | STAT3 | Down | ZFP2 | 60 | SERTAD4 |
| Up | ZNF24 | 47 | SH3BGRL3 | Down | POLR2A | 60 | KHDRBS1 |
| Up | NR2F6 | 45 | NRCAM | Down | KLF16 | 59 | FBXO46 |
| Up | MXD3 | 45 | OSGIN1 | Down | HBP1 | 58 | LSS |
| Up | ATF1 | 44 | LEP | Down | CREB3L1 | 58 | KAT8 |
| Up | ZBTB11 | 44 | HSPA8 | Down | CHD1 | 58 | EIF4B |
| Up | ZNF423 | 44 | TUBB2B | Down | KLF7 | 57 | CTH |
| Up | NRF1 | 43 | DBNDD1 | Down | REST | 56 | MIS18BP1 |
| Up | SSRP1 | 42 | PLTP | Down | ATF1 | 56 | CCNB1IP1 |
| Up | TGIF2 | 42 | FAM98A | Down | IRF1 | 56 | TRIM24 |
| Up | GLIS2 | 42 | TAGLN2 | Down | ZNF501 | 55 | HNRNPA1P10 |
| Up | ZBTB7A | 41 | ADAMTSL4 | Down | MYNN | 55 | FOXO3 |
| Up | ELF1 | 41 | NCKAP5L | Down | ZBTB11 | 54 | MOCS1 |
| Up | ZBTB26 | 40 | MT2A | Down | ZBTB7A | 54 | FOXO1 |
| Up | SMAD5 | 40 | MYEOV | Down | KLF11 | 53 | HLF |
| Up | ARID4B | 39 | TLE2 | Down | ZNF71 | 53 | NR2F1 |
| Up | KLF11 | 38 | PLEKHO2 | Down | SSRP1 | 52 | HLA-DMA |
| Up | RXRB | 38 | CFB | Down | TAF7 | 52 | ZNF581 |
| Up | ZBTB33 | 38 | CD68 | Down | NR2F6 | 52 | ANKRD37 |
| Up | NR4A1 | 38 | FTH1 | Down | HMGN3 | 51 | BCAT2 |
| Up | TFE3 | 38 | B4GALT5 | Down | ZFX | 51 | H1F0 |
| Up | ZFX | 38 | NEK6 | Down | ZNF324 | 51 | PTEN |
| Up | SOX13 | 37 | INF2 | Down | ZEB1 | 51 | GCHFR |
| Up | HIC1 | 37 | AIFM2 | Down | MXD3 | 50 | LETMD1 |
| Up | DMAP1 | 36 | ABCC3 | Down | TGIF2 | 50 | H1FX |
| Up | SP2 | 36 | FGF11 | Down | KLF8 | 50 | KLF8 |
| Up | THRB | 36 | AEN | Down | NRF1 | 49 | ORMDL3 |
| Up | NR2F1 | 36 | NANOS3 | Down | BCL11B | 49 | HOXA10 |
| Up | KLF1 | 36 | ELMOD3 | Down | BCL11A | 48 | FBL |
| Up | IRF1 | 36 | RHOC | Down | MXD4 | 47 | LDHD |
| Up | GATAD1 | 36 | SULT1A1 | Down | KLF1 | 47 | KLF9 |
| Up | ZFP37 | 36 | LOX | Down | ZFP37 | 47 | BCKDHA |
| Up | BCOR | 36 | ERV3-1 | Down | ZNF335 | 45 | AASS |
| Up | ZFP64 | 35 | HNMT | Down | WT1 | 45 | ELF2 |
| Up | MYNN | 34 | MARCKS | Down | ZNF423 | 45 | MRPL55 |
| Up | CHD1 | 34 | IRX2 | Down | SMARCA5 | 45 | NASP |
| Up | DRAP1 | 34 | QPRT | Down | E2F5 | 45 | FADS1 |
| Up | BCL11B | 34 | TYMP | Down | SP2 | 45 | MSRB1 |
| Up | HDGF | 34 | S100A3 | Down | NFRKB | 45 | CDKN1B |
| Up | ZNF2 | 34 | VLDLR | Down | ZNF610 | 44 | HDDC3 |
| Up | HMG20B | 33 | SOS1 | Down | NFYC | 44 | TTC38 |
| Up | MBD1 | 33 | ZNF219 | Down | ID3 | 43 | EPB41L4A-AS1 |
| Up | GTF2E2 | 33 | TM4SF19 | Down | ETV4 | 43 | ERRFI1 |
| Up | WRNIP1 | 33 | LRRC32 | Down | DMAP1 | 42 | RNF125 |
| Up | MLLT1 | 33 | CCND2 | Down | EGR1 | 42 | FOXN2 |
| Up | SP1 | 33 | SLC7A4 | Down | SP3 | 42 | EYA1 |
| Up | CTCF | 33 | ALCAM | Down | IRF4 | 42 | SIN3A |
| Up | KLF8 | 33 | NOTCH3 | Down | FOXM1 | 42 | RFX1 |
| Up | ETV4 | 33 | MXRA7 | Down | WRNIP1 | 42 | ST3GAL6 |
| Up | TEAD1 | 32 | NCKAP5L | Down | FOXJ2 | 42 | UBR2 |
| Up | SMAD4 | 32 | CD248 | Down | NR2C2 | 41 | UBE2W |
| Up | REST | 32 | CDR2L | Down | ZBTB26 | 41 | ZHX2 |
| Up | NR2C2 | 32 | CCPG1 | Down | INSM2 | 41 | S100A1 |
| Up | EGR1 | 32 | FSTL3 | Down | ZNF639 | 40 | LIX1L |
| Up | ZNF610 | 32 | LMOD1 | Down | SP7 | 40 | LAPTM4B |
| Up | IRF4 | 32 | CLIP3 | Down | PPARG | 40 | MARCH3 |
| Up | FOXM1 | 32 | COL16A1 | Down | GABPA | 40 | INTS6 |
| Up | GATA4 | 32 | AQP11 | Down | YY1 | 39 | PER2 |
| Up | KLF7 | 32 | CALU | Down | HCFC1 | 39 | ORC2 |
| Up | ZNF501 | 32 | RSPO3 | Down | BCOR | 39 | MED28 |
| Up | ELK1 | 32 | TPM2 | Down | ARID4B | 39 | PCM1 |
| Up | ZFP2 | 32 | CD9 | Down | GATAD1 | 38 | TXNIP |
| Up | KDM1A | 31 | KIAA1671 | Down | CTCF | 38 | WNT11 |
| Up | E2F5 | 31 | DOCK6 | Down | CREM | 38 | MLX |
| Up | POLR2A | 31 | HBB | Down | ELF3 | 38 | NR3C1 |
| Up | SMARCA5 | 31 | GPNMB | Down | CCNT2 | 38 | RBMX |
| Up | FOSL1 | 31 | LAMB2 | Down | NR4A1 | 37 | CISH |
| Up | CREB3L1 | 31 | ZCCHC24 | Down | RFXANK | 37 | LDHD |
| Up | TFAP4 | 30 | DLGAP4 | Down | KLF6 | 37 | CD55 |
| Up | RCOR2 | 30 | GLS | Down | NCOR1 | 37 | RBM15 |
| Up | ZNF341 | 30 | KLHL30 | Down | HMG20B | 36 | CABLES1 |
| Up | ZNF175 | 30 | CXCL10 | Down | ZNF101 | 36 | PCCA |
| Up | ZEB1 | 30 | EMX2 | Down | SMAD4 | 36 | SIVA1 |
| Up | BCL11A | 30 | C4orf48 | Down | GTF2F1 | 36 | STAG2 |
| Up | SOX5 | 29 | ERBB2 | Down | POLR2H | 36 | RBBP4 |
| Up | ZNF644 | 29 | LBP | Down | TFAP4 | 35 | DCAF7 |
| Up | ZNF589 | 29 | HSD11B1 | Down | GATA4 | 35 | CSNK2B |
| Up | ZNF197 | 29 | DAG1 | Down | TRIM28 | 35 | ZC2HC1A |
| Up | RERE | 29 | ENC1 | Down | MBD1 | 35 | DNAJC27 |
| Up | PPARG | 29 | CRABP2 | Down | HIC1 | 35 | ECI1 |
| Up | NFRKB | 29 | SKAP2 | Down | KDM5A | 35 | GMPS |
| Up | RAD21 | 29 | SULT1A2 | Down | RXRB | 35 | MT1G |
| Up | RFXANK | 29 | DLGAP4 | Down | GMEB2 | 35 | YPEL5 |
| Up | INSM2 | 29 | MVP | Down | ZNF644 | 35 | CD302 |
| Up | TEAD3 | 28 | WWC1 | Down | KLF13 | 35 | ZNF394 |
| Up | HMGN3 | 27 | PPDPF | Down | ZNF18 | 35 | ZNF334 |
| Up | CBFB | 27 | LCP1 | Down | GLIS2 | 33 | PPP1R1B |
| Up | SP3 | 27 | GPR137B | Down | ZBTB33 | 33 | STOX1 |
| Up | ZNF639 | 27 | HSPA5 | Down | L3MBTL2 | 33 | THYN1 |
| Up | ID3 | 27 | MMP9 | Down | ZBTB40 | 33 | RNF144A |
| Up | RARA | 26 | MFAP4 | Down | ESRRA | 33 | ZFAND5 |
| Up | IKZF1 | 26 | S100A4 | Down | CBFB | 33 | CBFB |
| Up | ZNF71 | 26 | MN1 | Down | NCOA1 | 33 | ZNF280D |
| Up | NFYC | 26 | SOD2 | Down | TFE3 | 33 | C17orf53 |
| Up | CEBPG | 26 | CDK14 | Down | TEAD3 | 32 | RPS2 |
| Up | NFIA | 25 | LAMA5 | Down | TARDBP | 32 | RPS3 |
| Up | EED | 25 | IRF8 | Down | MLLT1 | 32 | STARD13 |
| Up | MTA2 | 25 | SAA1 | Down | HDGF | 32 | RPS12 |
| Up | SP7 | 25 | HOXC8 | Down | ETV1 | 32 | SRSF4 |
| Up | GABPA | 25 | C3orf14 | Down | ZNF2 | 32 | AUTS2 |
| Up | NFIL3 | 25 | NQO1 | Down | ATF3 | 31 | RHOT1 |
| Up | SIRT6 | 24 | TNFRSF12A | Down | SOX13 | 31 | GPHN |
| Up | SMARCA4 | 24 | SMYD2 | Down | ZNF341 | 31 | RPS9 |
| Up | GATAD2A | 24 | DBNDD1 | Down | RNF2 | 31 | SOCS2 |
| Up | ELF3 | 23 | SLX1A-SULT1A3 | Down | SOX5 | 31 | PCYT2 |
| Up | FOSL2 | 23 | PALLD | Down | MEF2D | 31 | RTN2 |
| Up | MLX | 23 | PLTP | Down | GLI4 | 31 | NDUFV3 |
| Up | NCOR1 | 23 | LCP1 | Down | EZH2 | 31 | TWIST1 |
| Up | ZNF394 | 23 | TYROBP | Down | ZFP64 | 30 | DCAF7 |
| Up | EBF1 | 23 | VASN | Down | TRIM22 | 30 | FAM207A |
| Up | WT1 | 23 | NAV1 | Down | ETS1 | 30 | SPSB3 |
| Up | TAF7 | 23 | CYR61 | Down | TSHZ1 | 30 | NR2F1 |
| Up | ZNF324 | 23 | RAB9A | Down | THRB | 29 | PPM1M |
| Up | CTBP2 | 23 | FAT1 | Down | LEF1 | 29 | CSRNP1 |
| Up | HDAC1 | 23 | SH3BGRL3 | Down | DDX20 | 29 | DBP |
| Up | HMG20A | 22 | SAA4 | Down | CEBPG | 29 | POLR1E |
| Up | ZKSCAN1 | 22 | SPARC | Down | USF2 | 29 | RPS27 |
| Up | ETV1 | 22 | SDSL | Down | NFIA | 29 | SPATA7 |
| Up | ZNF584 | 22 | RARRES3 | Down | DRAP1 | 29 | RNASE4 |
| Up | GTF2F1 | 22 | RND3 | Down | ZNF589 | 28 | MOCS1 |
| Up | L3MBTL2 | 22 | AMOT | Down | ZNF384 | 28 | USP13 |
| Up | BCL6 | 22 | HLA-A | Down | ZNF83 | 28 | SRSF3 |
| Up | ATF3 | 21 | CFH | Down | GATAD2A | 28 | TTC32 |
| Up | YY1 | 21 | BHMT2 | Down | KDM1A | 27 | ACSS2 |
| Up | SMARCE1 | 21 | MGST1 | Down | BCL6 | 27 | INTS6 |
| Up | LEF1 | 21 | LMO2 | Down | FOSL1 | 27 | RPL29 |
| Up | ZBTB40 | 21 | HBA1 | Down | RAD21 | 27 | TMEM100 |
| Up | DPF2 | 20 | PLBD2 | Down | ZNF263 | 27 | RPL35 |
| Up | ADNP | 20 | COL15A1 | Down | RARA | 27 | FKBP5 |
| Up | TRIM28 | 20 | CYCS | Down | GLIS1 | 27 | RUNDC3B |
| Up | CREM | 20 | CTHRC1 | Down | SMARCE1 | 27 | UBR2 |
| Up | KLF6 | 20 | LCP1 | Down | ZNF366 | 26 | TMOD1 |
| Up | ARID1B | 20 | SLC35G1 | Down | ZNF175 | 26 | TXNIP |
| Up | FOXA3 | 19 | HAVCR2 | Down | ZNF382 | 26 | NASP |
| Up | GLIS1 | 19 | FGF11 | Down | RCOR2 | 26 | ORMDL3 |
| Up | SUZ12 | 19 | SFRP4 | Down | PRDM10 | 26 | AZIN1 |
| Up | NR2F2 | 19 | INF2 | Down | SIRT6 | 25 | TGIF1 |
| Up | THAP1 | 19 | MYL9 | Down | SMARCA4 | 25 | BLVRB |
| Up | ZNF335 | 19 | EGR2 | Down | RERE | 25 | RPL29 |
| Up | BHLHE40 | 19 | TAGLN2 | Down | ZNF197 | 25 | GMPS |
| Up | TRIM22 | 19 | CHI3L2 | Down | MTA1 | 25 | CNBP |
| Up | GATA2 | 19 | TRAM2 | Down | ADNP | 25 | FBL |
| Up | ESRRA | 19 | TLE2 | Down | ZKSCAN1 | 24 | RPL18 |
| Up | POLR2H | 18 | TDP1 | Down | NFIL3 | 24 | AHCTF1 |
| Up | FOXJ2 | 18 | APLNR | Down | JUND | 24 | DTNA |
| Up | ZNF18 | 18 | HOXC8 | Down | ARID1B | 23 | GLUL |
| Up | CUX1 | 18 | TMED3 | Down | PBX2 | 22 | MRPL55 |
| Up | GFI1B | 18 | RAB31 | Down | ARNT | 22 | FADS1 |
| Up | ETS1 | 18 | NRP2 | Down | HDAC1 | 22 | TSC22D3 |
| Up | ZHX2 | 18 | GLS | Down | IKZF1 | 22 | RPL18A |
| Up | MEF2D | 18 | DBNDD1 | Down | GATA2 | 22 | GMCL1 |
| Up | ZNF121 | 17 | TYROBP | Down | MYBL2 | 21 | MT1G |
| Up | TBX21 | 17 | MSC | Down | MBD4 | 21 | GPHN |
| Up | ZNF263 | 16 | TIMP1 | Down | HNF4G | 21 | ACACB |
| Up | PRDM1 | 16 | RND3 | Down | CREB1 | 21 | ERRFI1 |
| Up | CREB1 | 16 | PYGL | Down | SIN3B | 21 | ALDH6A1 |
| Up | ZNF623 | 16 | ZNF404 | Down | EED | 21 | AIP |
| Up | ZNF382 | 15 | RSPO3 | Down | CTBP2 | 21 | SOX17 |
| Up | MTA1 | 15 | KLF4 | Down | IRF2 | 21 | CCDC59 |
| Up | SUPT5H | 15 | HMOX1 | Down | GTF2A2 | 21 | H1FX |
| Up | MBD2 | 15 | WISP2 | Down | FOXA3 | 21 | EEF2K |
| Up | RNF2 | 15 | PPP2R1B | Down | STAT1 | 21 | RSL24D1 |
| Up | JUND | 15 | OXT | Down | SCRT1 | 20 | ORC2 |
| Up | PBX2 | 15 | PPFIBP2 | Down | GFI1B | 20 | EYA1 |
| Up | HNF4G | 14 | OAF | Down | ZNF146 | 20 | LARP6 |
| Up | MYBL2 | 14 | LBP | Down | MXI1 | 20 | ANKRD37 |
| Up | MBD4 | 14 | SAA4 | Down | MBD2 | 20 | BTG2 |
| Up | TRIM24 | 14 | MYEOV | Down | MIXL1 | 20 | HNRNPA1P10 |
| Up | RELA | 14 | DLGAP4 | Down | CREB3 | 20 | BHLHE40 |
| Up | DDX20 | 14 | MIR3188 | Down | BACH1 | 20 | CISH |
| Up | GTF2A2 | 14 | CCDC80 | Down | ZNF623 | 20 | HLA-DMA |
| Up | SMC3 | 14 | CYB561 | Down | ZNF207 | 20 | YPEL5 |
| Up | TSHZ1 | 14 | CRIP1 | Down | SUZ12 | 20 | FAM107A |
| Up | TARDBP | 14 | MIR3188 | Down | SUPT5H | 19 | RPL35 |
| Up | PKNOX1 | 13 | TM4SF19 | Down | MAX | 19 | RPL18 |
| Up | KLF13 | 13 | TRAM2 | Down | JUNB | 19 | RPL14 |
| Up | TCF7 | 13 | FLNC | Down | HES1 | 19 | RHOT1 |
| Up | PRDM10 | 13 | FILIP1L | Down | TBX21 | 18 | ARHGEF3 |
| Up | BACH1 | 13 | RARRES3 | Down | TEAD1 | 18 | FOXN2 |
| Up | MIXL1 | 13 | TM4SF19 | Down | ZBTB17 | 18 | HOXA10 |
| Up | CREB3 | 13 | NUP188 | Down | HMBOX1 | 18 | RBMX |
| Up | GLI4 | 13 | MXRA7 | Down | ZNF584 | 18 | MOCS1 |
| Up | HCFC1 | 13 | PMEPA1 | Down | ZNF121 | 18 | SRSF7 |
| Up | CCNT2 | 12 | LAMA5 | Down | ZNF143 | 17 | ADD3 |
| Up | HMBOX1 | 12 | FAM189A2 | Down | PRDM1 | 17 | PTEN |
| Up | GMEB2 | 12 | TDP1 | Down | ATF4 | 17 | N4BP2L2 |
| Up | ARNT | 11 | CLDN15 | Down | HMG20A | 17 | NET1 |
| Up | ZNF143 | 11 | ERV3-1 | Down | RAD51 | 17 | PHF3 |
| Up | KDM5A | 11 | MMP9 | Down | RELA | 17 | LEFTY2 |
| Up | ZNF366 | 11 | IGFBP6 | Down | MYC | 17 | EIF4B |
| Up | RCOR1 | 11 | AQP11 | Down | EGR2 | 16 | TGIF1 |
| Up | MXI1 | 11 | DLGAP4 | Down | EBF1 | 16 | GPR146 |
| Up | ZNF207 | 11 | ZNF219 | Down | ZNF239 | 16 | BLVRB |
| Up | DEK | 11 | S100A4 | Down | PML | 16 | CSNK2B |
| Up | ZNF101 | 11 | CD68 | Down | THRAP3 | 16 | ELOVL6 |
| Up | HDAC6 | 11 | CXCL12 | Down | ZBTB1 | 16 | LSS |
| Up | HDAC2 | 11 | ESPNL | Down | THAP1 | 15 | AGT |
| Up | NCOA1 | 11 | CRTAP | Down | NR2F2 | 15 | KLF9 |
| Up | ZNF558 | 11 | TUBB2B | Down | KLF4 | 15 | WNT11 |
| Up | USF2 | 11 | KCNB1 | Down | MTA2 | 15 | STARD13 |
| Up | ZNF7 | 11 | ITGB5 | Down | ZNF558 | 15 | BCAT2 |
| Up | ZNF384 | 10 | LYZ | Down | SMC3 | 15 | C17orf53 |
| Up | SREBF2 | 10 | LAMB3 | Down | CDC5L | 15 | RPS27 |
| Up | RUNX3 | 10 | PLEKHO2 | Down | PKNOX1 | 14 | ZNF394 |
| Up | SPI1 | 10 | SH3BGRL3 | Down | TCF7 | 14 | PHF13 |
| Up | ZBTB1 | 10 | COL6A1 | Down | NFE2 | 14 | BHLHE40 |
| Up | MAFK | 10 | MVP | Down | SREBF2 | 13 | KHDRBS1 |
| Up | ZNF83 | 10 | RHOC | Down | FOXA2 | 13 | BEX4 |
| Up | MYC | 10 | VASN | Down | CEBPD | 13 | RNF144A |
| Up | NFIC | 10 | HNMT | Down | TEAD4 | 13 | RPS9 |
| Up | RAD51 | 10 | LEP | Down | MITF | 13 | NR4A2 |
| Up | CTBP1 | 9 | SOBP | Down | ZNF7 | 12 | GSDMB |
| Up | ZBTB17 | 9 | BAMBI | Down | RFX3 | 12 | MED16 |
| Up | CEBPA | 9 | TUBB2A | Down | DNMT1 | 12 | DBP |
| Up | ATF4 | 9 | C1S | Down | FOSL2 | 12 | RSBN1 |
| Up | TAL1 | 9 | SLC7A4 | Down | CTBP1 | 11 | FOXO3 |
| Up | RFX1 | 9 | STAT3 | Down | HDAC6 | 11 | PCM1 |
| Up | MAX | 9 | CCND2 | Down | HDAC2 | 11 | GADD45B |
| Up | STAT1 | 9 | FSTL3 | Down | RCOR1 | 11 | RPS27 |
| Up | RFX3 | 9 | TAGLN2 | Down | TBP | 11 | ANG |
| Up | SCRT1 | 8 | MAP1B | Down | ZNF641 | 11 | SIN3A |
| Up | THRAP3 | 8 | HBB | Down | ZNF407 | 11 | NXNL1 |
| Up | HHEX | 8 | ELMOD3 | Down | CUX1 | 11 | CKB |
| Up | GATA1 | 8 | RAB6B | Down | MAFK | 11 | LAPTM4B |
| Up | PRDM2 | 8 | C1R | Down | EP300 | 11 | RBMX |
| Up | NFE2 | 8 | SH3BGRL3 | Down | CEBPA | 11 | LEMD3 |
| Up | FOXK2 | 8 | ERBB2 | Down | GTF2B | 11 | RPS29 |
| Up | JUNB | 7 | TMEM98 | Down | CBX8 | 10 | PELI2 |
| Up | DNMT1 | 7 | TNFRSF12A | Down | FOXK2 | 10 | KLF9 |
| Up | ZNF146 | 7 | TUBB2B | Down | GATA1 | 10 | PLCL2 |
| Up | CDC5L | 7 | TUBB2A | Down | PRDM2 | 10 | PCYT2 |
| Up | TBX3 | 7 | HNMT | Down | HHEX | 9 | CTH |
| Up | FOS | 7 | VGLL3 | Down | ZHX1 | 9 | MKNK2 |
| Up | CEBPB | 6 | AIFM2 | Down | ZNF217 | 8 | TRIM33 |
| Up | ZNF407 | 6 | APLNR | Down | TAL1 | 8 | CD55 |
| Up | CEBPD | 6 | TRIM6 | Down | HDAC8 | 8 | HDDC3 |
| Up | FOXA2 | 6 | SPTAN1 | Down | E2F6 | 8 | PFKFB3 |
| Up | TEAD4 | 6 | BAMBI | Down | MNT | 8 | SIVA1 |
| Up | GATAD2B | 6 | STC2 | Down | DIDO1 | 8 | LEFTY2 |
| Up | MAFF | 6 | ENC1 | Down | DEK | 7 | RPL35 |
| Up | MITF | 6 | FTH1 | Down | SCRT2 | 7 | HSD17B12 |
| Up | SIN3B | 6 | MVP | Down | CEBPB | 7 | REV1 |
| Up | HES1 | 6 | LAMB3 | Down | PTRF | 7 | SLC3A2 |
| Up | ZNF512 | 6 | SCARB1 | Down | ZNF512 | 7 | CTSC |
| Up | SCRT2 | 6 | PLEKHO2 | Down | SPI1 | 7 | PPM1M |
| Up | GATA3 | 5 | HSPA5 | Down | RUNX3 | 7 | ELOVL6 |
| Up | E2F4 | 5 | MT2A | Down | MAFF | 7 | IGF1 |
| Up | ZNF239 | 5 | DBNDD1 | Down | GATAD2B | 7 | ZFAND1 |
| Up | NONO | 5 | MYEOV | Down | RUNX1 | 6 | KHDRBS1 |
| Up | JUN | 5 | HSPB8 | Down | SREBF1 | 6 | DPF2 |
| Up | IRF2 | 5 | IFIT2 | Down | PYGO2 | 6 | MRPL55 |
| Up | ZNF217 | 5 | MGST1 | Down | STAT3 | 6 | NDFIP2 |
| Up | TEAD2 | 5 | RARRES3 | Down | GATA3 | 6 | ZHX2 |
| Up | POU5F1 | 4 | ANGPT1 | Down | ZNF547 | 6 | GPHN |
| Up | ZNF641 | 4 | CCDC107 | Down | POU2F2 | 6 | CLDND1 |
| Up | POU2F2 | 4 | TAGLN2 | Down | TBX3 | 6 | PER2 |
| Up | PYGO2 | 4 | CHST3 | Down | ZNF202 | 5 | RWDD2A |
| Up | PTRF | 4 | NRCAM | Down | HNF4A | 5 | ORMDL3 |
| Up | SIX4 | 4 | PMEPA1 | Down | E2F4 | 5 | MIS18BP1 |
| Up | EP300 | 4 | FLNC | Down | CHD4 | 5 | RFX1 |
| Up | NFATC1 | 4 | NAV1 | Down | MAFG | 5 | UBR2 |
| Up | MTA3 | 4 | MIR3188 | Down | PTTG1 | 5 | BHLHE40 |
| Up | ZNF16 | 4 | DOCK6 | Down | ZNF585B | 5 | NR3C1 |
| Up | NFE2L2 | 3 | PRSS23 | Down | ZNF8 | 5 | FBL |
| Up | ZNF488 | 3 | HOXC8 | Down | NFYB | 5 | DBP |
| Up | PML | 3 | CD68 | Down | NFE2L2 | 5 | NDUFV3 |
| Up | ZHX1 | 3 | SLC7A4 | Down | POU5F1 | 4 | AASS |
| Up | BCL3 | 3 | CHI3L2 | Down | ATF2 | 4 | RBM15 |
| Up | ZNF547 | 3 | CDR2L | Down | NFIC | 4 | CTSC |
| Up | DIDO1 | 3 | COL6A1 | Down | IRF3 | 4 | BLVRB |
| Up | ZC3H11A | 3 | LAMA5 | Down | USF1 | 4 | EEF2K |
| Up | UBTF | 3 | CRIP1 | Down | ZNF16 | 4 | SRSF3 |
| Up | HLF | 3 | IGFBP6 | Down | ETS2 | 3 | TRIM24 |
| Up | USF1 | 3 | STC2 | Down | TBL1XR1 | 3 | SOCS2 |
| Up | ZNF264 | 3 | SOBP | Down | TEAD2 | 3 | PHF13 |
| Up | YBX1 | 3 | SLC22A12 | Down | ETV6 | 3 | ORC2 |
| Up | CBX8 | 2 | CCND2 | Down | CHD2 | 3 | PTEN |
| Up | HNF4A | 2 | CFB | Down | ZFP41 | 3 | GADD45B |
| Up | MYB | 2 | MYEOV | Down | TCF3 | 3 | LAPTM4B |
| Up | ZNF202 | 2 | TNFRSF12A | Down | STAT5A | 3 | RNF144A |
| Up | RBBP5 | 2 | GNG2 | Down | SETDB1 | 3 | RNF217 |
| Up | RXRA | 2 | PLTP | Down | RBBP5 | 3 | RPL9 |
| Up | ZNF8 | 2 | DBNDD1 | Down | CEBPZ | 3 | SIN3A |
| Up | SREBF1 | 2 | DHDDS | Down | POLR3A | 2 | ANG |
| Up | ATF2 | 2 | EMX2 | Down | BDP1 | 2 | RNASE4 |
| Up | ZNF140 | 2 | FGF11 | Down | BRF1 | 2 | ANG |
| Up | MCM3 | 2 | FGR | Down | GTF3C2 | 2 | RNASE4 |
| Up | KAT2A | 2 | SGK1 | Down | MCM7 | 2 | KCNK6 |
| Up | GTF2B | 2 | SOS1 | Down | NANOG | 2 | C17orf53 |
| Up | CHD7 | 2 | SLC2A5 | Down | JUN | 2 | POLR1D |
| Up | PTTG1 | 2 | HSPA5 | Down | ZNF138 | 2 | GINS3 |
| Up | EHMT2 | 2 | KCNB1 | Down | NFYA | 2 | RGMB |
| Up | RUNX1 | 2 | MIR3188 | Down | TAF1 | 2 | HNRNPA1P10 |
| Up | ZNF274 | 2 | RHOC | Down | SP4 | 2 | ZNF394 |
| Up | AEBP2 | 2 | SGCE | Down | PRDM12 | 2 | RPL17 |
| Up | NANOG | 2 | SH3BGRL3 | Down | YBX1 | 1 | BCAT2 |
| Up | HDAC8 | 2 | SLC7A4 | Down | HSF1 | 1 | CCDC59 |
| Up | ZNF585B | 2 | TYMP | Down | PPARGC1A | 1 | CCDC59 |
| Up | MEF2A | 1 | CHI3L2 | Down | NONO | 1 | CD55 |
| Up | MEF2C | 1 | CHI3L2 | Down | FOS | 1 | CD55 |
| Up | ZNF292 | 1 | COL12A1 | Down | SIX4 | 1 | DCAF7 |
| Up | NFE2L1 | 1 | DBNDD1 | Down | TCF7L2 | 1 | ELK1 |
| Up | IRF3 | 1 | DHDDS | Down | MYB | 1 | FOXO1 |
| Up | SRF | 1 | EGR2 | Down | ZNF707 | 1 | GCHFR |
| Up | TBP | 1 | FTH1 | Down | ZC3H11A | 1 | GCHFR |
| Up | TBL1XR1 | 1 | FTH1 | Down | UBTF | 1 | GCHFR |
| Up | NR3C1 | 1 | MYEOV | Down | KAT2A | 1 | H1FX |
| Up | TCF7L2 | 1 | SERPINH1 | Down | CHD7 | 1 | LDHC |
| Up | ETS2 | 1 | SLC7A4 | Down | SRF | 1 | MRPL55 |
| Up | MCM5 | 1 | SLC7A4 | Down | ZZZ3 | 1 | NR2F1 |
| Up | E2F6 | 1 | SLC7A4 | Down | RXRA | 1 | ORMDL3 |
| Up | TSC22D4 | 1 | SLC7A4 | Down | ZNF264 | 1 | PTEN |
| Up | MCM7 | 1 | SLC7A4 | Down | ZNF274 | 1 | RAMP2 |
| Up | ETV6 | 1 | SOS1 | Down | BRCA1 | 1 | RPS2 |
| Up | STAT5A | 1 | SPTAN1 | Down | FOXA1 | 1 | UBR2 |
| Up | TCF3 | 1 | SPTAN1 | Down | NFE2L1 | 1 | ZFAND5 |
| Up | MAFG | 1 | TPGS2 | Down | BCL3 | 1 | ZNF581 |
| Up | TAF1 | 1 | TUBB2B | Down | BCLAF1 | 1 | ZNF581 |
| Up | CHD4 | 1 | TYROBP | Down |  |  |  |
| Up | MNT | 1 | VASN | Down |  |  |  |
| Up | SETDB1 | 1 | ZNF404 | Down |  |  |  |

Degree – No of target gene interact with TF. We taken any one target gene in table. TF transcription factors

**Table 10** Docking results of Designed Molecules on ERBB2, HSPAB 8 and STAT 3 Proteins

| **Sl. No/**  **Code** | **ERBB2** | | | **HSPAB 8** | | | **STAT 3** | | |
| --- | --- | --- | --- | --- | --- | --- | --- | --- | --- |
|  | **PDB: 1MFL** | | | **PDB: 5OOW** | | | **PDB: 3CWG** | | |
|  | **Total Score** | **Crash**  **(-Ve)** | **Polar** | **Total Score** | **Crash**  **(-Ve)** | **Polar** | **Total Score** | **Crash**  **(-Ve)** | **Polar** |
| PM1 | 6.354 | 1.8554 | 3.309 | 7.3649 | 0.9447 | 4.2486 | 6.418 | 1.1375 | 3.1553 |
| PM 2 | 5.9858 | 0.8123 | 2.926 | 6.6656 | 1.4583 | 2.4965 | 6.0481 | 1.6073 | 1.7502 |
| PM 3 | 6.1052 | 0.8765 | 3.3637 | 7.2703 | 1.3253 | 2.7246 | 6.1269 | 1.4193 | 0.4039 |
| PM 4 | 6.187 | 1.1189 | 4.0929 | 7.0949 | 0.8684 | 3.9023 | 7.1631 | 0.6057 | 3.5534 |
| PM 5 | 6.4935 | 1.214 | 2.7323 | 7.1414 | 0.6499 | 4.4987 | 6.8638 | 1.3073 | 2.1592 |
| PM 6 | 6.0433 | 1.1152 | 1.6313 | 6.9984 | 0.9305 | 3.2943 | 8.8312 | 2.3431 | 2.9687 |
| PM 7 | 5.0229 | 1.0973 | 2.2752 | 7.6405 | 1.843 | 3.8252 | 6.6734 | 1.5935 | 2.9562 |
| PM 8 | 5.5644 | 1.1034 | 3.7177 | 7.0861 | 0.945 | 3.7357 | 6.6148 | 1.2938 | 3.5281 |
| ND 1 | 5.0388 | 1.0515 | 1.1439 | 7.5269 | 1.5161 | 3.2626 | 7.3781 | 1.5294 | 0.6972 |
| ND 2 | 5.4742 | 0.9988 | 2.1351 | 7.5884 | 1.6485 | 2.6705 | 6.7647 | 1.8212 | 2.7292 |
| ND 3 | 5.5589 | 0.943 | 1.2409 | 7.6271 | 1.6791 | 2.6689 | 5.9477 | 0.8576 | 2.1241 |
| ND 4 | 7.2426 | 1.2189 | 3.8473 | 7.6963 | 1.7908 | 2.7594 | 6.7785 | 1.1241 | 2.6272 |
| ND 5 | 6.8014 | 1.6106 | 4.9544 | 8.0824 | 0.792 | 4.1267 | 7.9872 | 1.8386 | 1.7006 |
| ND 6 | 6.0228 | 0.9033 | 4.2139 | 7.6595 | 1.0783 | 3.3909 | 7.9567 | 2.8125 | 1.4927 |
| ND 7 | 6.0592 | 0.6791 | 2.9402 | 7.6766 | 1.7443 | 2.7273 | 6.7595 | 1.6798 | 2.4524 |
| ND 8 | 5.5869 | 2.0833 | 1.9807 | 7.1896 | 1.1333 | 3.0863 | 6.665 | 1.1301 | 2.4612 |
| FU 1 | 5.4199 | 0.3016 | 4.408 | 6.3822 | 0.9483 | 2.4809 | 6.9877 | 1.0049 | 1.7564 |
| FU 2 | 5.2033 | 0.6796 | 3.8339 | 6.4237 | 0.7108 | 2.6262 | 5.0806 | 2.4171 | 2.6277 |
| FU 3 | 5.6153 | 0.7548 | 3.5971 | 6.6718 | 0.8622 | 2.5807 | 5.0525 | 0.7271 | 1.0867 |
| FU 4 | 5.787 | 1.1682 | 3.4617 | 6.366 | 1.1302 | 2.7642 | 5.8162 | 1.3719 | 2.5882 |
| FU 5 | 7.6599 | 1.5352 | 6.1528 | 7.9335 | 0.8532 | 3.9764 | 6.4335 | 1.3482 | 3.8879 |
| FU 6 | 6.3452 | 0.8782 | 3.3486 | 7.3296 | 0.6103 | 4.8209 | 6.1884 | 1.8022 | 3.5115 |
| FU 7 | 5.3509 | 0.5639 | 3.6504 | 6.8527 | 0.8899 | 4.5422 | 6.2097 | 1.7944 | 2.2888 |
| FU 8 | 5.0006 | 0.6207 | 2.5575 | 7.5527 | 0.9242 | 3.4962 | 5.0749 | 0.7914 | 2.0186 |
| PF 1 | 6.3205 | 1.162 | 1.8661 | 7.1066 | 1.0973 | 2.6473 | 6.5213 | 1.5027 | 2.4556 |
| PF 2 | 5.8631 | 1.3064 | 5.1615 | 6.4083 | 0.8633 | 3.8894 | 6.721 | 1.3901 | 1.1491 |
| PF 3 | 5.0508 | 1.2347 | 1.0905 | 6.6714 | 0.8968 | 2.7028 | 6.348 | 1.1353 | 2.2938 |
| PF 4 | 5.8653 | 2.1412 | 2.2011 | 6.0181 | 0.9905 | 2.7088 | 7.0213 | 1.6703 | 4.1421 |
| PF 5 | 7.8421 | 1.4657 | 6.2584 | 7.0792 | 1.7372 | 4.7215 | 7.0386 | 1.6376 | 2.8817 |
| PF 6 | 6.2656 | 1.1912 | 4.2728 | 7.2659 | 1.1564 | 3.7669 | 6.6062 | 3.1043 | 2.8086 |
| PF 7 | 5.4077 | 0.9624 | 3.8651 | 7.0624 | 1.1262 | 2.4922 | 6.2014 | 1.8296 | 2.5331 |
| PF 8 | 6.1915 | 0.845 | 4.0836 | 6.4717 | 0.6029 | 4.3112 | 6.1099 | 1.1815 | 2.1838 |
| STD | 6.4695 | 2.2625 | 2.4422 | 8.2777 | 3.4553 | 1.9003 | 9.9563 | 1.9732 | 2.2073 |

* R’ - PM: P-Methoxy derivatives, ND: P- Dimethyl amino Derivatives FU: Furan Derivatives, PF: P- Fluoro derivatives & STD: Standard Orlistst
